# Darobactins Exhibiting Superior Antibiotic Activity by Cryo‐EM Structure Guided Biosynthetic Engineering**

**DOI:** 10.1002/anie.202214094

**Published:** 2022-12-07

**Authors:** Carsten E. Seyfert, Christoph Porten, Biao Yuan, Selina Deckarm, Fabian Panter, Chantal D. Bader, Janetta Coetzee, Felix Deschner, Kamaleddin H. M. E. Tehrani, Paul G. Higgins, Harald Seifert, Thomas C. Marlovits, Jennifer Herrmann, Rolf Müller

**Affiliations:** ^1^ Microbial Natural Products Helmholtz Institute for Pharmaceutical Research Saarland (HIPS) Helmholtz Centre for Infection Research (HZI) Department of Pharmacy at Saarland University Campus Building E8.1, 66123 Saarbrücken (Germany); ^2^ German Centre for Infection Research (DZIF), partnersite Hannover-Braunschweig Germany; ^3^ University Medical Center Hamburg-Eppendorf (UKE) Institute of Structural and Systems Biology Notkestraße 85, Building 15 22607 Hamburg Germany; ^4^ Centre for Structural Systems Biology (CSSB) Hamburg Germany; ^5^ Deutsches Elektronen-Synchrotron Zentrum (DESY) Hamburg Germany; ^6^ Microbiology, Immunology and Hygiene Faculty of Medicine and University Hospital Cologne University of Cologne Cologne Germany; ^7^ German Center for Infection Research (DZIF), partner site Bonn-Cologne Germany

**Keywords:** Antibiotics, BamA, CRAB, Cryo-Electron Microscopy, Natural Products

## Abstract

Over recent decades, the pipeline of antibiotics acting against Gram‐negative bacteria is running dry, as most discovered candidate antibiotics suffer from insufficient potency, pharmacokinetic properties, or toxicity. The darobactins, a promising new small peptide class of drug candidates, bind to novel antibiotic target BamA, an outer membrane protein. Previously, we reported that biosynthetic engineering in a heterologous host generated novel darobactins with enhanced antibacterial activity. Here we utilize an optimized purification method and present cryo‐EM structures of the Bam complex with darobactin 9 (D9), which served as a blueprint for the biotechnological generation of twenty new darobactins including halogenated analogs. The newly engineered darobactin 22 binds more tightly to BamA and outperforms the favorable activity profile of D9 against clinically relevant pathogens such as carbapenem‐resistant *Acinetobacter baumannii* up to 32‐fold, without observing toxic effects.

## Graphical abstract

Structural evaluation of the Bam complex with darobactin 9 using cryo‐EM combined with bioactivity evaluation led to biosynthetic engineering of novel derivatives with enhanced activity against Gram‐negative pathogens, including clinically relevant isolates. The currently most promising derivative darobactin 22 verified the structure and activity guided approach, exhibiting up to 128‐fold increased activity compared to natural darobactin A.

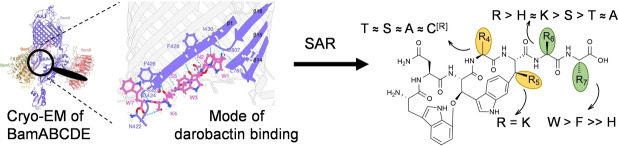

## Introduction

The stalling development of new antibiotics in combination with an increasing human population density and rising antimicrobial resistance (AMR) in bacteria leads to an emerging antibiotic crisis across the globe.[^1^, ^2^] Recent studies estimate that 1.27 million deaths in 2019 were attributed to bacterial AMR alone, while multidrug‐resistant bacterial infections contributed to 5 million deaths in already diseased patients.^[3]^ Moreover, during the current coronavirus (COVID‐19) pandemic, the viral infections were often accompanied by simultaneous infection with bacterial pathogens.[^4^, ^5^] Particularly, co‐infections with carbapenemase‐producing pathogens increase lethality rates in patients and contributed to the death of over 6 million COVID‐19 patients since the pandemic outbreak in 2020.^[6]^ The challenging research towards discovery and development of new antibiotic classes, especially against Gram‐negative pathogens causing life‐threatening infections, rarely results in promising candidates.[^2^, ^7^] Consequently, the demand for new drugs against pathogenic Gram‐negative bacteria, such as *Escherichia coli* (*E. coli*), *Acinetobacter baumannii* (*A. baumannii*), *Klebsiella pneumoniae* (*K. pneumoniae*), and *Pseudomonas aeruginosa* (*P. aeruginosa*) continuously rises.[^5^, ^8^]

Presently, there are some candidates in pre‐clinical and clinical development stages for the treatment of infections caused by Gram‐negative pathogens. However, these mostly belong to already known compound classes or they are novel chemical entities attacking common targets, such as the broad‐spectrum ribosome inhibitor odilorhabdin, originally produced by *Xenorhabdus nematophila*.^[9]^ The darobactins, which are also produced by an entomopathogenic bacterium, *Photorhabdus khanii*,^[10]^ however, are some of the most promising new antibacterials in the last 50 years^[2]^ given they address a novel broad‐spectrum target in Gram‐negative pathogens. The mechanism of action overcomes common pathogenic resistance and is effective, not only in vitro, but also in vivo.^[2]^ This family of bicyclic heptapeptides belongs to the biosynthetic class of ribosomally synthesized and post‐translationally modified peptides (RiPPs).^[10]^ Darobactins distinguish themselves from other new antibiotic candidates, such as the recently published macolacin,^[11]^ as they do not affect human gut commensals of the family *Bacteriodes*, a beneficial characteristic considering the intended use in humans.^[10]^ Due to the selective binding to the β‐barrel of the outer membrane protein (OMP) BamA, darobactin interferes with the integration of natural OMPs into the membrane by mimicking the OMP signaling peptide.^[12]^ As a consequence, BamA is unable to close the lateral gate and, thus, affects mechanical, kinetic, and energetic properties of the entire complex.[^13^, ^14^] Highlighting the value of BamA as a worthwhile antibacterial target, darobactin‐resistant *E. coli* strains with mutated BamA generated in vitro resulted in substantially reduced fitness and virulence. This indicates a lack of pre‐existing resistance and a reduced risk of fast resistance development, at least in *E.coli*.[^12^, ^15^, ^16^]

Darobactin has only been produced in low amounts in the native host bacterium, insufficient for larger industrial fermentation.^[10]^ Total synthesis via multiple steps with relatively low yields was only very recently developed for darobactin A (DA) and requires the usage of toxic chemicals.[^17^, ^18^] Excitingly, recently published heterologous expression platforms of the darobactin biosynthetic gene cluster (BGC) offer reasonable production rates in *E. coli* strains in low cost standard cultivation media.[^10^, ^16^, ^19^] The darobactin BGC consists of two genes essential for production. One encodes the precursor peptide DarA and the other encodes the radical *S*‐adenosylmethionine (rSAM) enzyme DarE, catalyzing the bicyclic crosslinking.^[20]^ Heterologous production rates of up to 25 mg L^−1^ in the case of DA and 3 mg L^−1^ for the more active darobactin 9 (D9), generated via biosynthetic pathway engineering, open the door for efficient large‐scale production.^[19]^ While the cryo‐EM structure of DA in complex with BamA already exists,^[12]^ no structure–activity relationship (SAR) rationale has been reported.

Herein, we describe a SAR study inspired by the structure elucidation of the BamABCDE (BAM)‐D9 complex via cryo‐EM, which aided the design of new darobactin core peptide sequences in vitro. A new, fast, and less expensive core peptide modification strategy, based on the overlap‐extension polymerase chain reaction (OE‐PCR) method facilitated the generation of fifteen new *darA* gene variants, including multiple halogenated tryptophans achieved through feeding experimentation, leading to promising derivatives with improved bioactivity and altered core sequences compared to DA, while conserving both tryptophan moieties critical for bicyclization.^[20]^ Most notably, darobactin 22 (D22) shows significantly improved minimal inhibitory concentration (MIC) values against clinically relevant pathogens, such as clinical isolates of carbapenem‐resistant *A. baumannii* (up to 32‐fold) and *P. aeruginosa* (up to 4‐fold). Single‐particle cryo‐EM structure elucidation explained the binding behavior of the most active new derivative compared to established DA and D9.

## Results and Discussion

**Figure 1:**
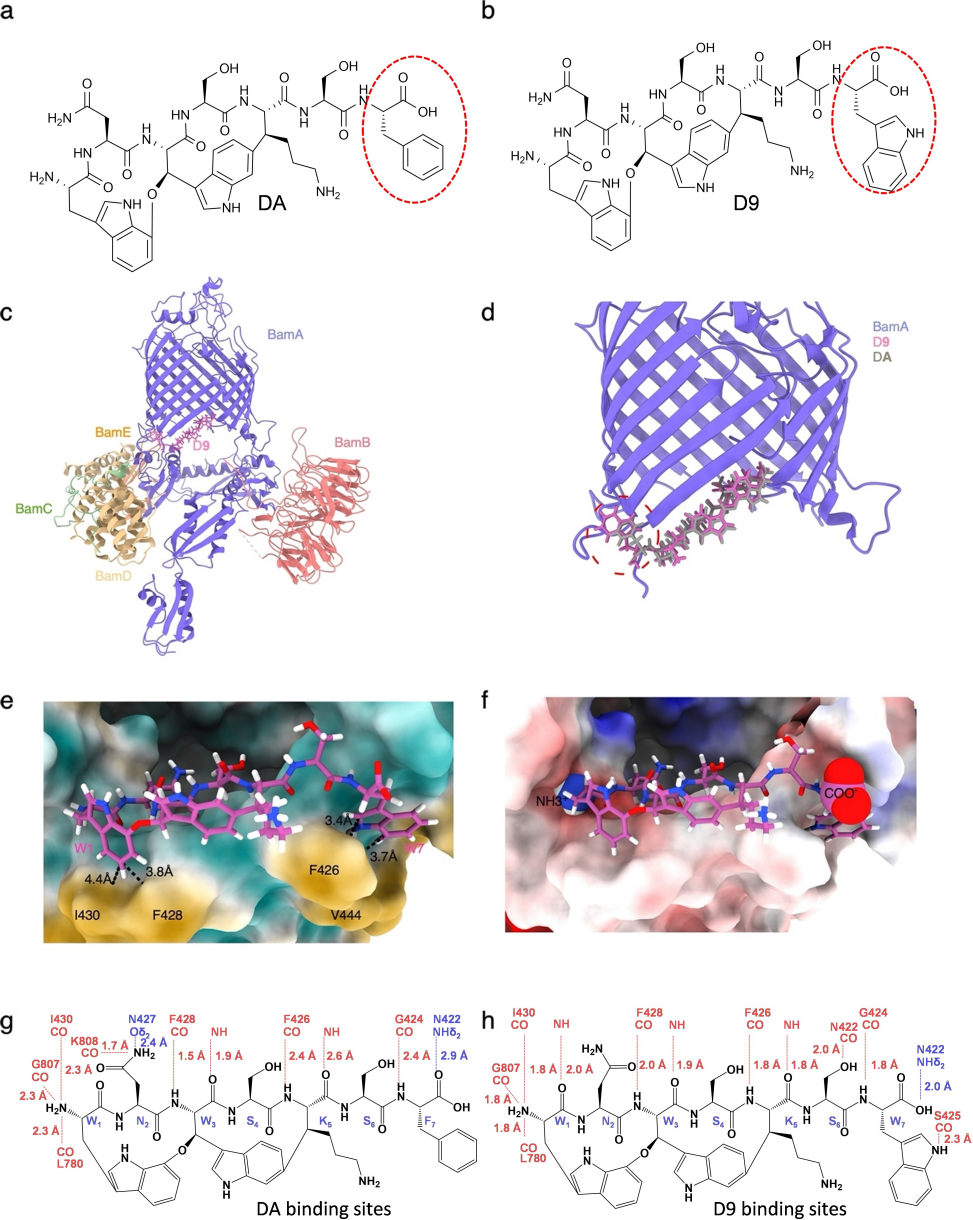
a–b) Structure formulae of DA and D9. The phenylalanine at the DA position 7 (F7) and the D9 tryptophan are indicated with a red dashed circle. c) Three‐dimensional structure of the D9 bound BAM as resolved by cryo‐EM. d) Magnified view of D9 bound to the surface of BamA and compared with DA. e–f) Magnified view of BAM‐D9 showing the local (hydrophobic) environment (green=hydrophilic; orange=hydrophobic) in the binding pocket and the positive (blue) and negative (red) electrochemical potential in the BAM‐D9 interaction area. g–h) 2D scheme of BamA‐darobactin hydrogen bond interactions. Canonical β‐strand hydrogen bonds (red), side chain interactions (blue), and distance variations when comparing DA^[12]^ (g) and D9 (h) were exhibited.

Five natural darobactins (DA to DE) were previously described.[^10^, ^19^, ^21^] In a recent work by our group, the non‐natural derivatives D1 to D21 were produced, including a derivative in which the terminal phenylalanine (DA, *W_1_N_2_W_3_S_4_K_5_S_6_F_7_*) is exchanged with a tryptophan (D9, *W_1_N_2_W_3_S_4_K_5_S_6_W_7_*) as seen in Figure 1 a‐b. The latter increases the antibacterial activity 2‐ to 8‐fold against a large panel of pathogens.^[19]^ The superior antibacterial activity of modified D9 compared to the native DA was the result of engineering the darobactin biosynthetic pathway and created a basis for new targeted structural improvements.^[19]^ To determine if D9 shares the same binding site as DA, we first resolved the cryo‐EM structure of the *E. coli* BAM complex bound to D9. While the overall average resolution was determined to be 3.4 Å, the local resolution the D9 estimated using Cryosparc revealed a resolution of 3.0 Å around binding region (Figure 1 c, d; Figure S1 a–e; Table S7). Binding to the β‐barrel of the *E. coli* BAM complex was deciphered revealing high similarities to the binding of DA with the BAM complex shown in Figure 1 d in addition to the overlaid electron density of both compounds (Figure S2).^[15]^ D9 also binds to the β1 of the lateral gate of BamA with multiple hydrogen bonds in antiparallel β‐sheet conformation, mimicking a β‐sheet extension thereby blocking the functional region of BamA (Figure 1 c, d; Figure S1 f, g).^[12]^ Moreover, the N‐ and C‐terminal ends of D9 are also tightly anchored to BamA via electrostatic and hydrophobic interactions (Figure 1 e, f). Furthermore, the distances for the establishment of hydrogen bonds of D9 to atoms of the binding site are well within the expected values and suggest that altered core peptides result in highly active darobactin derivatives such as the previously published darobactins DB, D4, D9, D11, D14, and D16.^[19]^ Of note, D9 is more tightly bound to BamA, as shown by comparison with native DA (Figure 1 d, g, h), which could be explained by additional side chain and backbone interactions of l‐tryptophan at position 1 with NH of I430, at position 6 with the carbonyl group of N422, and at position 7 with the carbonyl group of S425 (Figure 1 g, h). The novel hydrogen bond interactions and accompanied changes in D9 orientation are at the expense of l‐asparagine hydrogen bond interaction on position 2 with the carbamoyl oxygen of the side chain (Oδ_2_) of N427. Remarkably, the C‐terminal carboxyl group of the terminal tryptophan forms a shorter hydrogen bond with NHδ_2_ of N422 in contrast to l‐phenylalanine (Figure 1 g, h). Furthermore, the aromatic group of the C‐terminal tryptophan of D9 is significantly extended into the hydrophobic pocket formed by residues F426 and V444, potentially generating strong hydrophobic interactions (Figure 1 e). As a consequence, this might improve the ability to seal the lateral gate compared to native DA. Taken together, the additional interaction sites of D9 result in tighter binding,^[12]^ providing (1) structural evidence for the basis of 8‐fold enhanced activity of D9 over DA and (2) proof of concept that additional chemical alterations on DA could further improve binding as well as biological activity.

For the design of new non‐native darobactin derivatives, we considered currently available data, including relevant interactions of D9 (*WNWSKSW*) (Figure 1) and DA (*WNWSKSF*) with the BAM complex and the bioactivity ranking of darobactin‐containing extracts (DA to DE and D1 to D21), as well as the pure DA, DB and D9.[^10^, ^19^, ^21^] To ensure proper darobactin cyclization and ligand‐receptor interaction (Figure 1 g, h), positions 1, 2 and 3 of darobactin remained unaltered. In particular, manipulations to amino acid position 2 were avoided as this has previously resulted in reduced production rates and decreased antibacterial activity.[^19^, ^21^] Consequently, the binding behavior of DA and D9 on *E. coli* BAM complex revealed positions 4, 6 and 7 as the most flexible positions for core sequence engineering, without deteriorating target binding (Figure 1 g, h). Therefore, we designed fifteen new derivatives with alterations in these positions (Table 1): The increased interaction of the C‐terminal tryptophan at position 7 compared to smaller phenylalanine motivated the design of D26 to D28, which carry terminal l‐histidines. The expectation was that the aromatic imidazole ring of histidine could form strong interactions with the adjacent amino acids of BamA, similar to the aromatic rings of l‐phenylalanine or l‐tryptophan (Figure 1 a, b).

**Table 1:**
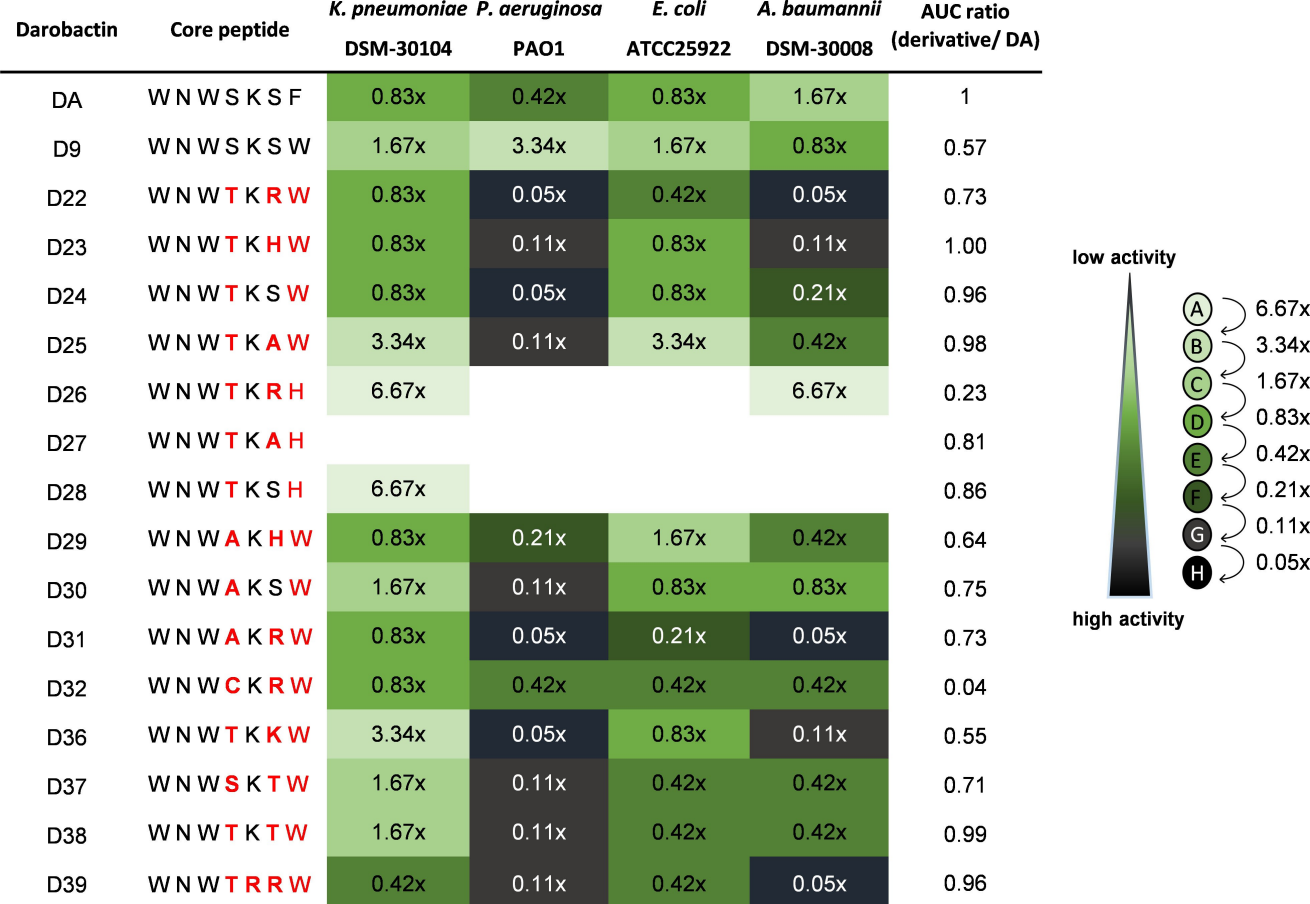
Darobactins with modified core peptide sequences and activity screen against *K. pneumoniae* DSM‐30104, *P. aeruginosa* PAO1, *E. coli* ATCC 25922 and *A. baumannii* DSM‐30008.Changes in the core peptide sequence to the native DA sequence are shown in red. Relative production titers of D9 and of its derivatives with respect to DA based on crude extracts were calculated by comparing area under the curve (AUC) ratios of new derivatives relative to DA. The antimicrobial activity of each derivative‐containing crude extract against tested pathogens is highlighted as a color code (bright green to dark green), depending on the highest dilution factor of standardized extract with respect to the assay volume [two‐fold serial dilution from 1 : 15 (A) (concentration factor: 6.67x) to 1 : 3,840 (H) (concentration factor: 0.05x)] in which full growth inhibition was detected (compare to Groß et al.^[19]^). UHPLC‐HRMS chromatograms and MS2 fragmentation pattern of produced darobactin derivatives are shown in Figure S5–32 and Figure S35–36, respectively. Derivatives D33 (*WNWCKSW*), D34 (*WNWCKAW*) and D35 (*WQWTKAW*) were designed but not generated due to purification issues of cysteine derivative D32 (D33, D34) or the expected low activity (D35).^[21]^

The comparison of the three derivatives, together with native DB (*WNWTKRF*) and published non‐natural derivative D16 (*WNWSKAF*)^[19]^ enabled us to study the effect of changing l‐serine at position 6 to either l‐arginine or apolar l‐alanine. Additionally, increased activity for DB and D4 (*WNWTKSF*) compared to DA motivated the exchange of l‐serine to l‐threonine at position 4.[^19^, ^21^] The impressive improvement in activity due to replacing the terminal l‐phenylalanine with an l‐tryptophan led to the design of more derivatives derived from D9 with varying amino acids at positions 4 and 6. Consequently, derivatives D22 to D25 and D36 to D38 carrying l‐arginine, l‐histidine, l‐serine, l‐alanine, l‐lysine or l‐threonine on position 6 and lthreonine on position 4, as present in the more active DB,^[21]^ were altered to evaluate the influence of each position. Replacing l‐serine at position 4 with l‐alanine, appeared to have less impact on activity, as observed in D14 (*WNWAKSF*),^[19]^ possibly due to the lack of direct side chain interaction of position 4 (Figure 1 g, h). To further study the influence of position 4, D29 to D32 were designed, which correspond to related D22 to D24 and D6.

**Figure 2:**
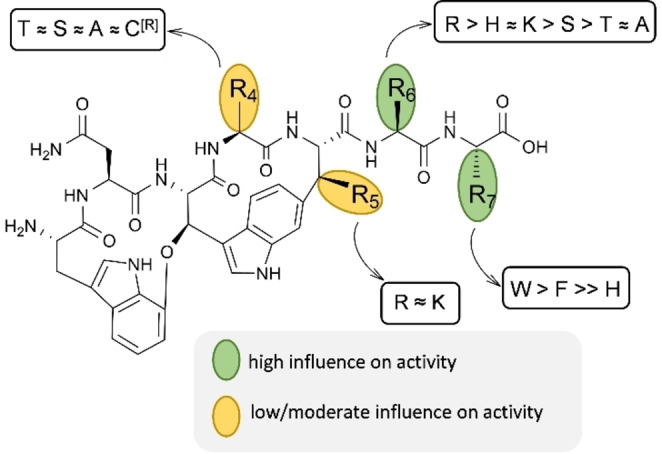
Summary of amino acid modifications and their influence on antibacterial activity. SAR study results in altered amino acids on position 4, 5, 6 and 7 of the heptapeptide. Tested amino acids are shown as one letter abbreviations (whereas C^[R]^ indicates the l‐cysteine adduct) and ordered depending on their antibacterial bioactivity against *A. baumannii* DSM‐30008, based on a MIC assay using crude extracts (Table 1). “≈” comparable activity; “>” or “»” higher activity.

Notably, change of the amino acid in position 5 from l‐lysine to l‐arginine resulted in lower production yields and reduced activity, as shown by the extract activity in our previous publication and by analyzing pure DD (*WNWTRSF*) by Böhringer et al.[^19^, ^21^] However, this data is contradictory to the darobactin binding mode determined in our cryo‐EM experiments.^[21]^ The backbone of l‐lysine at position 5 is only involved in interaction with the BamA β1‐strand on F426 with its carbonyl‐ and on A427 by the nitrogen of the amide group (Figure 1 g, h).^[12]^ The l‐lysine side chain with its terminal amino group is not involved in darobactin‐BamA interaction, but is oriented into the exterior region of the β‐barrel (Figure 1 d–f; Figure S1 f). Kaur et al. found that the positive charge of lysine side chain interacts with phosphate moieties of cardiolipin and 1‐palmitoyl‐2‐oleoyl‐*sn*‐glycero‐3‐phosphoglyc‐erol,^[12]^ which should also hold true for arginine. Accordingly, we were interested in the design of D39 with l‐threonine at position 4, l‐arginine at position 5 and 6 and l‐tryptophan at position 7 (*WNWTRRW*). The designed darobactin core sequences encoding for D22 to D32 and D36 to D39 (Figure S3) were generated by introducing point mutations via OE‐PCR in pNOSO‐*darABCDE*‐9 (DSM 33802). A detailed explanation can be found in the methods (Figure S4; Table S2). Subsequent mass spectrometry (MS) analysis (Table S3; Figure S5–36) and bioactivity ranking of the crude extracts for prioritization of the most active derivatives were performed as described in our previous publication, which led to the discovery of D9.^[19]^ Different molecular ions observable in electrospray ionization (ESI) MS were used in combined extracted ion chromatograms (EIC) to obtain comparable data for the area under the curve (AUC), since introduction of basic amino acids shifted the most abundant ion species from the doubly charged to the triply charged species (Table 1). The activity screening clearly showed an improvement of antibacterial activity of all newly generated derivatives in comparison to DA and at least comparable activity to D9, except for derivatives exhibiting the terminal l‐histidine (D26 to D28). The exchange to l‐histidine seems to abolish potency, which could be due to the slightly positive electro‐chemical potential in the C‐terminal binding area of BamA (Figure 1 f) and, in turn, may prevent histidine from binding into the hydrophobic binding pocket. Nevertheless, the activity of every other tested derivative with a terminal l‐tryptophan is auspicious. Interestingly, activity testing indicated a strong effect elicited by changes at position 6, which is detectable when comparing activity data of D22 to D25 and D36. This effect was most pronounced in *A.baumannii* DSM‐30008 and less obvious in *P. aeruginosa* due to generally higher potency. Overall, the SAR due to changes at position 6 in *E. coli* and *K. pneumoniae* was less obvious. Notably, the SAR of D37 and D38 suggests that *A. baumannii* is more sensitive to the modification of position 6, particularly when the residue is changed from the positively charged side chain of l‐lysine or l‐arginine. This could hint at more pathogen‐specific interactions or other factors affecting the antibacterial potency of darobactins. Based on the direct comparison of D22 with D31 and D32, it can be concluded that changes on position 4 from l‐threonine to l‐alanine or extended l‐cysteine residues as described previously,^[19]^ have only minor effects on activity. NMR structure elucidation determined that the l‐cysteine in these cases is connected via a disulfide bond to another l‐cysteine and lactic acid (Figure S76–85, Table S13, S14). Interestingly, D39, with l‐arginine instead of l‐lysine as a ring‐forming amino acid exhibited stronger antibacterial activity. This finding is contradictory to the 16‐fold decrease in activity when comparing DA and DD,[^19^, ^21^] but in line with predictions based on the expected binding mode (Figure 1 d, h), suggesting a different reason for the activity decrease of DD.^[21]^ Our findings regarding SAR of crude extracts are summarized in Figure 2. While crude extract data should be taken with caution,^[19]^ MIC assays done with purified darobactin derivatives were completely in line with extract data (see below).

Another approach to darobactin modification is the altering of tryptophans e.g., through halogenation. The tryptophan indole ring positioning is integral for darobactin blocking of the β‐barrel lateral gate.^[12]^ We considered the possibility that halogenation of these essential elements could significantly change bioactivity. Five new halogenated derivatives of D9 were indeed produced through feeding (see Supporting Information) of halogenated l‐tryptophans, two of them harboring 5‐chloro‐ and three 6‐fluoro‐l‐tryptophan (Figure S37–40). However, the initial yields prohibited isolation of all derivatives. The highest yield derivative with 6‐fluoro‐l‐tryptophan at position 3 of the core‐peptide could be purified and its structure was confirmed via NMR spectroscopy (Figure S86–90; Table S15). The corresponding activity analysis of purified, single fluorinated derivatives D9–6F1 and D9–6F7 showed comparable activity to pure D9 with slightly improved activity against *E. coli* and *K.pneumoniae* and slightly diminished activity against *P.aeruginosa*. (Figure S41). In general, modifications to darobactin amino acids, such as halogenation, expand the structural diversity spectrum of new darobactins, potentially influencing binding behavior and pharmacokinetic characteristics as known for fluoroquinolones.^[22]^ This assessment, along with developing a workflow to improve the yield of halogenated darobactins, will be part of a separate study. Thus, for the current phase of this project, we chose to focus on purification and characterization of new non‐halogenated derivatives with promising crude extract activity (Table 1, Figure 2).

To verify the promising activity data of crude extracts, certain derivatives with increased activity were selected for large‐scale production and isolation. During the production optimization of D22, the commercially available *E. coli* BL21‐Gold (DE3), engineered for reduced leaky expression (Agilent Technologies, California, USA), was evaluated and found to be favorable for production control of darobactins, due to their autotoxicity against the Gram‐negative host. While there was no significant difference in D22 production titer when comparing *E. coli* BL21 (DE3) (10.5 (±1.0) mg L^−1^) to *E. coli* BL21‐Gold (DE3) (9.0 (±1.2) mg L^−1^), we opted to use *E. coli* BL21‐Gold (DE3). This allowed for a more controlled fermentation, and thus better reproducibility in contrast to significantly increased leaky expression in uninduced *E. coli* BL21 (DE3), as shown in Groß et al.^[19]^ An optimized purification procedure utilized weak cation‐exchange resin Dowex MAC‐3 and ammonia elution instead of XAD16‐N as previously published.^[19]^ With this modified workflow, following an initial C18 flash chromatography step, we were able to reduce the purification effort of the darobactins to a single preparative C18 step that resulted in an increase in D22 yield from 0.8 mg L^−1^ culture to 3.0 to 3.7 mg mL^−1^ (3.8 to 4.6‐fold). The detailed purification procedure can be found in the Supporting Information.

The purified darobactins D22, D23, D31, D32, D36, D37 and D38 were structurally verified via NMR (Figure S46–75, Table S7–12) and tested against a panel of different Gram‐negative pathogens including *A.baumannii, E.coli, K.pneumoniae*, and *P.aeruginosa*. The activity was compared to previously published DA activity data^[10]^ and the crude extract data shown above (Table 1). Darobactins are not active against pathogens with phylogenetically distant BamA gene loci, like *Proteus mirabilis* or *Serratia marcescens* (Table 2; Figure S42), as expected.^[12]^ However, many new derivatives depict promising antibacterial activity against the majority of tested pathogens (Table 2) and are in line with the previously generated MIC data of crude extracts (Table 1). Compounds D22 and D31 stand out by demonstrating up to an eight‐fold increased activity against the panel of pathogens compared to native DA. Additionally, there was up to a two‐fold improved activity compared to D9, most interestingly against *Acinetobacter* strains.^[19]^ Moreover, direct comparison of D22, D31 and D32 emphasizes the minor influence of manipulations made in the core sequence corresponding to amino acid position 4. The substantial influence of changes to position 6 is reflected by comparing the differing activities of D22 and D38 in addition to the activities of D9^[19]^ and D37. Changing l‐serine to l‐threonine or l‐arginine to l‐threonine decreased bactericidal activity significantly (Table 2), whereas a basic side chain can enhance the activity demonstrated by comparison of DA with D36. However, these effects are generally species‐specific, most notably for the *K. pneumoniae* strains. Furthermore, no cytotoxicity was observed against the human cell line HepG2 as published for DA and D9.[^10^, ^19^]

**Table 2:**
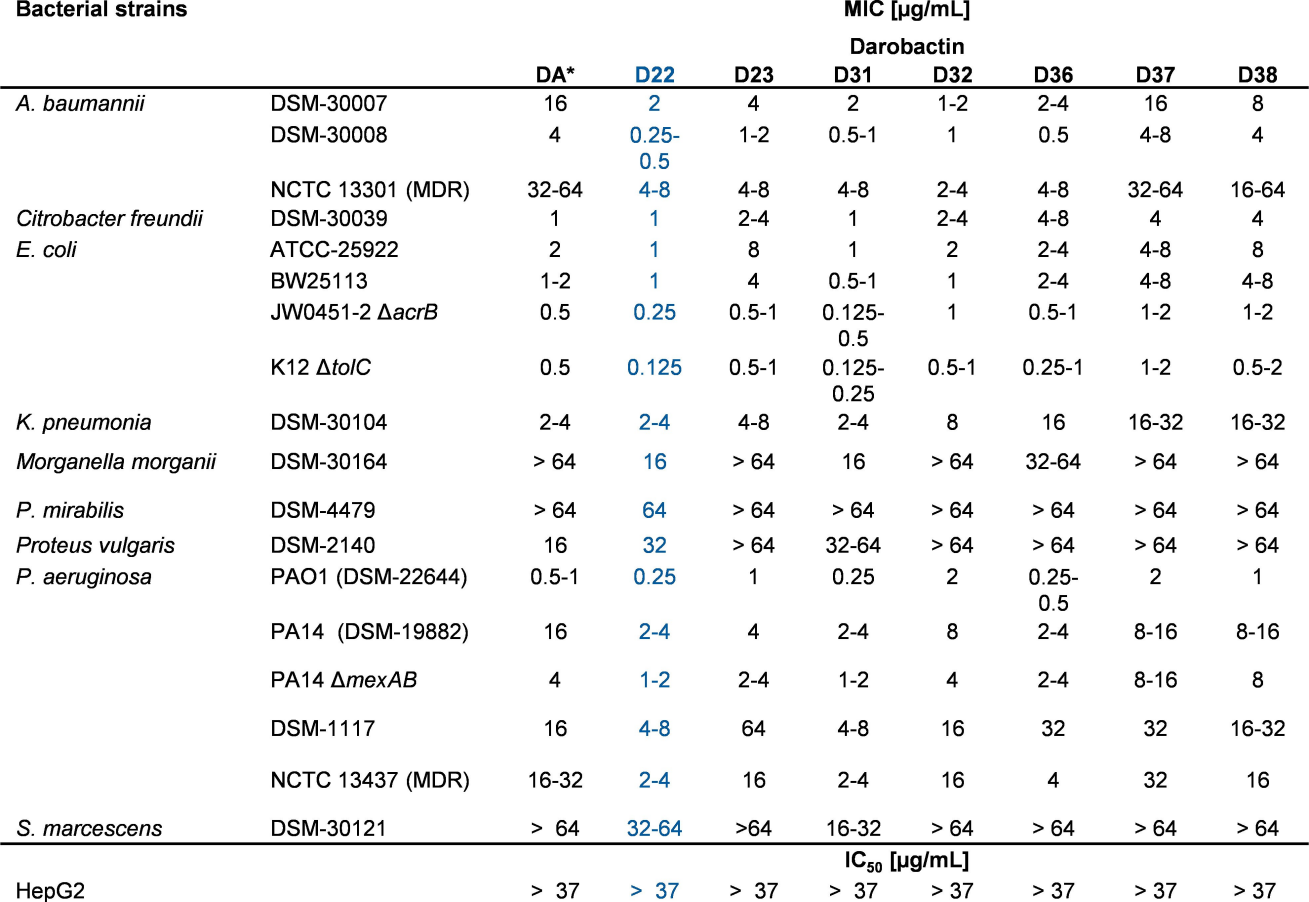
Antibacterial activity of pure darobactins: Compounds D22, D23, D31, D32, D36, D37 and D38 were tested in a MIC assay (in μg mL^−1^) against clinically relevant Gram‐negative pathogens. Superior activity of the new designed derivatives was analyzed by comparing them to DA data^[19]^ as control.

**Figure 3:**
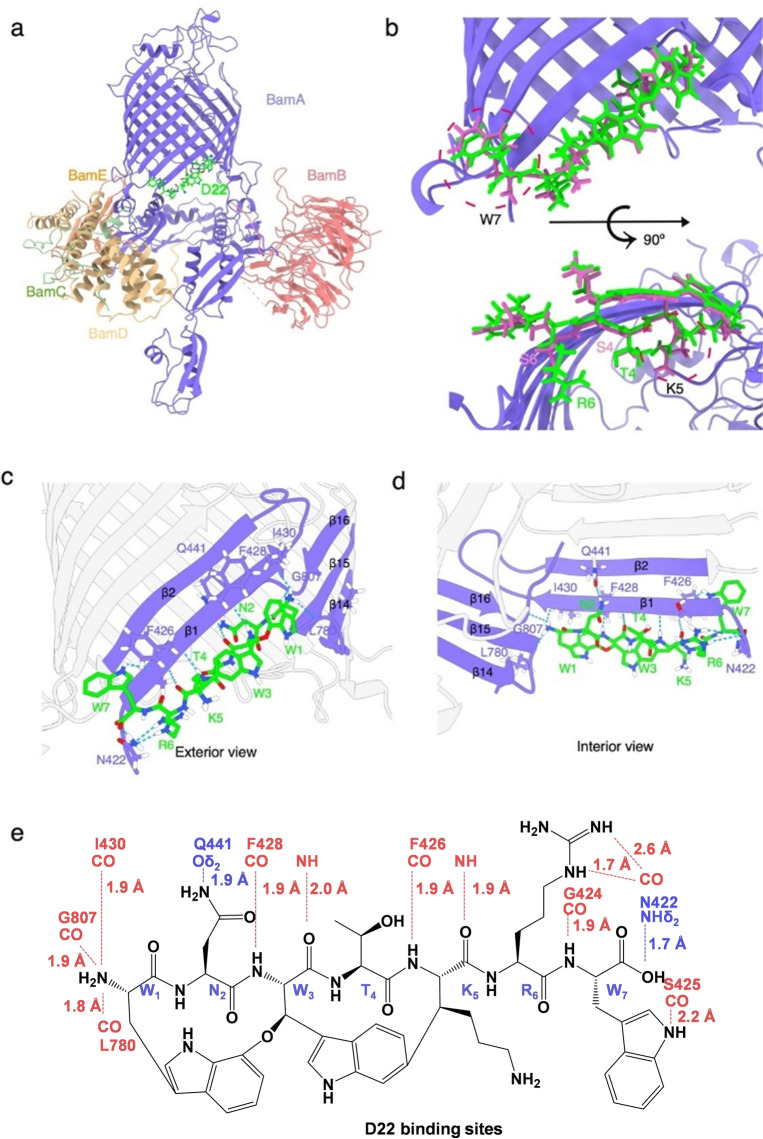
a) Cryo‐EM structure of the D22 bound to the BAM complex. b) Magnified view of D22 (lime) bound to the surface of BamA compared to D9 (pink). The positions with variable conformations were indicated in red dashed circle. The variable amino acids at the same position were also indicated with residue name in different colors. c–d) Magnified view of BamA‐D22 from exterior and interior views. Amino acids involved in hydrogen bond interaction are displayed as sticks and indicated by one letter abbreviation. Green dashes highlight hydrogen bond pairs. e) 2D scheme of BamA‐D22 hydrogen bond interactions. Canonical ß‐strand hydrogen bonds are shown in red and side chain interactions in blue color.

To investigate the SAR of D22, which exhibited the best activity profile of all known darobactins,[^10^, ^19^, ^21^] a cryo‐EM co‐structure determination of D22 bound BAM complex was initiated to study the potentially changed mode of binding (MoB). The structural basis of superior activity of D22 was analyzed by single‐particle co‐structure elucidation via cryo‐EM of *E. coli* BAM‐D22 complex (Figure 3) and compared to the co‐structure of BAM‐D9 (Figure 1). The cryo‐EM structure of the *E. coli* BAM complex was resolved with an overall resolution of 3.0 Å (Figure 3 a; Figure S43; Table S6). The MoB of D22 is comparable to D9, but with N_2_ and W_7_ conformational changes, which is probably caused by substitution of T_4_ and R_6_, respectively (Figure 3 b). The C‐terminal tryptophan is pushed deeper into the hydrophobic binding pocket and forms a closer hydrogen bond with the nitrogen of the indole ring and S425, compared to BamA‐D9 (Figure 3 b–e; Figure 1 h). N422 of BamA still pairs with the C‐terminal D22 carbonyl group via NHδ_2_ side chain interaction. Moreover, N422 forms one additional bond with the guanidine group of R_6_, probably causing the tighter seal of C‐terminal tryptophan into the binding pocket. A side chain interaction of Q441 from β‐strand 2 is formed due to its side chain remodeling (Figure 3 e).

These conformational changes are likely to be responsible for increased binding affinity and consequently improved bioactivity at least in *A. baumannii* strains. Furthermore, the significantly changed interaction and orientation at the C‐terminal end is not at the expense of one of three N‐terminal hydrogen bond interactions, maintaining proper closing of the lateral gate of BAM (Figure 3 c, d). The fact that T_4_ is not involved in direct binding with BamA β‐barrels makes this residue attractive for evaluating other amino acids or other moieties for improving pharmacological properties. Taken together, the different darobactin‐BamA binding interactions of DA, D9, and D22 likely reveal the structural basis for enhanced activity of engineered darobactins, which are more closely paired with BamA to mimic the β‐sheet prolongation and to lock the BAM complex in the inactive state by sealing the lateral gate tightly (Figure 1, 3).^[14]^ These structure–activity based findings are reinforced by the results of analyzing the different binding kinetics for DA and D9 versus D22 on purified *E. coli* BamA revealing *K*_D_ values of 0.278 μM, 0.3 μM and 0.159 μM, respectively (Figure S44). Nevertheless, more extensive activity profiling assays must be performed to generate a more valid correlation between the increased activity of D22 and the structural changes compared to DA and D9.

As D22 distinguishes itself by its superior activity, especially against tested *Acinetobacter* strains (Table 2), this derivative was tested in a MIC assay using hard‐to‐treat clinical isolates of carbapenem‐resistant *Acinetobacter baumannii* (CRAB), where most of the commonly used antibiotics are not active (Table S4). Here, the activities of DA, D9 and D22 were compared to colistin (COL), the antibiotic of last resort to treat infections caused by CRAB, which can cause nephrotoxicity.^[23]^

Intriguingly, D22 was found to be highly active against all nine tested clinical CRAB strains (MICs 0.06 to 0.5 μg mL^−1^) (Table 3), displaying significantly higher potency than the previous frontrunner D9 (and DA) and nearly equipotent antibacterial activity with colistin plus showing much better activity than other clinically used antibiotics (Table S4).^[24]^ In particular, the in vitro potency of D22 against CRAB was at least one order of magnitude better than for D9. This marked improvement is even more impressive as D9 displays already four‐fold improved activity compared to native DA.

**Table 3:**
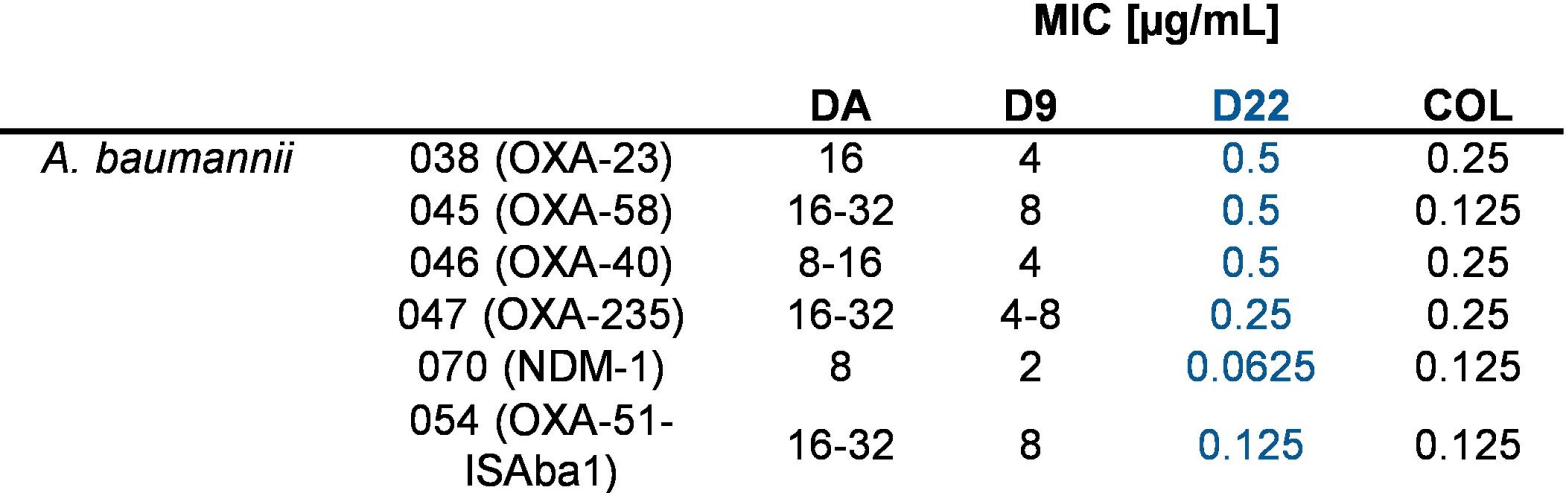
Bioactivity of DA, D9 and D22 against clinical CRAB isolates compared with colistin (COL). MIC in μg mL^−1^.

**Figure 4:**
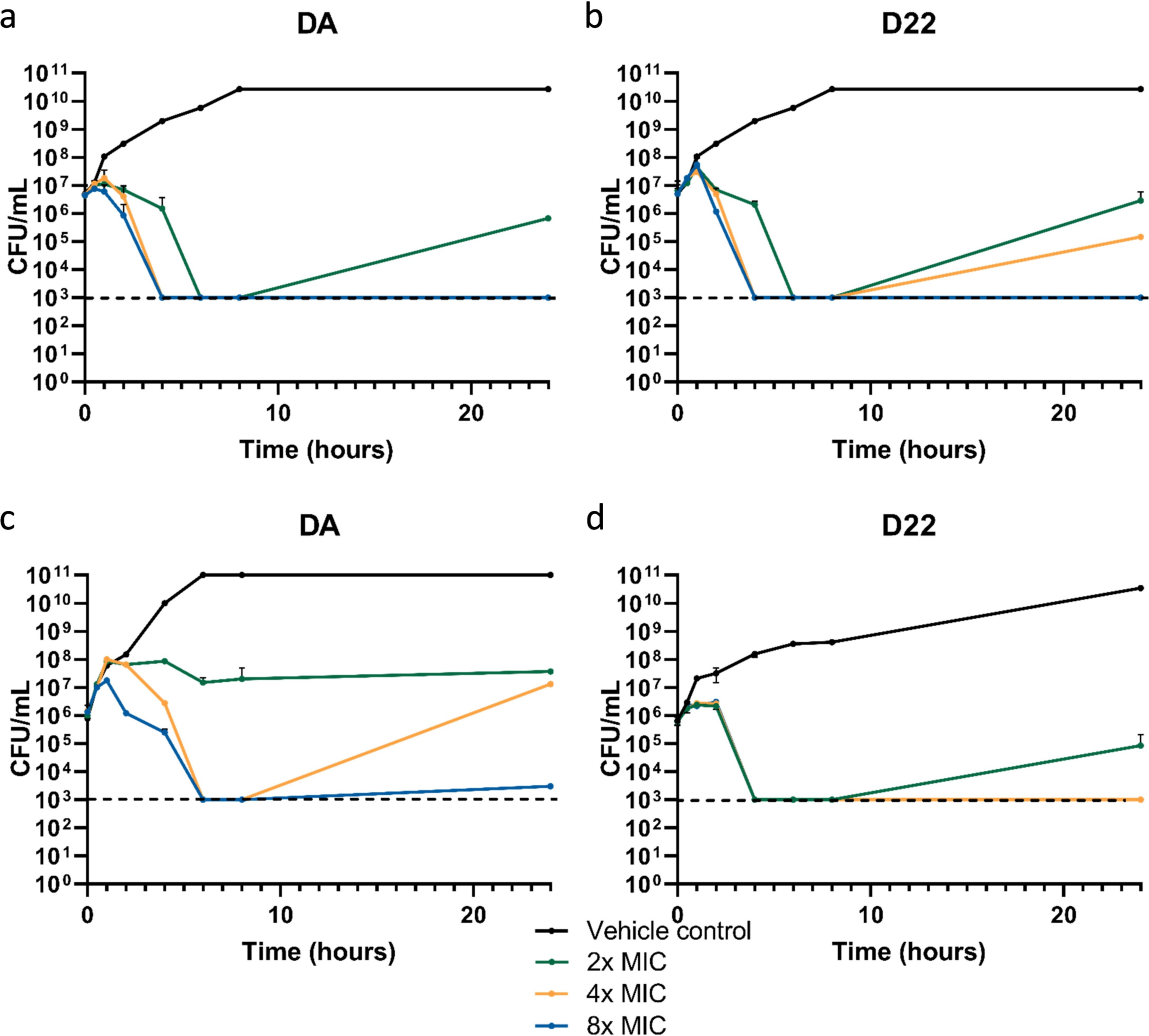
Time‐kill curves (TKCs) support bactericidal MoA of darobactins. a–b) TKC of *E. coli* MG1655 with DA and D22 using 2x, 4x and 8x MIC. c–d) TKC of *A. baumannii* NCTC13301 with DA and D22 using 2x, 4x and 8x MIC. Data are shown as mean±SD (*n*=3).

In addition, time‐kill kinetics of DA and D22 at 2x, 4x and 8x MIC in *E. coli* MG1655 (Figure 4 a, b) and in *A*. *baumannii* NCTC13301 (Figure 4 c, d) were assessed over a period of 24 hours. D22 shows rapid bacterial killing of *E*. *coli* MG1655 at 4x and 8x MIC after an early static phase of up to 2 hours followed by bactericidal activity with more than 3‐log killing exhibited in the reduced colony‐forming units (CFU mL^−1^) within 2 to 4 h. The observed initial bacteriostatic effect can possibly be explained by Ritzmann et al., which demonstrated that binding of darobactin on the lateral gate leads to an increased stability by “freezing” BamA and consequently no immediate bactericidal effect.^[14]^ However, DA and D22 at 2x, 4x and 8x MIC acted bactericidal over the first 8h in time‐kill experiments using *E. coli* MG1655, and the extent of bactericidal activity was concentration‐dependent. Nevertheless, re‐growth of bacteria was observed at 2x and 4x MIC conditions after 24 h, while this was not the case at 8x MIC (MIC values reported for DA and D22 were 2 μg mL^−1^ and 1 μg mL^−1^, respectively). Imai et al. did not report re‐growth of *E. coli* after DA treatment, however, the timeframe in their reported experiment was limited to 8h with 16x MIC, making direct comparison challenging.^[10]^ They also determined a minimum bactericidal concentration (MBC) of 8 μg mL^−1^ in *E. coli*, which is fully in line with our findings for DA and D22 having MBCs of 16 and 8 μg mL^−1^, respectively.^[10]^ Encouragingly, upon repeating the time‐kill experiment using high inoculum conditions (≈5×10^8^ CFU mL^−1^) we were able to show that surviving bacteria are likely persisters^[25]^ and did not gain resistance, as representative colonies from this experiment showed no MIC shifts (Table S5). In a comparison of time‐kill kinetics of both darobactins in *A. baumannii* NCTC13301, D22 shows its clear superiority over DA (Figure 4 c, d). For the latter, we determined a lasting bacteriostatic effect at 2x MIC, and re‐growth was observed at both initially bactericidal concentrations (4x and 8x MIC). In contrast, D22 showed bactericidal activity and concentrations as low as 4x MIC and fully prevented re‐growth at 24 h post‐treatment (MBC of 32 μg mL^−1^).

Considering that *A.baumannii* easily acquires resistance, making CRAB a WHO priority one pathogen for antibiotic development,[^8^, ^26^] the results of D22 are highly promising. It displays equipotent in vitro activity with colistin against clinical CRAB isolates, while colistin exhibits severe side effects. D22 did, however, not show toxic effects tested in vitro with HepG2 cells (Table 2) and in vivo using the highly sensitive zebrafish larvae model up to 500 μg mL^−1^ (Figure S45), which emphasizes the potential for application and encourages further development towards proof of concept in humans.

## Conclusion

Preceding darobactin SAR studies focused on native darobactins[^16^, ^21^] or were based upon observations from them.^[19]^ Elucidation of the MoB of DA and D9 to BamA, combined with structural data of recently published darobactins,[^12^, ^14^, ^19^, ^21^] enabled a more guided approach, in which we constructed twenty new derivatives, including twelve derivatives with terminal l‐tryptophan exhibiting similar or higher activity than DA (Figure S3).

According to our findings, structural changes at position 4 appears to have only a minor influence, while positions 6 and 7, necessary for interactions with β‐hairpins of BamA, seem to have a major influence on activity and could be used as a potential starting point for improvement of pharmaceutical properties through structural changes. The most promising compound developed to date, based on our SAR study is the biosynthetically engineered new darobactin D22, which surpasses the antibacterial activity of all identified native darobactins. This is remarkable, as natural products are produced in co‐evolution over millions of years, resulting in structural diversity and target specificity for fungi, eukaryotes and bacteria.^[27]^ The engineered derivative D22 is highly active against a broad range of Gram‐negative bacterial pathogens demonstrating superiority even to the previous, biosynthetically engineered frontrunner D9, which already surpassed the activity of originally reported native DA.^[19]^ In particular, D22 displays bactericidal activity against hard‐to‐treat CRAB and is equipotent to the highly active, yet nephrotoxic, last resort antibiotic colistin. Furthermore, D22 was shown to be non‐toxic in initial assays in vitro (HepG2) and in vivo (zebrafish larvae). Further studies will explore the optimized effects of D22 regarding mechanistic, kinetic, and energetic properties, e.g. regarding the linker region and the eight β‐hairpins, as detailed by Ritzmann et al.^[14]^ for DA.

Future studies should also include a comparison to the structurally variable BAM complex of *A. baumannii* strains, of which a co‐structure with darobactins has not yet been solved. However, our activity and toxicity data of D22 are encouraging to advance into further preclinical studies. While currently established protocols for cultivation in *E. coli* are favorable for fast and robust generation of novel, even halogenated, darobactins in quantities suitable for research and development purposes,[^16^, ^19^] they are hard‐pressed to satisfy supply demands for such future studies, because of low self‐resistance of the Gram‐negative heterologous host *E. coli*. Changing the heterologous producer to a Gram‐positive strain such as *Bacillus subtilis*, which has been successfully applied in industrial fermentations, might enable higher darobactin titers. Alternatively, very recently published total syntheses reported for DA may be applicable but currently suffer from yield issues as well.[^17^, ^18^]

To summarize, our SAR approach presented structural knowledge about the MoB of the darobactins and resulted in novel derivatives with up to 32‐fold increased activity against CRAB, compared to the most potent known derivative D9.^[19]^ The new, distinguished D22 was found to be comparable to clinically used antibiotics in its activity against certain Gram‐negative pathogens. Consequently, our approach led to a new frontrunner molecule of darobactins showing more favorable antibacterial activity. Compound D22 is particularly active against critically prioritized Gram‐negative pathogens encouraging further preclinical studies with this highly promising scaffold to hopefully and eventually achieve proof of efficacy in humans for this exciting and promising molecule class.

## Conflict of interest

The authors declare no conflict of interest.

1

## Supporting information

As a service to our authors and readers, this journal provides supporting information supplied by the authors. Such materials are peer reviewed and may be re‐organized for online delivery, but are not copy‐edited or typeset. Technical support issues arising from supporting information (other than missing files) should be addressed to the authors.

Supporting Information

## Data Availability

The data that support the findings of this study are available from the corresponding author upon reasonable request.
