# Supplementary material for: Darobactins Exhibiting Superior Antibiotic Activity by Cryo‐EM Structure Guided Biosynthetic Engineering
Source: Angew Chem Int Ed Engl. 2022 Dec 7;62(2):e202214094. doi: 10.1002/anie.202214094 (PMC10107326; doi:10.1002/anie.202214094)
Supplement: Supplementary file 1 — Supporting Information [file ANIE-62-0-s001.pdf]

Angewandte Chemie
Eine Zeitschrift der Gesellschaft Deutscher Chemiker

Supporting Information

Darobactins Exhibiting Superior Antibiotic Activity by Cryo-EM Structure Guided Biosynthetic Engineering

C. E. Seyfert, C. Porten, B. Yuan, S. Deckarm, F. Panter, C. D. Bader, J. Coetzee, F. Deschner, K. H. M. E. Tehrani, P. G. Higgins, H. Seifert, T. C. Marlovits, J. Herrmann*, R. Müller**

Contents

Methods 6

1. *In silico* design and generation of novel darobactin derivatives by overlap-extension polymerase chain reaction 6

2. Overproduction of novel darobactin derivatives 6

3. Analysis and quantification of production titer 7

4. Overproduction of novel darobactin derivatives with induction 7

5. Overproduction of halogenated darobactins 7

6. Purification of novel darobactin derivatives 8

7. Determination of antibacterial activity 11

8. Characterization of darobactin 22 11

8.1 Maximum tolerated concentration (MTC) 11

8.2 Time-kill assay 11

9. NMR spectroscopy of D6, D22, D23, D31, D32, D36, D37 and D38 12

10. Protein expression and purification 12

11. Electron microscopy sample preparation and data processing, collecting and analyzing 13

12. Thermal titration calorimetry 13

13. List of bacterial strains 14

Supplementation figures and tables 17

Cryo-EM of BAM-D9 17

Chromatograms of darobactin extracts 23

Analyses of darobactin derivative MS2 spectra 28

Analyses of cysteine derivatives D6 and D32 MS and MS2 spectra 33

Analyses of halogenated D9 37

MS and MS2 spectra 37

BamA interaction site 41

Cryo-EM of BAM-D22 42

CRAB profiling 44

Antibiograms of clinical CRAB isolates 44

Cryo-EM data collection, refinement, and validation statistics. 46

NMR confirmation of darobactins 47

Supplementary tables

Table S 1: List of bacterial strains used or generated in this study. 14
Table S 2: Oligonucleotides used for overlap extension (OE) polymerase chain reaction I, II and III... 21
Table S 3: Calculated and observed masses of the darobactin derivatives. 22
Table S 4: Antibiograms of clinical CRAB isolates. 44
Table S 5: Activity assessment using 24 h population of TKC. 44
Table S 6: Cryo-EM data collection and refinement statistics and validation statistics of BAM-D9 and BAM-D22 data collection. 46
Table S 7: NMR spectroscopic data of D22. 47
Table S 8: NMR spectroscopic data of D23. 53
Table S 9: NMR spectroscopic data of D31. 59
Table S 10: NMR spectroscopic data of D36. 65
Table S 11: NMR spectroscopic data of D37. 71
Table S 12: NMR spectroscopic data of D38. 77
Table S 13: NMR spectroscopic data of D6[R]. 83
Table S 14: NMR spectroscopic data of D32[R]. 89
Table S 15: NMR spectroscopic data of D9-6F. 96

Supplementary figures

Figure S 1: Cryo-EM structure of BAM-D9 complex. 18
Figure S 2: EM density comparison of DA and D9. 18
Figure S 3 : Overview of all generated non-halogenated novel darobactin derivatives. 19
Figure S 4: Generation of new darobactin derivatives via overlap-extension PCR. 20
Figure S 5: Chromatogram of the *E. coli* BL21 (DE3) pNOSO-darABCDE-22 culture XAD16-N extract. 23
Figure S 6: Chromatogram of the *E. coli* BL21 (DE3) pNOSO-darABCDE-23 culture XAD16-N extract. 23
Figure S 7: Chromatogram of the *E. coli* BL21 (DE3) pNOSO-darABCDE-24 culture XAD16-N extract. 24
Figure S 8: Chromatogram of the *E. coli* BL21 (DE3) pNOSO-darABCDE-25 culture XAD16-N extract. 24
Figure S 9: Chromatogram of the *E. coli* BL21 (DE3) pNOSO-darABCDE-26 culture XAD16-N extract. 24
Figure S 10: Chromatogram of the *E. coli* BL21 (DE3) pNOSO-darABCDE-27 culture XAD16-N extract. 25
Figure S 11: Chromatogram of the *E. coli* BL21 (DE3) pNOSO-darABCDE-28 culture XAD16-N extract. 25
Figure S 12: Chromatogram of the *E. coli* BL21 (DE3) pNOSO-darABCDE-29 culture XAD16-N extract. 26
Figure S 13: Chromatogram of the *E. coli* BL21 (DE3) pNOSO-darABCDE-30 culture XAD16-N extract. 26
Figure S 14: Chromatogram of the *E. coli* BL21 (DE3) pNOSO-darABCDE-31 culture XAD16-N extract. 26
Figure S 15: Chromatogram of the *E. coli* BL21 (DE3) pNOSO-darABCDE-36 culture XAD16-N extract. 27
Figure S 16: Chromatogram of the *E. coli* BL21 (DE3) pNOSO-darABCDE-37 culture XAD16-N extract. 27
Figure S 17: Chromatogram of the *E. coli* BL21 (DE3) pNOSO-darABCDE-38 culture XAD16-N extract. 28
Figure S 18: Chromatogram of the *E. coli* BL21 (DE3) pNOSO-darABCDE-39 culture XAD16-N extract. 28
Figure S 19: MS2 spectrum of D22. 29

Figure S 20: MS2 spectrum of D23. 29
Figure S 21: MS2 spectrum of D24. 29
Figure S 22: MS2 spectrum of D25. 30
Figure S 23: MS2 spectrum of D26. 30
Figure S 24: MS2 spectrum of D27. 30
Figure S 25: MS2 spectrum of D28. 31
Figure S 26: MS2 spectrum of D29. 31
Figure S 27: MS2 spectrum of D30. 31
Figure S 28: MS2 spectrum of D31. 32
Figure S 29: MS2 spectrum of D36. 32
Figure S 30: MS2 spectrum of D37. 32
Figure S 31: MS2 spectrum of D38. 33
Figure S 32: MS2 spectrum of D39. 33
Figure S 33: Chromatogram of the *E. coli* BL21 (DE3) pNOSO-darABCDE-6 culture XAD16-N extract. 34
Figure S 34: MS2 spectrum of D6 before cleaving of with its most abundant protection group (a) and after reduction with β-mercaptoethanol (b). 35
Figure S 35: Chromatogram of the *E. coli* BL21 (DE3) pNOSO-darABCDE-32 culture XAD16-N extract. 36
Figure S 36: MS2 spectrum of D32 before cleaving of with its most abundant protection group (a) and after reduction with β-mercaptoethanol (b). 37
Figure S 37: Chromatogram of *E. coli* BL21 (DE3) pNOSO-darABCDE-9-6F culture XAD-16N extract. 37
Figure S 38: MS2 fragmentation pattern of D9-6F. 38
Figure S 39: Chromatogram of *E. coli* BL21 (DE3) pNOSO-darABCDE-9-5Cl culture XAD-16N extract. 39
Figure S 40: MS2 fragmentation pattern of D9-5Cl. 40
Figure S 41: Generation and activity of fluorinated D9 against Gram-negative bacteria. 41
Figure S 42: Alignment of BamA sequence variations. 41
Figure S 43: Cryo-EM structure of BAM-D22 complex. 42
Figure S 44: Thermal titration calorimetry assays. 43
Figure S 45: No *in vivo* cytotoxicity against zebrafish larvae. 45
Figure S 46: ^{1}H spectrum of D22 in ACN/D2O + 1% FA at 45 °C and 500 MHz. 49
Figure S 47: ^{13}C spectrum of D22 in ACN/D2O + 1% FA at 45 °C and 125 MHz. 49
Figure S 48: HSQC spectrum of D22 in ACN/D2O + 1% FA at 45 °C and 500/125 MHz. 50
Figure S 49: COSY spectrum of D22 in ACN/D2O + 1% FA at 45 °C and 500/125 MHz. 51
Figure S 50: HMBC spectrum of D22 in ACN/D2O + 1% FA at 45 °C and 500/125 MHz. 52
Figure S 51: ^{1}H spectrum of D23 in ACN/D2O + 1% FA at 45 °C and 500 MHz. 55
Figure S 52: ^{13}C spectrum of D23 in ACN/D2O + 1% FA at 45 °C and 125 MHz. 55
Figure S 53: HSQC spectrum of D23 in ACN/D2O + 1% FA at 45 °C and 500/125 MHz. 56
Figure S 54: COSY spectrum of D23 in ACN/D2O + 1% FA at 45 °C and 500/125 MHz. 57
Figure S 55: HMBC spectrum of D23 in ACN/D2O + 1% FA at 45 °C and 500/125 MHz. 58
Figure S 56: Water suppressed ^{1}H spectrum of D31 in ACN/D2O + 1% FA at 45 °C and 500 MHz. 61
Figure S 57: ^{13}C spectrum of D31 in ACN/D2O + 1% FA at 45 °C and 125 MHz. 61
Figure S 58: HSQC spectrum of D31 in ACN/D2O + 1% FA at 45 °C and 500/125 MHz. 62
Figure S 59: COSY spectrum of D31 in ACN/D2O + 1% FA at 45 °C and 500/125 MHz. 63
Figure S 60: HMBC spectrum of D31 in ACN/D2O + 1% FA at 45 °C and 500/125 MHz. 64
Figure S 61: Water suppressed ^{1}H spectrum of D36 in ACN/D2O + 1% FA at 45 °C and 500 MHz. 67
Figure S 62: ^{13}C spectrum of D36 in ACN/D2O + 1% FA at 45 °C and 125 MHz. 67
Figure S 63: HSQC spectrum of D36 in ACN/D2O + 1% FA at 45 °C and 500/125 MHz. 68
Figure S 64: COSY spectrum of D36 in ACN/D2O + 1% FA at 45 °C and 500/125 MHz. 69

Figure S 65: HMBC spectrum of D36 in ACN/D2O + 1% FA at 45 °C and 500/125 MHz. 70
Figure S 66: ^{1}H spectrum of D37 in ACN/D2O + 1% FA at 45 °C and 500 MHz. 73
Figure S 67: ^{13}C spectrum of D37 in ACN/D2O + 1% FA at 45 °C and 125 MHz. 73
Figure S 68: HSQC spectrum of D37 in ACN/D2O + 1% FA at 45 °C and 500/125 MHz. 74
Figure S 69: COSY spectrum of D37 in ACN/D2O + 1% FA at 45 °C and 500/125 MHz. 75
Figure S 70: HMBC spectrum of D37 in ACN/D2O + 1% FA at 45 °C and 500/125 MHz. 76
Figure S 71: Water-suppressed ^{1}H spectrum of D38 in ACN/D2O + 1% FA at 45 °C and 500 MHz. 79
Figure S 72: ^{13}C spectrum of D38 in ACN/D2O + 1% FA at 45 °C and 125 MHz. 79
Figure S 73: HSQC spectrum of D38 in ACN/D2O + 1% FA at 45 °C and 500/125 MHz. 80
Figure S 74: COSY spectrum of D38 in ACN/D2O + 1% FA at 45 °C and 500/125 MHz. 81
Figure S 75: HMBC spectrum of D38 in ACN/D2O + 1% FA at 45 °C and 500/125 MHz. 82
Figure S 76: ^{1}H spectrum of D6[R] in ACN/D2O + 1% FA at 45 °C and 500 MHz. 85
Figure S 77: ^{13}C spectrum of D6[R] in ACN/D2O + 1% FA at 45 °C and 125 MHz. 85
Figure S 78: HSQC spectrum of D6[R] in ACN/D2O + 1% FA at 45 °C and 500/125 MHz. 86
Figure S 79: COSY spectrum of D6[R] in ACN/D2O + 1% FA at 45 °C and 500/125 MHz. 87
Figure S 80: COSY spectrum of D6[R] in ACN/D2O + 1% FA at 45 °C and 500 MHz. 88
Figure S 81: ^{1}H spectrum of D32[R] in ACN/D2O + 1% FA at 35 °C and 500 MHz. 92
Figure S 82: ^{13}C spectrum of D32[R] in ACN/D2O + 1% FA at 35 °C and 125 MHz. 92
Figure S 83: HSQC spectrum of D32[R] in ACN/D2O + 1% FA at 35 °C and 500/125 MHz. 93
Figure S 84: COSY spectrum of D32[R] in ACN/D2O + 1% FA at 35 °C and 500 MHz. 94
Figure S 85: HMBC spectrum of D32[R] in ACN/D2O + 1% FA at 35 °C and 500/125 MHz. 95
Figure S 86: ^{1}H spectrum of D9-6F in ACN/D2O + 1% FA at 45 °C and 500 MHz. 98
Figure S 87: ^{13}C spectrum of D9-6F in ACN/D2O + 1% FA at 45 °C and 125 MHz. 98
Figure S 88: HSQC spectrum of D9-6F in ACN/D2O + 1% FA at 45 °C and 500/125 MHz. 99
Figure S 89: COSY spectrum of D9-6F in ACN/D2O + 1% FA at 45 °C and 500/125 MHz. 100
Figure S 90: HMBC spectrum of D9-6F in ACN/D2O + 1% FA at 45 °C and 500/125 MHz. 101

Methods

1. *In silico* design and generation of novel darobactin derivatives by overlap-extension polymerase chain reaction

Due to time and cost reduction, the generation of new derivatives was performed by insertion of point mutations into *darA* gene in three independent polymerase chain reactions (PCRs) (Figure S 4). In our approach, four different primers, named pr1dar_fw, pr2darXX_rv, pr3dartrp/his_fw and pr4dar_rv, were designed to generate one new darobactin derivative. Except of primer pr2darXX_rv (where XX stands for appropriate derivative, Table S 1), all primers can be re-used no matter which derivative is produced. Primer pr1dar_fw binds to a region located in the T7 lac promoter. Primer variants of pr2darXX_rv and primer pr3dartrp/his_fw are binding in the core region of *darA* and carry an overlapping region of 13 base pairs. Primer pr4dar_rv is binding in front of the termination region on the end of the intergenic region between *darA* and *darB* (Figure S 4). As depicted in Figure S 4, PCR reaction I with pr1dar_fw and pr2dar22_rv and PCR reaction II with pr3dartrp/his_fw and pr4dar_rv were performed in parallel, using pNOSO-darABCDE-9 (DSM 33802) as a template (Figure S 4). The two resulting PCR products, harboring homologous regions (highlighted in blue), were mixed in a 1 to 1 ratio and used as template for an overlap-extension PCR reaction (PCR reaction III). For PCR reaction III, primer pr1dar_fw and pr4dar_rv were re-used. The OE-PCR reaction consists of two amplification cycles performed at low and thirty-two at high annealing temperature. All used primers are listed in Table S 2. The DNA fragment amplified in PCR reaction III was gel-purified, restriction hydrolyzed and cloned into pNOSO-darABCDE-9 using standard restriction hydrolysis/DNA ligation cloning techniques. New plasmids with modified *darA* core DNA sequences were verified via Sanger sequencing at LGS biosynthetic. Figure S 3 shows the generated darobactin derivatives.

2. Overproduction of novel darobactin derivatives

Escherichia coli (*E. coli*) NEB10β strain was used for transformation of ligation mixtures with digested, modified *darA* gene fragment and pNOSO-darABCDE-9[1] backbone to generate new artificial darobactin BGC. Cultivation of bacterial cloning strain was performed in LB medium (10 g/L tryptone, 5 g/L NaCl, 5 g/L yeast extract, pH 7.6) at 30 °C. *E. coli* BL21 (DE3) or *E. coli* BL21-Gold (DE3) were taken as heterologous production hosts. Overnight culture with single colony of producer strain, previously transformed with generated expression vector (e.g. pNOSO-darABCDE-22 to 34 and 36 to 39), was incubated for 16 hours at 30 °C and 180 rpm on an orbital shaker (Orbitron/Multitron, Infors HT). 0.5 mL of seeded overnight culture was used for inoculation of 50 mL FM medium (12.54 g/L K_2HPO_4, 2.31 g/L KH_2PO_4, 5 g/L NaCl,

12 g/L yeast extract, 4 g/L D(+) glucose, 1 g/L NH_4Cl, 0.24 g/L $MgSO_4$, pH 7.0) including 1 mg/L sterile filtered vitamin B_{12}. 300 mL round bottom flasks were shaken at 30 °C for 3 days and 180 rpm on an orbital shaker (Orbitron/Multitron, Infors HT). Kanamycin (50 µg/mL; pNOSO-based expression constructs) was used as antibiotic selection marker.

3. Analysis and quantification of production titer

The 50 mL production broth was centrifuged at 7000 x g for 10 min at 4 °C and supernatant was collected. Production of darobactin in the supernatant was analyzed using an amaZon speed 3D ion trap MS system (Bruker Daltonics) with an Apollo II ESI source uHPLC-HRMS. The extracted ion chromatogram (EIC) was calculated using DataAnalysis software as previously described.[1] Darobactins in crude extract were measured via analytic ultra-high-performance liquid chromatography-high-resolution mass spectrometry (uHPLC-HRMS) using m/z values of $[M+2H]^{2+}$, $[M+3H]^{3+}$, $[M+3H-NH_3]^{3+}$ and MS2 fragmentation analysis of $[M+2H]^{2+}$ (Figure S 5 - Figure S 36) as calculated (Table S 3). Different ionization states were used in combined extracted ion chromatograms (EIC) to receive comparable data, because introduction of basic amino acids shifted the most abundant ion species from twofold charged $[M+2H]^{2+}$ to two threefold charged states ($[M+3H]^{3+}$ and $[M+3H-NH_3]^{3+}$). The novel cysteine derivative D32[R], carries the identical major protection group [R], as previously published D6[R], indicated by their identical +191.026 Da mass shifts and as confirmed via nuclear magnetic resonance (NMR) structure elucidation of pure D6[R] and D32[R] (Table S 3, Figure S 76 - Figure S 85). For determination of compound present in the extract relative to DA extract, MS peak area under the curve (AUC) of each crude extracts was computed by automated peak integration of combined EIC values using *Compass Data Analysis* version 5.3 (Bruker Daltonics) and divided through AUC of DA (Table 1 of the manuscript).

4. Overproduction of novel darobactin derivatives with induction

The established fermentation protocol of DA and 9**[1]** was taken to produce D22, D23, D31, D32, D36, D37, D38 in *E. coli* BL21 (DE3) after transformation of electro-competent *E. coli* BL21 (DE3) with pNOSO-darABCDE-22, -23, -31, -32, -36, -37 and -38. Using *E. coli* BL21-Gold (DE3) pNOSO-darABCDE-22 as a producer strain for D22 production, the *T7* RNA polymerase was induced by 0.4 mM isopropyl β-D-1-thiogalactopyranoside (IPTG) after an OD_{600} 0.8 to 1.0 was reached to allow a controlled gene expression of *darA* variant compared to *E. coli* BL21 (DE3).

5. Overproduction of halogenated darobactins

15 mL LB medium, including 30 µg/mL Kanamycin (Kan30), was inoculated with single colony of *E. coli* BL21 (DE3) harboring pNOSO-darABCDE-9. After overnight incubation at 30 °C, 180 rpm (Infors HT), 0.2 mL of seeded culture was used to inoculate 20 mL FM medium (1.254 % K_2HPO_4, 0.231 % KH_2PO_4, 0.4 % D(+)-glucose, 0.1 % NH_4Cl, 1.2 % yeast extract, 0.5 % NaCl

and 0.0492 % $MgSO_4$(*7H_2O); pH 7.1) including Kan30 and 1 mg/L Vitamin B_{12}. Cultures were supplemented with 6-fluoro-L-tryptophane to a final concentration of 2.1 mM in 20 mL of production culture after continually feeding over a period of 3 days. Feeding of 5-chloro-L-trytrophan, was tested in concentrations of 0.1 to 10 mM end concentration. The overproduction culture was grown under constant conditions at 30 °C and 180 rpm on an orbital shaker. For large scale production of fluorinated D9, volumes were up-scaled to 4 times 1 L cultures in 5 L un-baffled flasks and with a final concentration of 1 mM 6-fluoro-L-tryptophan. Extracts were analyzed by UHPLC-MS and -MS-MS as described before[1], while adjusting the gradient to 10-20 % B over 17 minutes, to get separation of different isomers and multiple halogenated darobactins. The derivatives were named according to the following pattern: "darobactin #" (e.g. darobactin 9), position of fluorine on tryptophan according to IUPAC (e.g. 6F for 6-fluoro-L-tryptophan"), followed by the position of the halogenated tryptophan in the core peptide (e.g. 1, 3 or 7). As an example, D9-6F3 indicates darobactin 9 with a 6-fluoro-L-tryptophan at position 3 (WNW$_F$SKSW).

6. Purification of novel darobactin derivatives

The darobactin derivatives D6, D23, D31 and D36 were purified as described before for DA and D9 using a two-step purification protocol.[1] The previously used Biphenyl column was switched for an XBridge Phenyl column in the first purification step (5 µm, 19x150 mm; Waters Corp.). For D9, the gradient was additionally adjusted to 2-16 % B from minute 2-24 during the second purification step. Detection was accomplished by their respective mass signals (Table S 3) using the built-in single quadrupole mass analyzer of the preparative Autopurifier HPLC-MS system by Waters Corp. Retention times were as follows: D6: C_{18}: 14.3 min, Phenyl: 14 min, D23: C_{18}: 11.8 min, Phenyl: 11.6 min, D31: C_{18}: 13 min, Phenyl: 14.5 min, D36: C_{18}: 12.5 min, Phenyl: 13 min.

D22 is the only derivative, where both purification protocols were used. To highlight improvements in the process, the old protocol used for D22 is described here in detail:

After elution from the absorbent resin XAD-16N with 80 % MeOH and reuptake in H_2O after evaporation, the darobactin crude extracts were purified by three chromatographic steps (two preparative-scale and one semi-preparative scale) using gradients of H_2O (eluent A) and ACN (eluent B) both containing 0.1 % FA. First, crude extracts were separated by preparative reverse-phase (RP) HPLC-MS using an AutoPurification system (Waters Corp.) equipped with built-in single quadrupole mass analyzer. Separation of 900 µL injections was achieved with an XBridge BEH C18 column (130 Å, 5 µm, 19 mm X 150 mm; Waters Corp.) with a gradient of 2-25 % B for D22 over 22 minutes. The second step utilized the same HPLC-MS instrument equipped with a XBridge Phenyl OBD (130 Å, 5 µm, 19 mm X 150 mm; Waters Corp.) and a gradient of 5-15 % B for D22 over 22 minutes. Finally, pure compounds were isolated using an

Ultimate 3000 semi-preparative HPLC (Thermo Scientific) equipped with an XSelect CSH OBD Phenyl-Hexyl column (130 Å, 5 µm, 10 mm X 250 mm; Waters Corp.) and a HCT plus Mass Analyzer (Bruker) and a gradient of 2-10 % B for D22 over 20 minutes. Several chromatographic steps involving diversion of sample to an MS analyzer and the choice of adsorbent resin may limit compound recovery. Therefore, we elected to optimize our procedure for darobactin purification. This method yielded 9.8 mg D22 from 12x 1 L culture (0.8 mg/L). In two different batches with the new purification method for D22, as described below, yields varied from 3.0-3.7 mg/L culture (3.8- to 4.6-fold increase).

The purification protocol was optimized for D9-6F, D37, D38, D22 and D32, adapting the cation-exchange protocol of Tehrani *et al.,*[2] which was then used also for D22 and D32. XAD-16N was exchanged by 2 % (w/V) weak cation exchange resin Dowex MAC-3 (hydrogen form, Sigma Aldrich) during the extraction step. The resin was incubated with the culture for 2 hours on orbital shaker (Orbitron/Multitron, Infors HT) at 160 rpm. Supernatant was decanted and MAC-3 washed with dH_2O, ethyl acetate and eluted using 3-5x 1 L of 2 M ammonia per 200-400 g of resin and decanting the supernatant after each step. For the elution steps, decanted supernatant was also filtrated through paper filter. The solvent was then neutralized on ice with 37 % HCl (Sigma Aldrich) and loaded onto a 130 g C_{18} flash column (CHROMABOND Flash RS 120 C_{18} ec, 40–63 µm) using a peristaltic pump (Ismatec). Salts and highly polar compounds were removed using 3 column volumes (CV) of dH_2O on a Biotage flash (Isolera One), which was followed by elution with 20 CV of 5 % Eluent A (dH_2O + 0.1 % FA) to 20 % Eluent B (ACN + 0.1 % FA), followed by a ramp of 3 CV to 95 % B and 3 CV of 95 %B for cleaning. Detection was performed at 220 and 280 nm UV adsorption. The darobactin-containing fractions were collected and dried using a rotary evaporator. Purification on the preparative Autopurifier HPLC-MS system by Waters Corp was achieved using XBridge C_{18} column (5 µm, 19x150 mm; Waters Corp.) with the following gradients:

After 2 min equilibration at 2 % B and 98 % A, for D32[R], D37 and D38 a gradient from 2-25 % B was used for 22 min, followed by a 2 min ramp to 95 % B, 1 min hold at 95 % B and ramp down to 2 % B again. D32[R] eluted at 11.5 min, D37 at 15.5 min and D38 at 15 min.

D22 was purified using 2 min of equilibration at 5 % B and 95 % A, a separation gradient from 5-15 % B was used for 22 min, followed by a 2 min ramp to 95 % B, 1 min hold at 95 % B and ramp down to 5 % B again. D22 eluted approximately at 15 min.

D9-6F was purified using 2 min of equilibration at 11 % B and 89 % A, a separation gradient from 11-17 % B was used for 22 min, followed by a 2 min ramp to 95 % B, 1 min hold at 95 % B and ramp down to 11 % B again. D9-6F eluted together as mixture of the two isomers approximately at 17.1.

H_2O and ACN (+0.1 % FA) were used as mobile phases A and B for all methods with a flow rate of 25 mL/min. The darobactin-containing fractions were collected and dried using a rotary evaporator.

D36 and D9-6F required a second purification step on an Ultimate 3000 SDLC low pressure gradient system (Dionex) equipped with XSelect Peptide CSH C_{18} column (250x10mm 5µm; Waters Corp.), while for the other derivatives (D6, D23, D31) an Acquity CSH Phenyl-hexyl column (250x10mm 5µm; Waters Corp.) was used. The eluents were H_2O + 0.1 % FA as A and ACN + 0.1 % FA as B, at a flow rate of 5 mL/min (6 mL/min for D9-6F) and a column temperature of 45 °C (35 °C for D32[R]). The darobactins were detected by UV absorption at 280 nm using time-dependent fraction collection.

The following gradients were applied:

For D36 the separation was started with a plateau of 8 % B for 1 min, followed by ramp to 15 % B for 20 min and a cleaning step using a ramp to 95 % B of 1 min, keeping 95 % B for 1 min and ramping back to the starting conditions of 8 % B for 1 min again, followed by re-equilibration for 1 min. Elution occurred at 5.3 min.

For D9-6F the separation was started with a plateau of 11 % B for 1 min, followed by ramp to 17 % B for 20 min and a cleaning step using a ramp to 95 % B of 1 min, keeping 95 % B for 1 min and ramping back to the starting conditions of 11 % B for 1 min again, followed by reequilibration for 1 min. Elution of the two isomers occurred at 12 and 13 min.

For D6 the separation was started with a plateau of 2 % B for 1 min, followed by ramp to 30 % B for 17 min and a cleaning step using a ramp to 95 % B of 3 min, keeping 95 % B for 1 min and ramping back to the starting conditions of 2 % B for 1 min again, followed by reequilibration for 1 min. Elution occurred at 13.5 min.

For D23 the separation was started with a plateau of 2 % B for 1 min, followed by ramp to 10 % B for 20 min and a cleaning step using a ramp to 95 % B of 1 min, keeping 95 % B for 1 min and ramping back to the starting conditions of 2 % B for 1 min again, followed by reequilibration for 1 min. Elution occurred at 17.8 min.

For D31 the separation was started with a plateau of 2 % B for 1 min, followed by ramp to 15 % B for 20 min and a cleaning step using a ramp to 95 % B of 1 min, keeping 95 % B for 1 min and ramping back to the starting conditions of 2 % B for 1 min again, followed by reequilibration for 1 min. Elution occurred at 12.8 min.

After evaporation, the darobactins were obtained as pale yellowish to brownish amorphous solids. Purity was confirmed by uHPLC-MS and NMR.

7. Determination of antibacterial activity

The antibacterial activity of novel darobactins was established by determining the minimum inhibitory concentration (MIC) as previously described for crude extracts and for pure compounds.[1] For determining the activity of selected darobactins against Carbapenem-resistant *Acinetobacter baumannii* (CRAB) in the presence of lung surfactant, the medium for the MIC assay was supplemented with 1% (*v/v*) lung surfactant (Alveofact® 45 mg/mL suspension; phospholipids from bovine lung, reconstituted with provided vehicle).

8. Characterization of darobactin 22

8.1 Maximum tolerated concentration (MTC)

Husbandry of the adult zebrafish was performed according to internal protocols in accordance with the German Animal Welfare Act (§11 Abs. 1 TierSchG). Zebrafish larvae of the wildtype AB line were used for the study and all experiments were done within the first 120 hours post fertilization (hpf). The MTC assay was designed to determine acute and developmental toxicity (genotoxicity) of tested compounds on different embryonic developmental stages of zebrafish larvae.

For this purpose, the dechorinated larvae (1 day post fertilization, dpf) were incubated at 28°C in the presence of various compound concentrations (10, 50, 100, 250 and 500 µg/mL, n=10 per condition) dissolved in 0.3x Danieau's solution (17 mM NaCl, 2 mM KCl, 0.12 mM $MgSO_4$, 1.8 mM $Ca(NO_3)_2$, 1.5 mM HEPES, pH 7.1 - 7.3, and 1.2 µM methylene blue). The embryos were monitored daily and developmental stages, hatching behavior, pigmentation, heartbeat, blood flow, locomotor response, and body shape were observed.[3] If more than one dead larva was found in the untreated control, the experiment was rejected. An embryo was considered *dead* once there was no heartbeat recorded. Images were taken using a Leica M205FA stereomicroscope equipped with a Leica DFC 7000T camera. The assay was terminated when larvae reached 5 dpf (120 hpf).

8.2 Time-kill assay

Overnight bacterial cultures were prepared from a cryo-preserved stock in fresh medium and incubated in a shaking incubator at 180 rpm and at 30 °C for *Acinetobacter baumannii* (*A. baumannii*) strains and 37 °C for *E. coli* strains. The OD_{600} was determined and the initial inoculum was adjusted to approximately 5 x 10^6 colony-forming units (CFU)/mL. Time-kill kinetics were determined at 2x, 4x, 8x MIC of DA and D22, respectively, and compared to vehicle-treated cultures as control. Treated bacterial cultures were incubated in a shaking incubator at 180 rpm at the appropriate temperatures. Aliquots were taken at the assigned time points (0, 0.5, 1, 2, 4, 6, 8 and 24 hours) and CFU/mL were determined by plating the culture

onto non-selective CASO agar in serial dilution. The plates were incubated in a static incubator at the appropriate temperature for 24 hours. The following day the colonies were counted and CFU/mL was determined, which was then plotted against time to obtain a time-kill curve (TKC). All TKCs were done with three technical repeats of two independent biological replicates.

9. NMR spectroscopy of D6, D22, D23, D31, D32, D36, D37 and D38

1D and 2D NMR data were recorded as described previously in a 2:1 mixture of D2O:ACN-d3 + 1 % FA-d2,[1] while modifying the temperature to 308.15 K for D32. NMR signals were assigned using ACD/labs NMR workbook suite. Color-coding in the 2D NMR spectra reflects fit of the experimental chemical shifts to the calculated chemical shifts: Green = experimental value perfectly matches the calculated chemical shift, yellow = experimental value matches predicted area of calculated chemical shift, red = experimental value differs from calculated chemical shift. All structure formulae devised by NMR will be made publicly available under their corresponding name in NPAtlas[4] upon acceptance of the manuscript.

10. Protein expression and purification

Protein expression and purification of the *E. coli* BAM complex was performed as described previously[5] with minor modification. The BAM complex was extracted with 1% (v/v) DDM, and further purified in presence of 0.05%DDM. Finally, the BAM complex protein was purified on a 16/600 Superose 6 increase column using 50 mM Tris pH 7.5, 50 mM NaCl, 0.01% (w/v) n-dodecyl β-D-maltopyranoside (DDM) and concentrated to 5.0 mg/mL. The freshly purified protein was directly used for cryo-EM without freezing.

The BamA barrel domain (BamA-β) (residues 421–810, C690S, C700S), with an N-terminal His6 tag and overexpressed and purified as described previously with small modifications.[5] In brief, *bamA-β* was cloned into pROEX vector and overexpressed in inclusion bodies using Top10. The inclusion bodies were solubilized in 8 M urea and purified using Ni-NTA with 6 M urea. The purified protein was dialyzed against ultrapure H_2O and the precipitated protein was collected by centrifugation at 100,000 xg for 30 min. The remaining soluble fraction was further concentrated using 10 kDa cut-off centrifugal into 1 mL. The remaining soluble fraction was further concentrated using 10 kDa cut-off centrifugal into 1 mL. Next, 0.6 g Guanidine hydrochloride was added and dissolved in the concentrated sample and further used for solubilizing the pellet. The subsequent refolding process was performed as described.[5]

11. Electron microscopy sample preparation and data processing, collecting and analyzing

The BAM complex incubated with a fivefold molar excess of darobactin was applied to a glow-discharged quantifoil R1.2/1.3 AU 300. The grids were blotted for 3-5 s (blot force: minus 5 to 5; 100 % humidity) and plunge-frozen in liquid ethane/propane using a Vitrobot Mark V. The grids were first screened to get the best vitrified grid using Talos Arctica. Then, the best vitrified specimens were imaged on an FEI Titan Krios, operated at 300 kV, and equipped with a Gatan K3 camera. The data were acquired with a defocus range of 0.6-3.0 μm and nominal magnification of 105,000x (a pixel size of 0.85 Å) using Thermo Fisher Scientific EPU software. For individual frames, an electron dose of 1.2 e/A2 was used, corresponding to a cumulative electron dose of 60 e/A2 equally distributed over a 3-sec movie.

The data were processed using relion3.1[6] and the final polished particles were further imported into cryoSPARC.[7] A heterogeneous classification was carried out and the particles from good classes were further submitted for non-uniform refinement to get a final 3D reconstruction map. The summary of data processing is provided in Figure S 1 and Figure S 43. Previously the BAM complex with DA (PDB: 7NRI[5]) was used as initial model to fit chimeraX1.3.[8] The BAM complex was refined in Phenix.[9] The geometry restraints of D9 and D22 were generated by using eLBOW from Phenix. The darobactins and the darobactin binding regions of the BAM complex were further refined with ISOLDE.[10] Validation was done using the cryo-EM validation tools in Phenix. The map resolution range was determined from local resolution calculation in cryoSPARC.

12. Thermal titration calorimetry

The ITC experiments on darobactins binding to BamA-β were carried out at 25 °C with the Microcal PEAQ-ITC instrument in duplicate. The ITC was performed in the buffer solution as described in previous study (20 mM NaPi, 150 mM NaCl, 0.1% w/v LDAO, pH 7.5).[5] 300 μM darobactin in the syringe was injected 13 times into the sample cell containing 20 μM BamA-β with a spacing of 150 s and a stirring rate of 750 rpm. The injection volume is 3 μl with a 6s duration, except for the first injection (0.4 μl, 0.8 s). The control experiment was performed in the absence of BamA-β. The resulting data were analyzed using the integrated public-domain software packages NITPIC, SEDPHAT and GUSSI.

13. List of bacterial strains

Table S 1: List of bacterial strains used or generated in this study.

Bacterial strain	Genotype	Reference
Cloning strains		
E. coli HS996	F^-, *mcr*A, Δ(*mrr*-*hsd*RMS-*mcr*BC), Φ80*lac*ZΔM15, Δ*lac*X74, *rec*A1, *ara*D139, Δ(*ara-leu*)7697, *gal*U, *gal*K, *rps*L (Str^R), *end*A1, *nup*G, *fhu*A::IS2	Invitrogen
E. coli NEB10β	*mcr*A, spoT1Δ(*mrr-hsd*RMS-*mcr*BC), Φ80d(*lac*ZΔM15)*rec*A1, *rel*A1, Δ*lac*X74, *rec*A1, *ara*D139, Δ(*ara-leu*)7697, *gal*K16, *gal*E15, *rps*L (Str^R), *end*A1, *nup*G, *fhu*A	New England Biolabs
E. coli BL21 (DE3)	$F^–$, *ompT, gal, dcm, lon,* Δ$hsdS_B(r_B^- m_B^-)$, λ(DE3 [*lacI lacUV5-T7p07 ind1 sam7 nin5*]), [*malB*$^+$]$_{K\text{-}12}$($λ^S$)	Invitrogen
E. coli BL21-Gold (DE3)	*E. coli* B F– ompT hsdS(rB– mB–) dcm+ Tetr gal λ(DE3) endA The	Agilent Technologies
E. coli HS996 pNOSO-darABCDE-A	*E. coli* HS996 with pNOSO-darABCDE-A, kan^R	Groß *et al.* 2021
E. coli HS996 pNOSO-darABCDE-9	*E. coli* HS996 with pNOSO-darABCDE-9, kan^R	Groß *et al.* 2021
E. coli HS996 pNOSO-darABCDE-6	*E. coli* HS996 with pNOSO-darABCDE-6, kan^R	Groß *et al.* 2021
E. coli NEB10β pNOSO-darABCDE-22	*E. coli* NEB10β with pNOSO-darABCDE-22, kan^R	This work
E. coli NEB10β pNOSO-darABCDE-23	*E. coli* NEB10β with pNOSO-darABCDE-23, kan^R	This work
E. coli NEB10β pNOSO-darABCDE-24	*E. coli* NEB10β with pNOSO-darABCDE-24, kan^R	This work
E. coli NEB10β pNOSO-darABCDE-25	*E. coli* NEB10β with pNOSO-darABCDE-25, kan^R	This work
E. coli NEB10β pNOSO-darABCDE-26	*E. coli* NEB10β with pNOSO-darABCDE-26, kan^R	This work
E. coli NEB10β pNOSO-darABCDE-27	*E. coli* NEB10β with pNOSO-darABCDE-27, kan^R	This work
E. coli NEB10β pNOSO-darABCDE-28	*E. coli* NEB10β with pNOSO-darABCDE-28, kan^R	This work
E. coli HS996 pNOSO-darABCDE-29	*E. coli* HS996 with pNOSO-darABCDE-29, kan^R	This work

Bacterial strain	Genotype	Reference
Cloning strains		
E. coli HS996 pNOSO-darABCDE-30	*E. coli* HS996 with pNOSO-darABCDE-30, kan^R	This work
E. coli HS996 pNOSO-darABCDE-31	*E. coli* HS996 with pNOSO-darABCDE-31, kan^R	This work
E. coli HS996 pNOSO-darABCDE-32	*E. coli* HS996 with pNOSO-darABCDE-32, kan^R	This work
E. coli HS996 pNOSO-darABCDE-36	*E. coli* HS996 with pNOSO-darABCDE-36, kan^R	This work
E. coli HS996 pNOSO-darABCDE-37	*E. coli* HS996 with pNOSO-darABCDE-37, kan^R	This work
E. coli HS996 pNOSO-darABCDE-38	*E. coli* HS996 with pNOSO-darABCDE-38, kan^R	This work
E. coli HS996 pNOSO-darABCDE-39	*E. coli* HS996 with pNOSO-darABCDE-39, kan^R	This work
Heterologous producer strains		
E. coli BL21 (DE3) pNOSO-darABCDE-9	*E. coli* BL21 (DE3) with pNOSO-darABCDE-9, kan^R	Groß *et al.* 2021
E. coli BL21 (DE3) pNOSO-darABCDE-6	*E. coli* BL21 (DE3) with pNOSO-darABCDE-6, kan^R	Groß *et al.* 2021
E. coli BL21 (DE3) pNOSO-darABCDE-22	*E. coli* BL21 (DE3) with pNOSO-darABCDE-22, kan^R	This work
E. coli BL21-Gold (DE3) pNOSO-darABCDE-22	*E. coli* BL21-Gold (DE3) with pNOSO-darABCDE-22, kan^R	This work
E. coli BL21 (DE3) pNOSO-darABCDE-23	*E. coli* BL21 (DE3) with pNOSO-darABCDE-23, kan^R	This work
E. coli BL21 (DE3) pNOSO-darABCDE-24	*E. coli* BL21 (DE3) with pNOSO-darABCDE-24, kan^R	This work
E. coli BL21 (DE3) pNOSO-darABCDE-25	*E. coli* BL21 (DE3) with pNOSO-darABCDE-25, kan^R	This work
E. coli BL21 (DE3) pNOSO-darABCDE-26	*E. coli* BL21 (DE3) with pNOSO-darABCDE-26, kan^R	This work
E. coli BL21 (DE3) pNOSO-darABCDE-27	*E. coli* BL21 (DE3) with pNOSO-darABCDE-27, kan^R	This work

Bacterial strain	Genotype	Reference
Heterologous producer strains		
E. coli BL21 (DE3) pNOSO-darABCDE-28	*E. coli* BL21 (DE3) with pNOSO-darABCDE-28, kan^R	This work
E. coli BL21 (DE3) pNOSO-darABCDE-29	*E. coli* BL21 (DE3) with pNOSO-darABCDE-29, kan^R	This work
E. coli BL21 (DE3) pNOSO-darABCDE-30	*E. coli* BL21 (DE3) with pNOSO-darABCDE-30, kan^R	This work
E. coli BL21 (DE3) pNOSO-darABCDE-31	*E. coli* BL21 (DE3) with pNOSO-darABCDE-31, kan^R	This work
E. coli BL21 (DE3) pNOSO-darABCDE-32	*E. coli* BL21 (DE3) with pNOSO-darABCDE-32, kan^R	This work
E. coli BL21 (DE3) pNOSO-darABCDE-36	*E. coli* BL21 (DE3) with pNOSO-darABCDE-36, kan^R	This work
E. coli BL21 (DE3) pNOSO-darABCDE-37	*E. coli* BL21 (DE3) with pNOSO-darABCDE-37, kan^R	This work
E. coli BL21 (DE3) pNOSO-darABCDE-38	*E. coli* BL21 (DE3) with pNOSO-darABCDE-38, kan^R	This work
E. coli BL21 (DE3) pNOSO-darABCDE-39	*E. coli* BL21 (DE3) with pNOSO-darABCDE-39, kan^R	This work
E. coli BL21 (DE3) C43 pBAM	*E. coli* BL21 (DE3) C43 with pBAM, amp^R	Kaur *et al.* 2021
E. coli BL21 (λ DE3) Lemo cells pET15b-BamAβ421-810	*E. coli* BL21 (λ DE3) Lemo cells with pET15b-BamAβ421-810, amp^R	This work

Supplementation figures and tables

Cryo-EM of BAM-D9

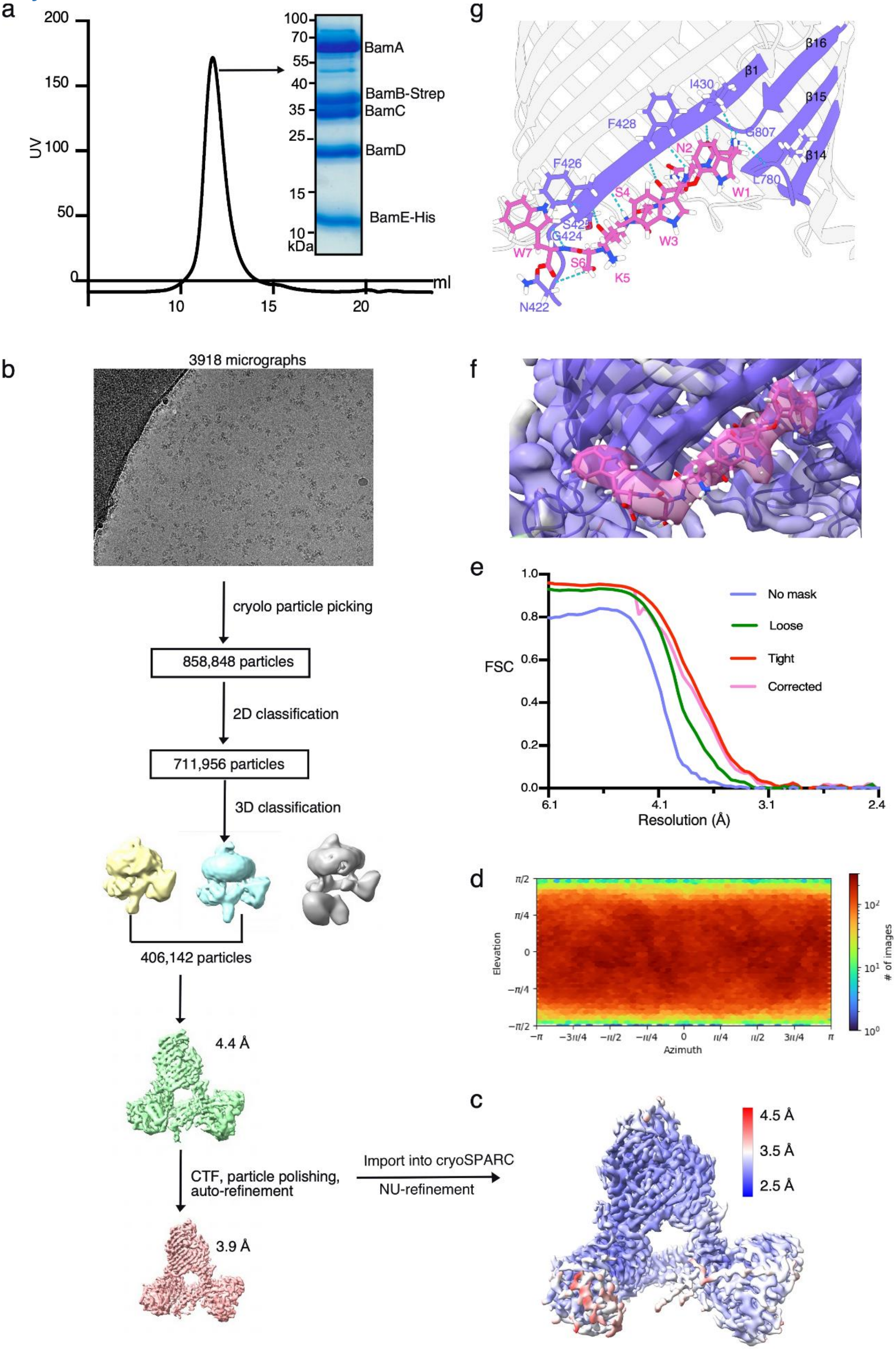

Figure S 1: Cryo-EM structure of BAM-D9 complex.a, SDS page analysis of purified BAM sample used for cryo-EM. b, Workflow of data generation, processing and refinement to decipher BAM-D9 complex. c, BAM overview map with resolution of the EM reconstruction, highlighting local variations from 2.5 Å (blue) to 4.5 Å (red). d, angular distribution of D9 bound to BAM complex was calculated in cryoSPARC for particle projections. Heat map shows number of particles for each viewing angle. e, resolution estimation by Fourier shell correlation (FSC) curves (CryoSparc). f, magnified BAM-D9 interaction area showing electron density surface of D9 (pink) bound on BAM (blue). g, magnified BAM-D9 interaction area, D9 (pink) and interacting amino acids of BamA (blue) are highlighted in stick representation. Potential hydrogen bond interactions are shown as green dashes. Involved ß-sheets (ß1, ß14, ß15, ß16) were labeled as interaction amino acids of BamA and D9.

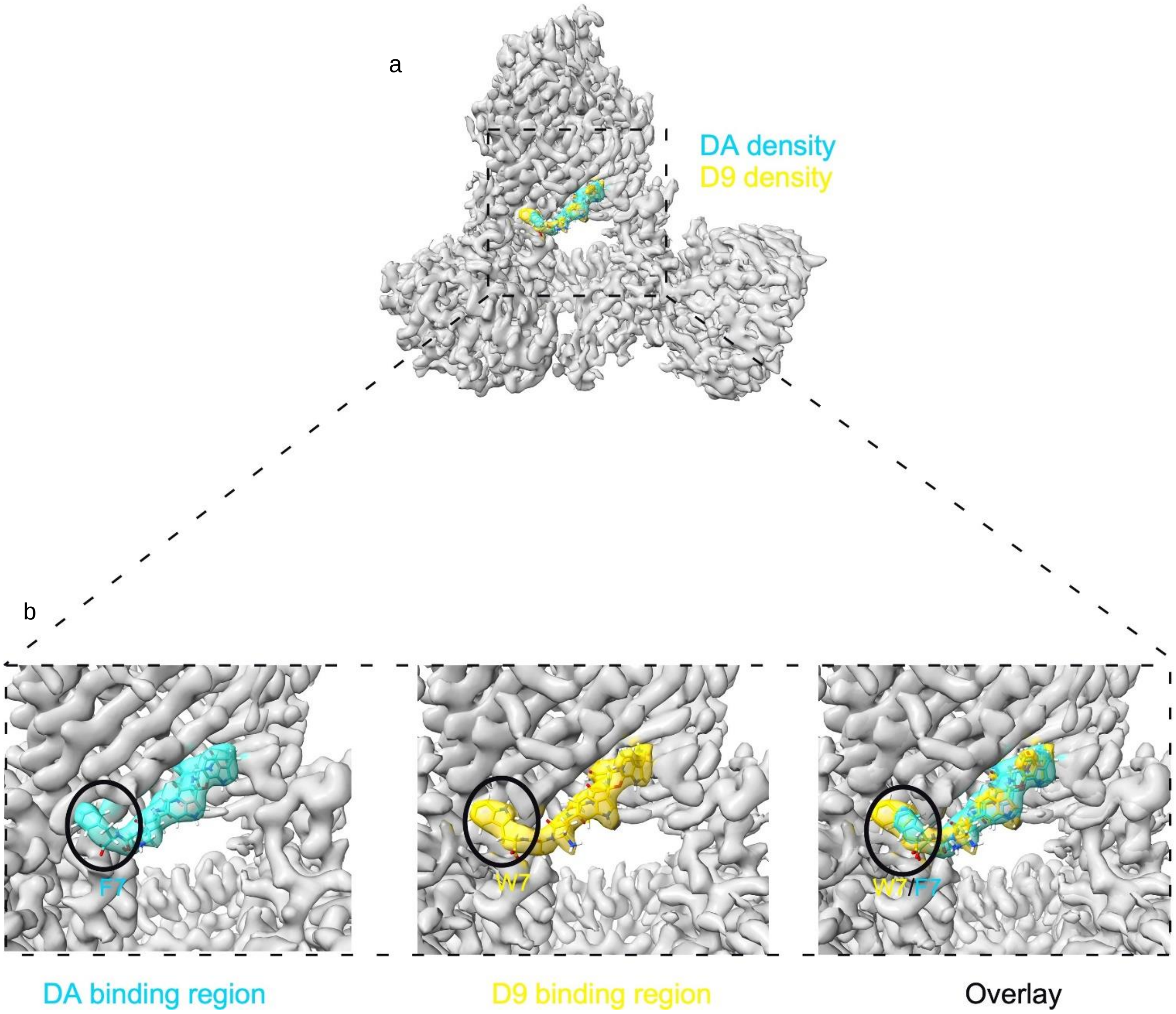

Figure S 2: EM density comparison of DA and D9. a, Overlay of BAM-DA (blue) and BAM-D9 (yellow) electron density. For the density maps, the raw data of BAM-DA were taken from Kaur *et al.*.[5] The raw data of BAM-D9 were generated in this study. b, Magnified view of DA, D9 and overlaid binding region. DA and D9 are fitted (stick presentation) to show consistent matching of DA and D9 into the electron densities.

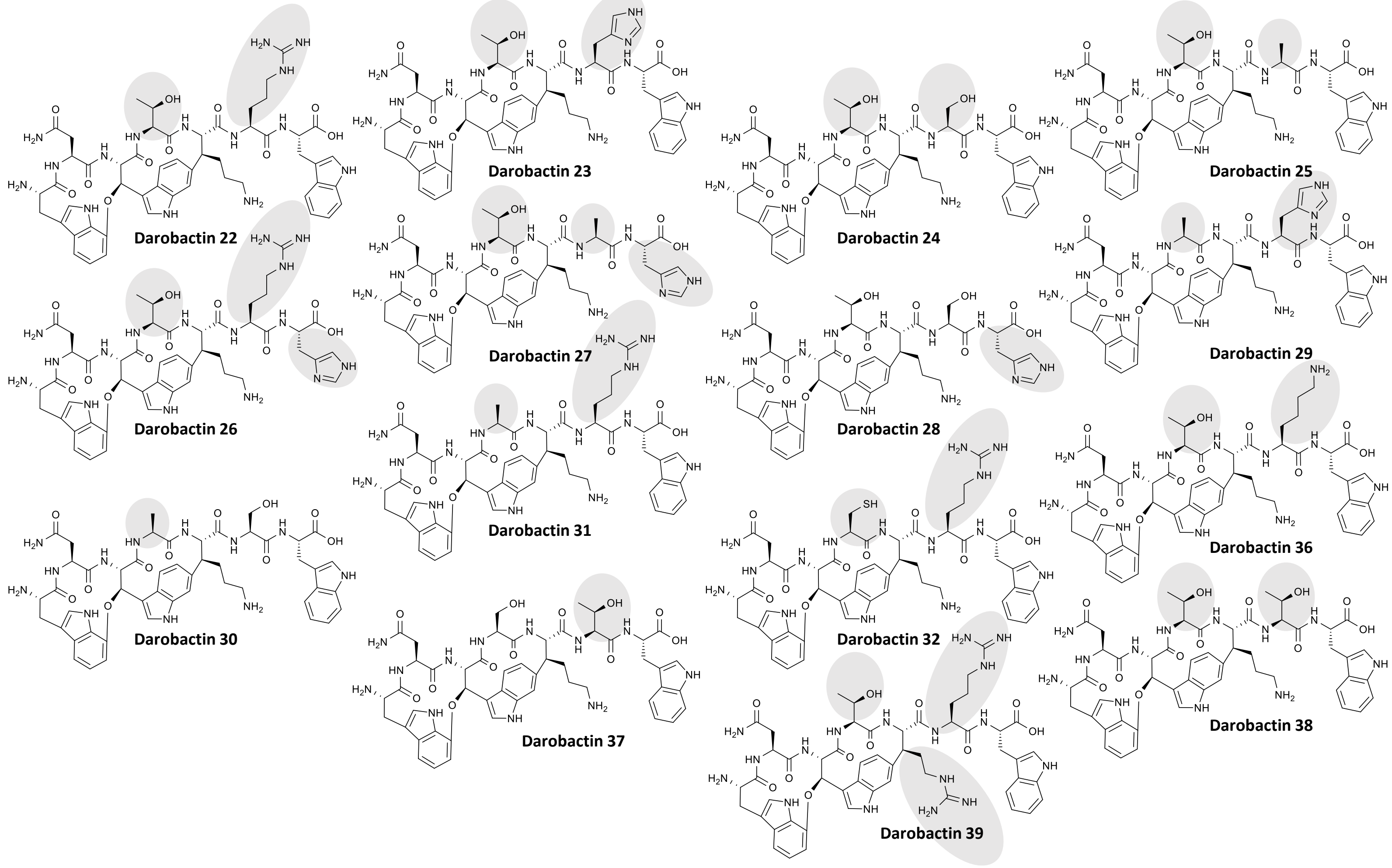

Darobactin	Core peptide
9	W N W S K S W
22	W N W **T** K **R** W
23	W N W **T** K **H** W
24	W N W **T** K S W
25	W N W **T** K **A** W
26	W N W **T** K **R H**
27	W N W **T** K **A H**
28	W N W **T** K S **H**
29	W N W **A** K **H** W
30	W N W **A** K S W
31	W N W **A** K **R** W
32	W N W **C** K **R** W
36	W N W **T** K **K** W
37	W N W S K **T** W
38	W N W **T** K **T** W
39	W N W **T R R** W

Figure S 3 : Overview of all generated non-halogenated novel darobactin derivatives. D22 to D32 and D36 to D39 are displayed and positions of hepta-peptide with amino acid changes compared to D9 amino acid sequence were highlighted in grey. Table with core peptide sequences itemize differences between the darobactin derivatives to D9.

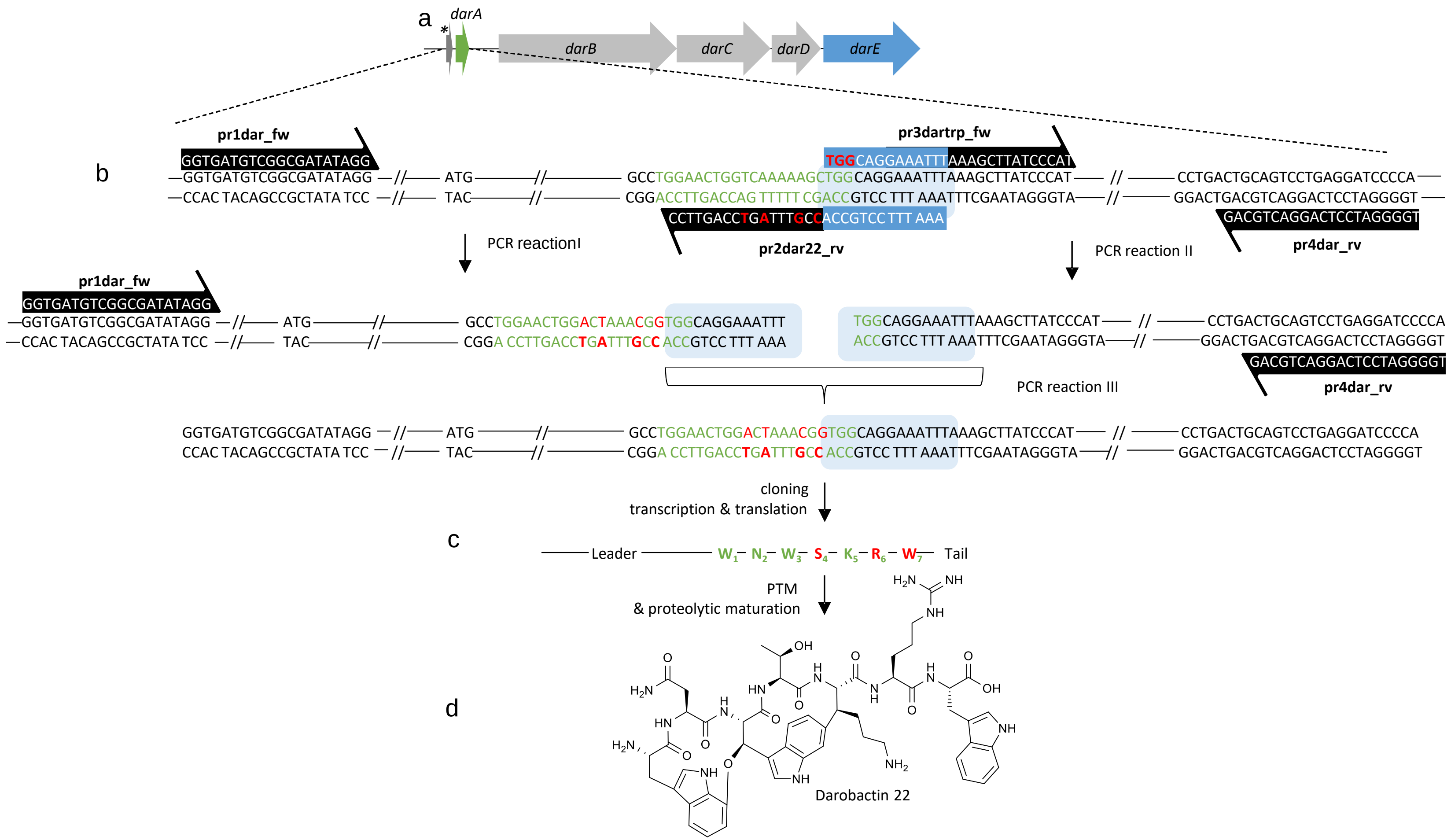

Figure S 4: Generation of new darobactin derivatives via overlap-extension PCR. a, Biosynthetic gene cluster of D9 b, Exemplified PCR reaction workflow with primer pr1dar_fw and pr2dar22_rv (harboring mismatches (red) compared to template sequence to introduce mutations during PCR amplification, leading into D22) to generate DNA fragment of PCR reaction I and with pr3dartrp_fw and pr4dar_rv to generate DNA fragment of PCR reaction II. Primer pr2dar22_rv and pr3dartrp_fw sharing homologous region (highlighted in blue). Using DNA fragments of PCR reaction I & II as template, overlap extension PCR reaction, named as PCR reaction III, was performed to generate fragment harboring modified *darA* core sequence. Unique restriction sites on 3' and 5'end enable to clone the mutated *darA* gene into pNOSO-darABCDE-9 for generating pNOSO-darABCDE-22. c, core peptide sequence of D22 with changed amino acids (red) compared to DA/D9. d, D22 structure based on NMR data, page 48-53.

Table S 2: Oligonucleotides used for overlap extension (OE) polymerase chain reaction I, II and III. Homologous regions of pr3darhis_fw and pr3dartrp_fw with pr2darXX_rv are highlighted in blue. Mismatches of pr2darXX_rv compared to template to generate the new darobactin derivatives are highlighted in red.

Oligonucleotide	Sequence (5'-3')
pr1dar_fw	**GGTGATGTCGGCGATATAGG**
pr3darhis-fw	CAT**CAGGAAATTTAAAGCTTATCCCAT**
pr3dartrp-fw	TGG**CAGGAAATTTAAAGCTTATCCCAT**
pr4dar_rv	**TGGGGATCCTCAGGACTGCAG**
pr2dar22_rv	**AAATTTCCTGCCACCGTTTAGTCCAGTTCC**
pr2dar23_rv	**AAATTTCCTGCCAATGTTTAGTCCAGTTCC**
pr2dar24_rv	**AAATTTCCTGCCATGATTTAGTCCAGTTCC**
pr2dar25_rv	**AAATTTCCTGCCACGCTTTAGTCCAGTTCC**
pr2dar26_rv	**AAATTTCCTGATGCCGTTTAGTCCAGTTCC**
pr2dar27_rv	**AAATTTCCTGATGCGCTTTAGTCCAGTTCC**
pr2dar28_rv	**AAATTTCCTGATGTGATTTAGTCCAGTTCC**
pr2dar29_rv	**AAATTTCCTGCCAATGTTTCGCCCAGTTCC**
pr2dar30_rv	**AAATTTCCTGCCATGATTTCGCCCAGTTCC**
pr2dar31_rv	**AAATTTCCTGCCACCGTTTCGCCCAGTTCC**
pr2dar32_rv	**AAATTTCCTGCCACCGTTTACACCAGTTCC**
pr2dar36_rv	**AAATTTCCTGCCATTTTTTAGTCCAGTTCC**
pr2dar37_rv	**AAATTTCCTGCCAAGTTTTTGACCAGTTCC**
pr2dar38_rv	**AAATTTCCTGCCAAGTTTTAGTCCAGTTCC**
pr2dar39_rv	**AAATTTCCTGCCACCGCCGAGTCCAGTTCCA**

Table S 3: Calculated and observed masses of the darobactin derivatives. Core peptide sequence of each novel darobactin derivative in comparison to core sequence of DA and D9 are summarized (differences highlighted in red). Calculated and observed masses in the XAD16-N extracts of the different charge states ($[M+H]^{1+}$, $[M+2H]^{2+}$, $[M+3H]^{3+}$, $[M+3H-NH_3]^{3+}$). For D6 and D32, a mass shift of 95.5126 for the $[M+2H]^{2+}$ mass (191.0252 neutral mass), was observed depicted as cysteine (C[R]) and derivatives with residue D6[R] and D32[R]. When reducing with 10 % β-mercaptoethanol, the expected masses are found (D6 and D32). For some derivatives only selected charge states could be found.

Darobactin	Core peptide	Calc. mass $[M+1H]^{1+}$	Obs. mass $[M+1H]^{1+}$	Calc. mass $[M+2H]^{2+}$	Obs. mass $[M+2H]^{2+}$	Calc. mass $[M+3H]^{3+}$	Obs. mass $[M+3H]^{3+}$	Calc. mass $[M+3H-NH_3]^{3+}$	Obs. mass $[M+3H-NH_3]^{3+}$
DA	W N W S K S F	966.4104	966.4111	483.7089	483.7095	322.8083	322.8082	317.1328	317.1324
D6[R]	W N W C[R] K S F	1173.4128	1173.4123	587.2101	587.2102	391.8091	391.8088	386.1336	no
D6	W N W C K S F	982.3876	982.3865	491.6974	491.6970	328.1341	no	322.4585	no
D9	W N W S K S W	1005.4213	1005.4209	503.2143	503.2139	335.8120	335.8118	330.1364	no
D9-6F7	W N W S K S W_F	1023.4119	no	512.2096	512.2096	341.8088	no	336.1333	no
D9-6F1	W_F N W S K S W	1023.4119	1023.4103	512.2096	512.2095	341.8088	341.8084	336.1333	336.1338
D9-6F1-7	W_F N W S K S W_F	1041.4025	1041.4020	521.2049	521.2043	347.8057	347.8052	342.1302	no
D9-5Cl3	W N W_{Cl} S K S W	1039.3824	1039.3802	520.1948	520.1942	347.1323	no	341.4568	no
D9-5Cl7	W N W S K S W_{Cl}	1039.3824	1039.3811	520.1948	520.1937	347.1323	347.1309	341.4568	no
D22	W N W **T** K **R** W	1088.5061	1088.5430	544.7567	544.7912	363.5069	363.5297	357.8314	357.8540
D23	W N W **T** K **H** W	1069.4639	1069.4644	535.2356	535.2457	357.1595	357.1662	351.4840	351.4906
D24	W N W **T** K S W	1019.4370	1019.4790	510.2221	510.2450	340.4838	no	334.8083	no
D25	W N W **T** K **A** W	1003.4421	1003.4520	502.2247	502.2415	335.1522	no	329.4767	no
D26	W N W **T** K **R** H	1039.4857	1039.4851	520.2465	520.2463	347.1667	347.1667	341.4912	341.4911
D27	W N W **T** K **A** H	954.4217	954.4226	477.7145	477.7146	318.8121	318.8121	313.1366	313.1366
D28	W N W **T** K S H	970.4166	970.4174	485.7119	485.7122	324.1437	324.1439	318.4682	318.4683
D29	W N W **A** K **H** W	1039.4533	1039.4545	520.2303	520.2308	347.1560	347.1563	341.4804	341.4808
D30	W N W **A** K S W	989.4264	989.4256	495.2169	495.2169	330.4803	no	324.8048	no
D31	W N W **A** K **R** W	1058.4955	1058.4998	529.7514	529.7537	353.5034	353.5047	347.8278	347.8292
D32[R]	W N W **C**[R] K **R** W	1281.4928	no	641.2500	641.2489	427.8358	427.8351	422.1603	422.1597
D32	W N W **C** K **R** W	1090.4676	1090.4712	545.7374	545.7376	364.1607	364.1613	358.4852	358.4854
D36	W N W **T** K **K** W	1060.4999	1060.4988	530.7536	530.7539	354.1715	354.1716	348.4960	348.4961
D37	W N W **S** K **T** W	1019.4370	1019.4356	510.2221	510.2206	340.4838	340.4834	334.8083	334.8085
D38	W N W **T** K **T** W	1033.4526	1033.4515	517.2300	517.2299	345.1557	345.1554	339.4802	339.4795
D39	W N W **T R R W**	1116.5122	no	558.7597	558.7597	372.8423	372.8422	367.1667	367.1667

Chromatograms of darobactin extracts

This section displays either extracted ion chromatogram (EIC) traces of the most abundant $[M+2H]^{2+}$ ion for darobactin derivatives, with similar ionization behavior compared to DA. For some derivatives (D22, D26, D31, D39) the additional introduced arginine led to the $[M+3H]^{3+}$ being more abundant. To keep comparability, combined extracted ion chromatograms (EICs) of the two charge states were used to determine AUC. The combined EICs are depicted in red, the corresponding BPCs of the extracts in black.

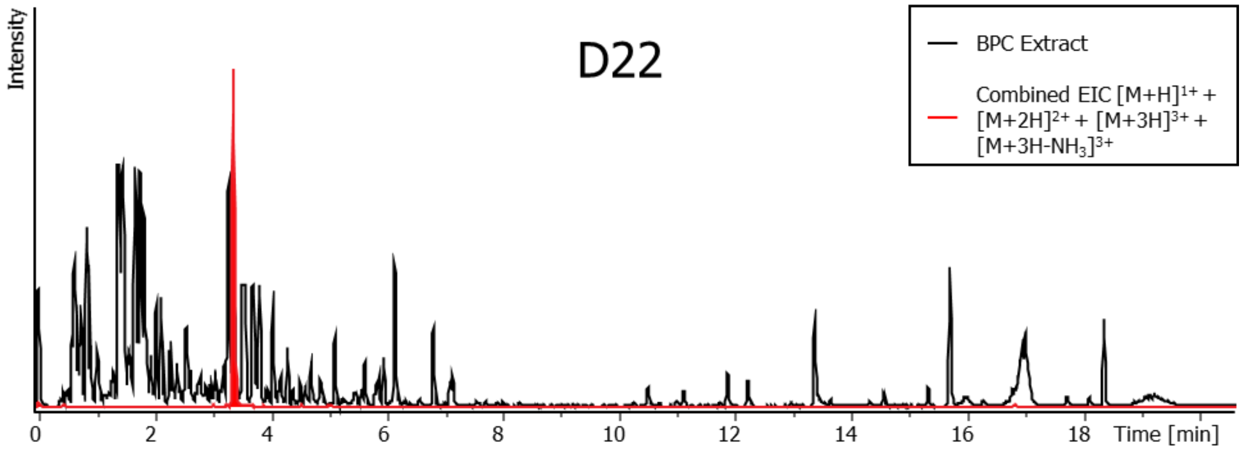

Figure S 5: Chromatogram of the *E. coli* BL21 (DE3) pNOSO-darABCDE-22 culture XAD16-N extract. The red trace displays the D22 combined EIC for the $[M+H]^{1+}$, $[M+2H]^{2+}$, $[M+3H]^{3+}$ and $[M+3H-NH_3]^{3+}$ species at their respective calculated masses (Table S 3) ±0.05 Da. The black trace displays the BPC of the whole extract from fermentation supernatant.

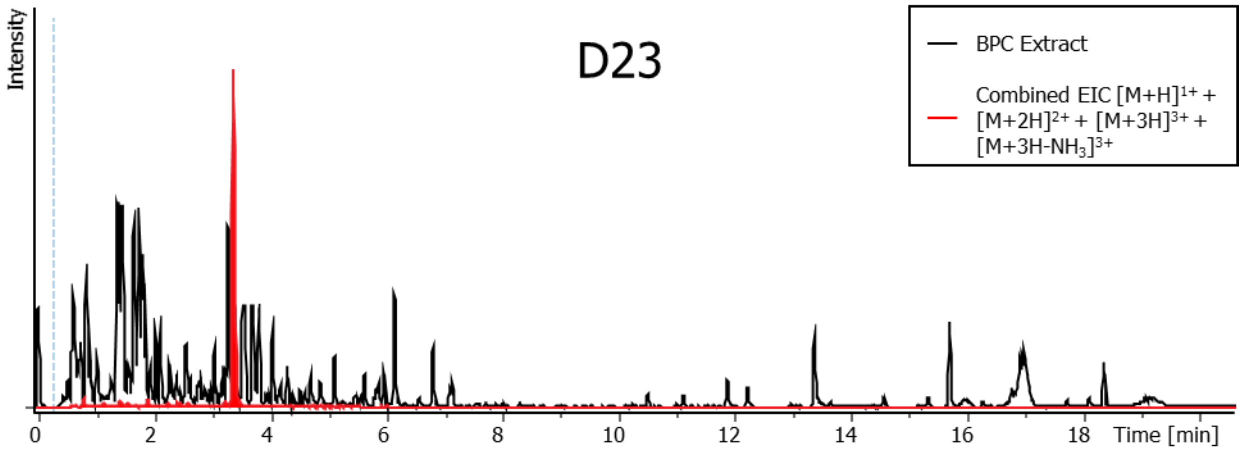

Figure S 6: Chromatogram of the *E. coli* BL21 (DE3) pNOSO-darABCDE-23 culture XAD16-N extract. The red trace displays the D23 combined EIC for the $[M+H]^{1+}$, $[M+2H]^{2+}$, $[M+3H]^{3+}$ and $[M+3H-NH_3]^{3+}$ species at their respective calculated masses (Table S 3) ±0.02 Da. The black trace displays the BPC of the whole extract from fermentation supernatant.

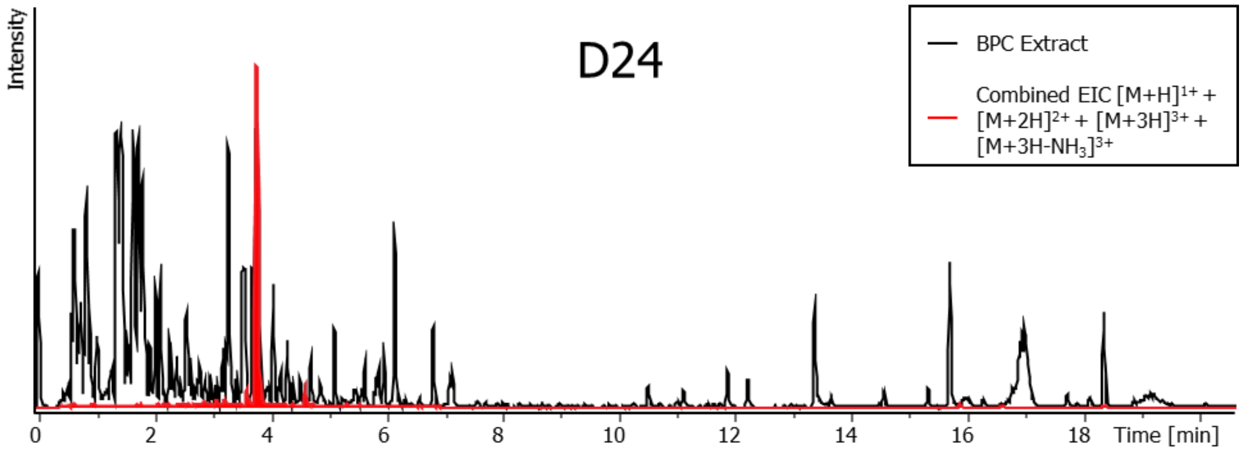

Figure S 7: Chromatogram of the *E. coli* BL21 (DE3) pNOSO-darABCDE-24 culture XAD16-N extract. The red trace displays the D24 combined EIC for the $[M+H]^{1+}$, $[M+2H]^{2+}$, $[M+3H]^{3+}$ and $[M+3H-NH_3]^{3+}$ species at their respective calculated masses (Table S 3) ±0.05 Da. The black trace displays the BPC of the whole extract from fermentation supernatant.

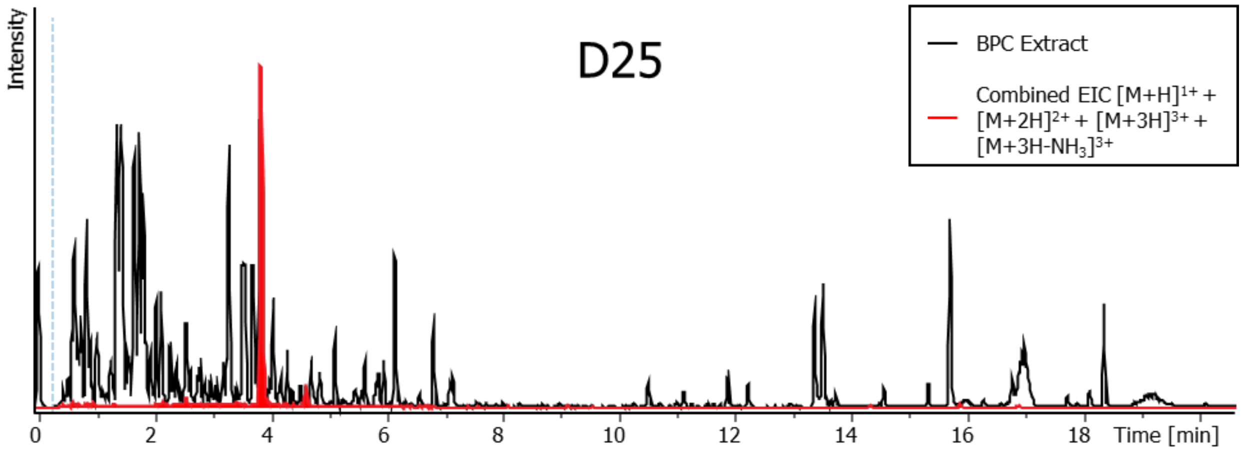

Figure S 8: Chromatogram of the *E. coli* BL21 (DE3) pNOSO-darABCDE-25 culture XAD16-N extract. The red trace displays the D25 combined EIC for the $[M+H]^{1+}$, $[M+2H]^{2+}$, $[M+3H]^{3+}$ and $[M+3H-NH_3]^{3+}$ species at their respective calculated masses (Table S 3) ±0.02 Da. The black trace displays the BPC of the whole extract from fermentation supernatant.

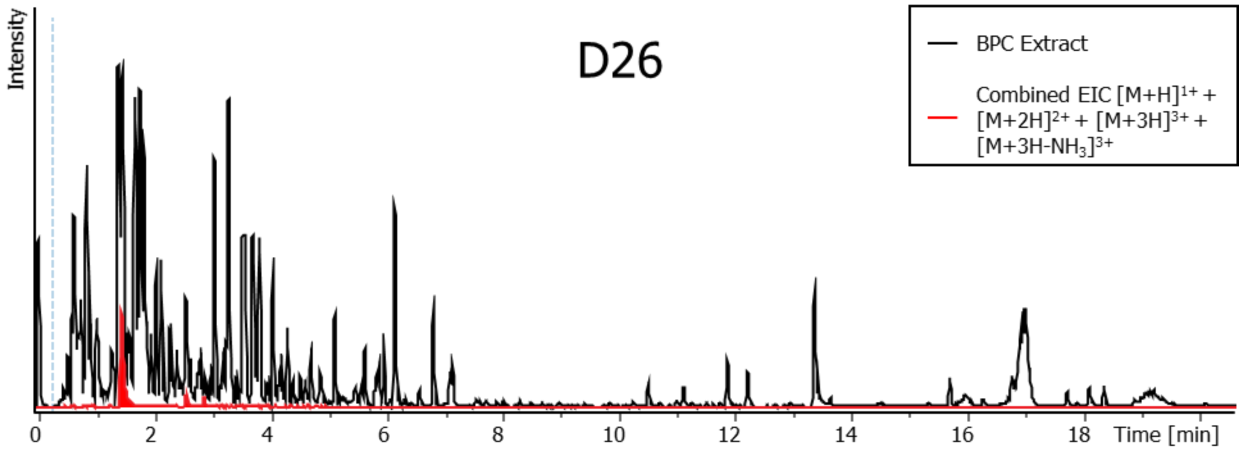

Figure S 9: Chromatogram of the *E. coli* BL21 (DE3) pNOSO-darABCDE-26 culture XAD16-N extract. The red trace displays the D26 combined EIC for the $[M+H]^{1+}$, $[M+2H]^{2+}$, $[M+3H]^{3+}$ and $[M+3H-NH_3]^{3+}$ species at their

respective calculated masses (Table S 3) ±0.02 Da. The black trace displays the BPC of the whole extract from fermentation supernatant.

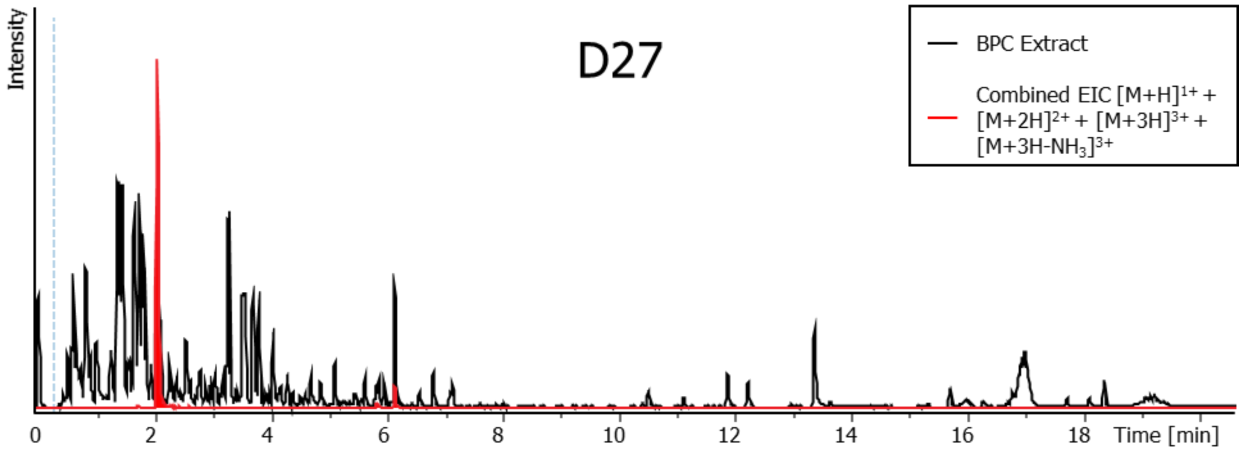

Figure S 10: Chromatogram of the *E. coli* BL21 (DE3) pNOSO-darABCDE-27 culture XAD16-N extract. The red trace displays the D27 combined EIC for the $[M+H]^{1+}$, $[M+2H]^{2+}$, $[M+3H]^{3+}$ and $[M+3H-NH_3]^{3+}$ species at their respective calculated masses (Table S 3) ±0.02 Da. The black trace displays the BPC of the whole extract from fermentation supernatant.

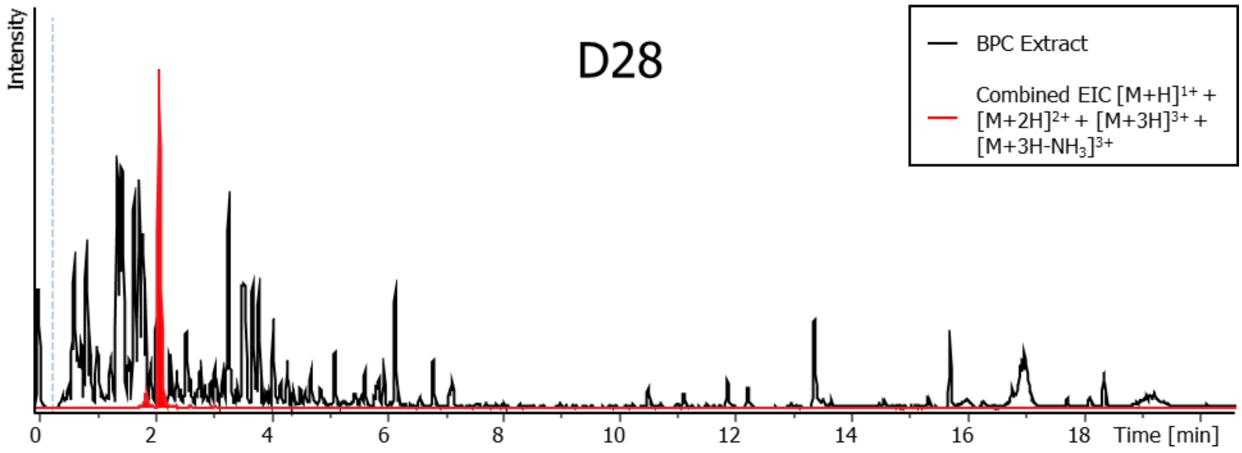

Figure S 11: Chromatogram of the *E. coli* BL21 (DE3) pNOSO-darABCDE-28 culture XAD16-N extract. The red trace displays the D28 combined EIC for the $[M+H]^{1+}$, $[M+2H]^{2+}$, $[M+3H]^{3+}$ and $[M+3H-NH_3]^{3+}$ species at their respective calculated masses (Table S 3) ±0.02 Da. The black trace displays the BPC of the whole extract from fermentation supernatant.

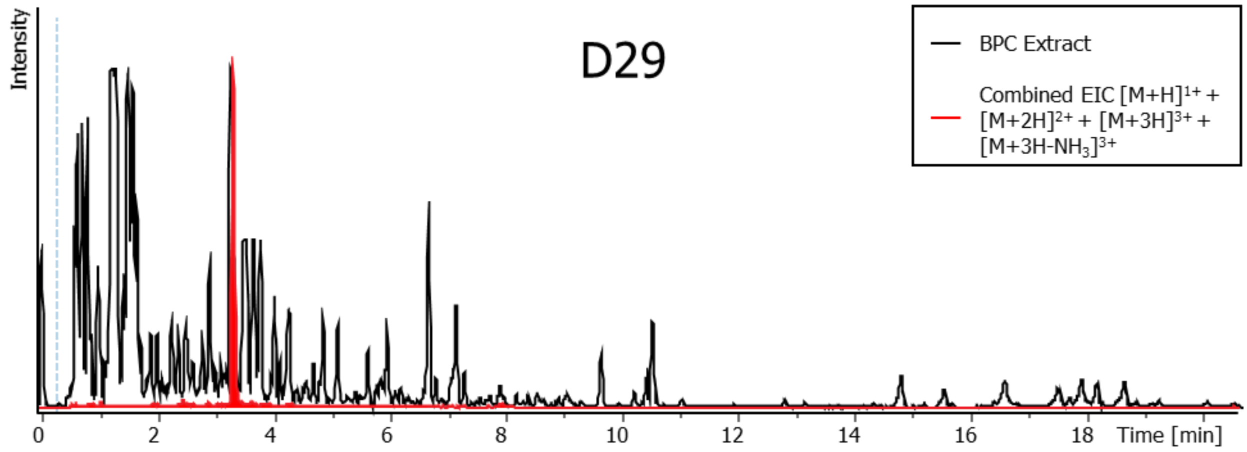

Figure S 12: Chromatogram of the *E. coli* BL21 (DE3) pNOSO-darABCDE-29 culture XAD16-N extract. The red trace displays the D29 combined EIC for the $[M+H]^{1+}$, $[M+2H]^{2+}$, $[M+3H]^{3+}$ and $[M+3H-NH_3]^{3+}$ species at their respective calculated masses (Table S 3) ±0.02 Da. The black trace displays the BPC of the whole extract from fermentation supernatant.

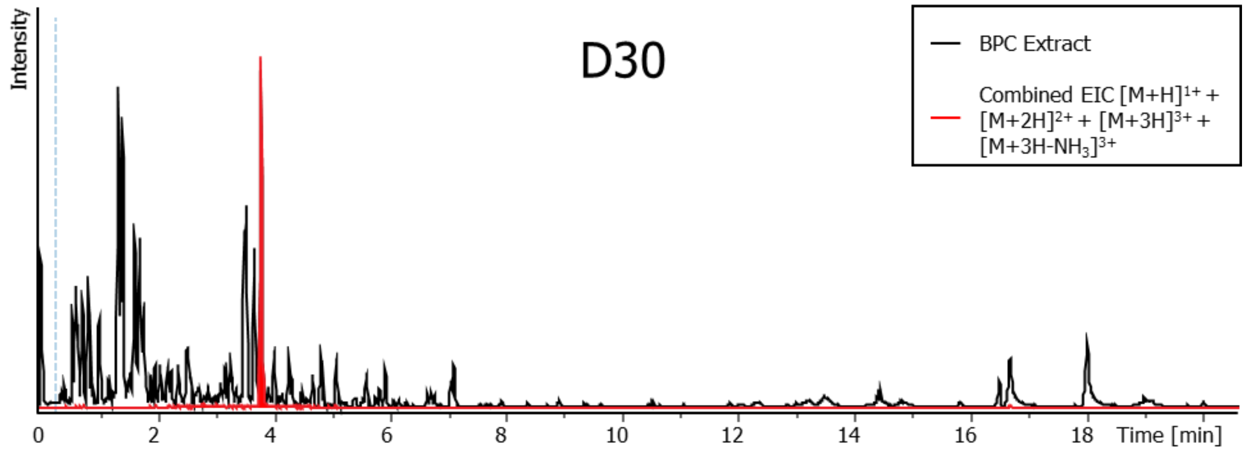

Figure S 13: Chromatogram of the *E. coli* BL21 (DE3) pNOSO-darABCDE-30 culture XAD16-N extract. The red trace displays the D30 combined EIC for the $[M+H]^{1+}$, $[M+2H]^{2+}$, $[M+3H]^{3+}$ and $[M+3H-NH_3]^{3+}$ species at their respective calculated masses (Table S 3) ±0.02 Da. The black trace displays the BPC of the whole extract from fermentation supernatant.

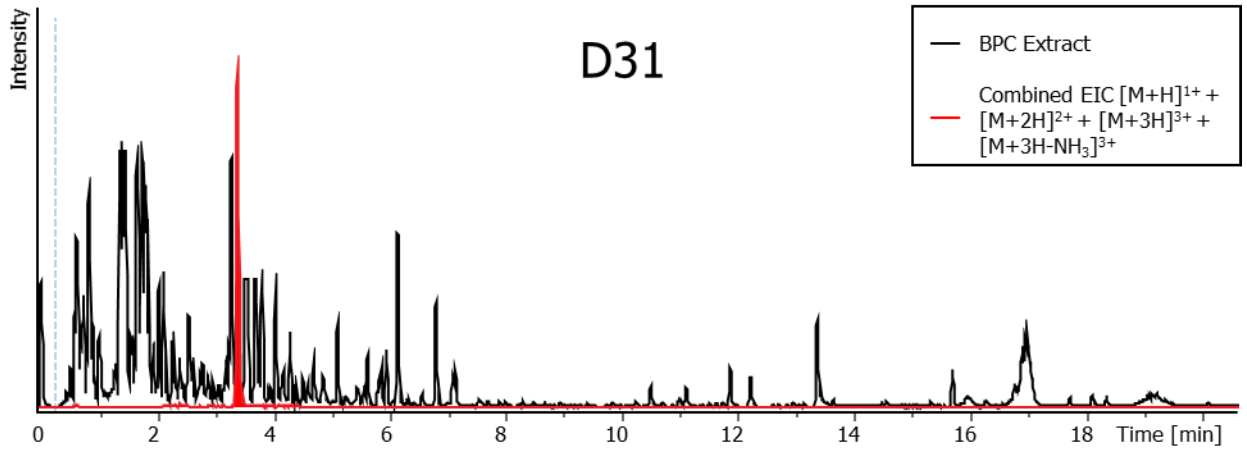

Figure S 14: Chromatogram of the *E. coli* BL21 (DE3) pNOSO-darABCDE-31 culture XAD16-N extract. The red trace displays the D31 combined EIC for the $[M+H]^{1+}$, $[M+2H]^{2+}$, $[M+3H]^{3+}$ and $[M+3H-NH_3]^{3+}$ species at their respective calculated masses (Table S 3) ±0.02 Da. The black trace displays the BPC of the whole extract from fermentation supernatant.

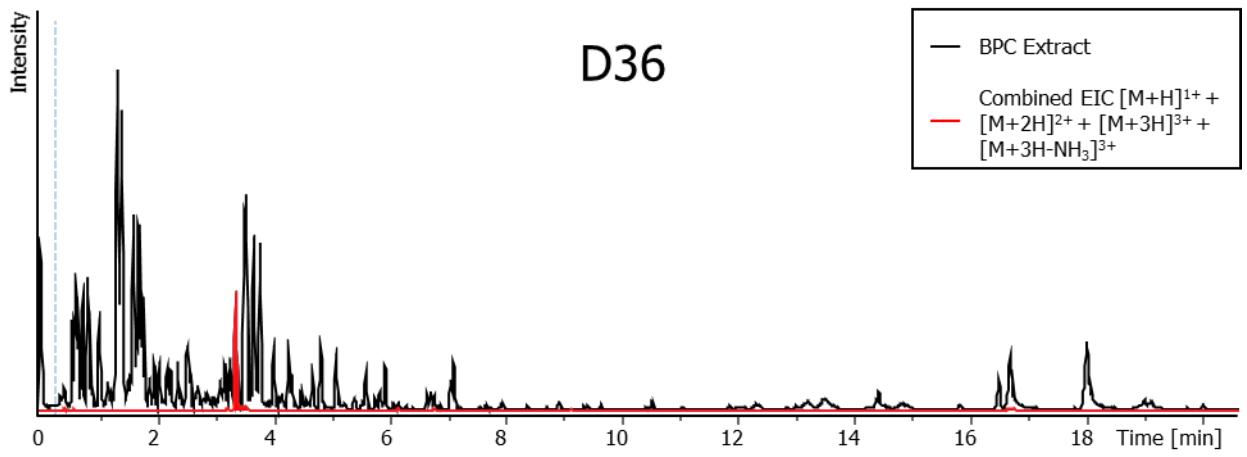

Figure S 15: Chromatogram of the *E. coli* BL21 (DE3) pNOSO-darABCDE-36 culture XAD16-N extract. The red trace displays the D36 combined EIC for the $[M+H]^{1+}$, $[M+2H]^{2+}$, $[M+3H]^{3+}$ and $[M+3H-NH_3]^{3+}$ species at their respective calculated masses (Table S 3) ±0.02 Da. The black trace displays the BPC of the whole extract from fermentation supernatant.

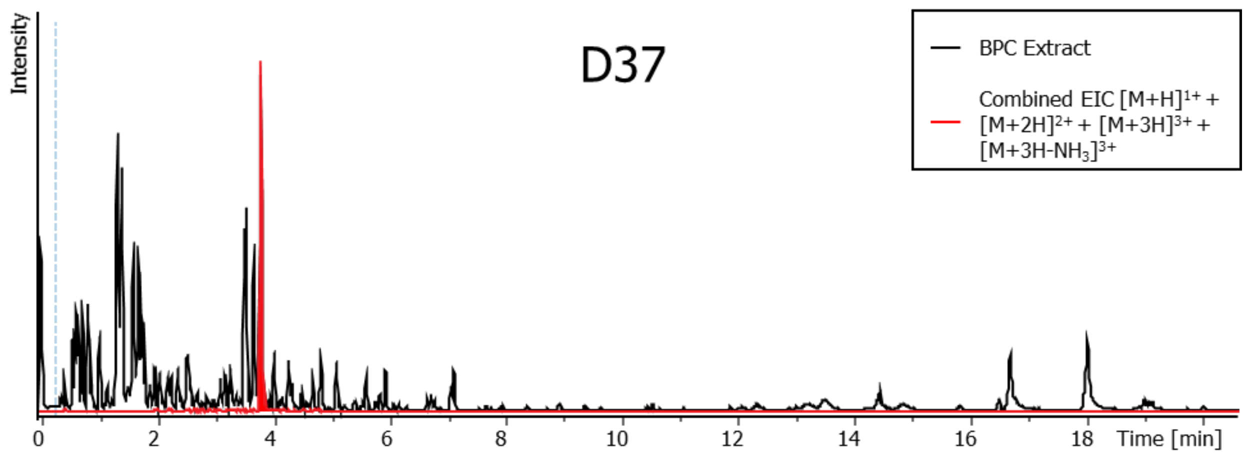

Figure S 16: Chromatogram of the *E. coli* BL21 (DE3) pNOSO-darABCDE-37 culture XAD16-N extract. The red trace displays the D37 combined EIC for the $[M+H]^{1+}$, $[M+2H]^{2+}$, $[M+3H]^{3+}$ and $[M+3H-NH_3]^{3+}$ species at their respective calculated masses (Table S 3) ±0.02 Da. The black trace displays the BPC of the whole extract from fermentation supernatant.

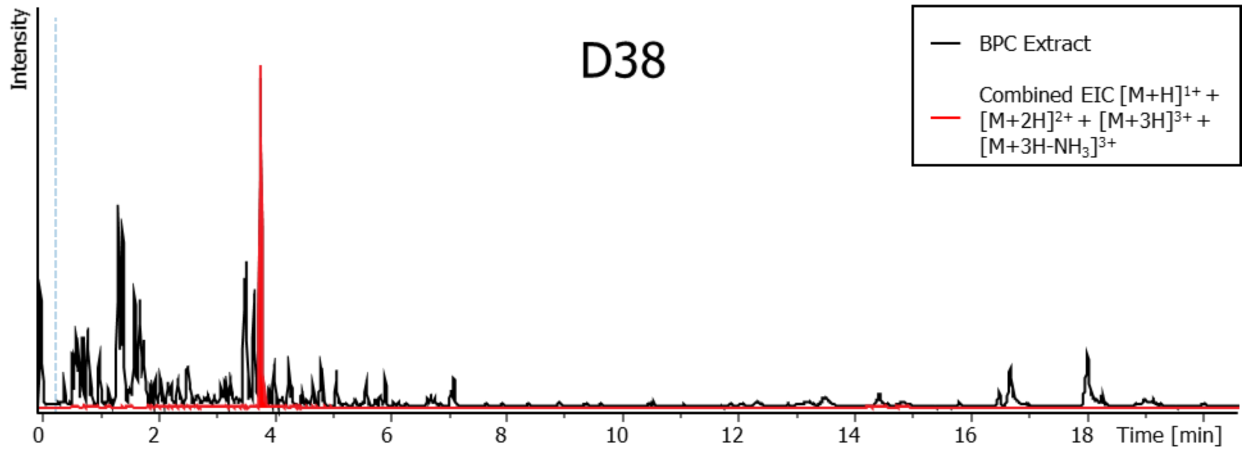

Figure S 17: Chromatogram of the *E. coli* BL21 (DE3) pNOSO-darABCDE-38 culture XAD16-N extract. The red trace displays the D38 combined EIC for the $[M+H]^{1+}$, $[M+2H]^{2+}$, $[M+3H]^{3+}$ and $[M+3H-NH_3]^{3+}$ species at their respective calculated masses (Table S 3) ±0.02 Da. The black trace displays the BPC of the whole extract from fermentation supernatant.

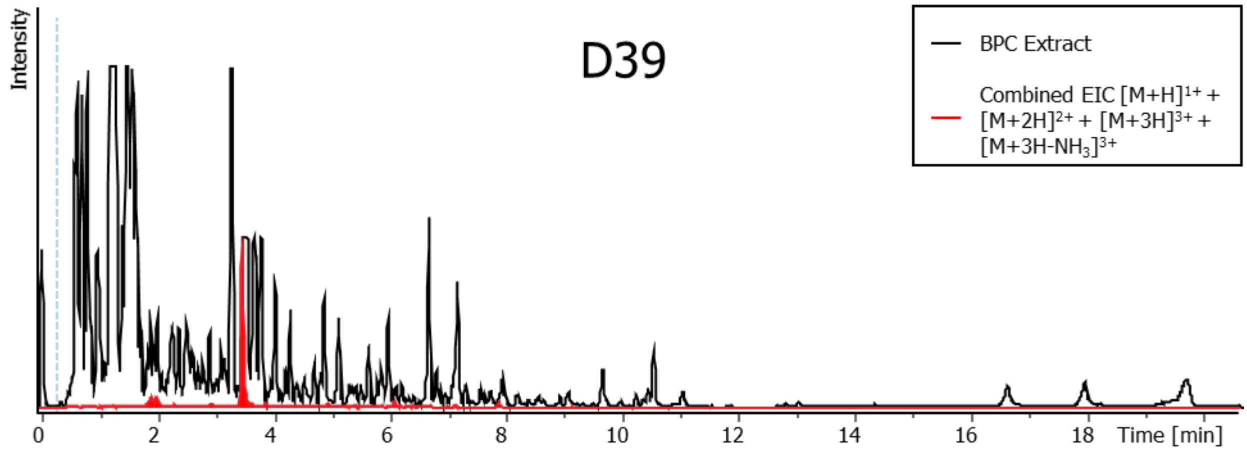

Figure S 18: Chromatogram of the *E. coli* BL21 (DE3) pNOSO-darABCDE-39 culture XAD16-N extract. The red trace displays the D39 combined EIC for the $[M+H]^{1+}$, $[M+2H]^{2+}$, $[M+3H]^{3+}$ and $[M+3H-NH_3]^{3+}$ species at their respective calculated masses (Table S 3) ±0.02 Da. The black trace displays the BPC of the whole extract from fermentation supernatant.

Analyses of darobactin derivative MS2 spectra

Here, all MS2 spectra of the derivatives are displayed. The blue square symbols the precursor ion, which was picked for fragmentation. In all spectra, the $[M-NH_3+2H]^{2+}$ fragment and the typical b2-ion with ammonia loss (theoretical mass of 300.0979) can be observed. Fragmentation pattern is shown in detail for the halogenated derivatives (Figure S 38 and Figure S 40).

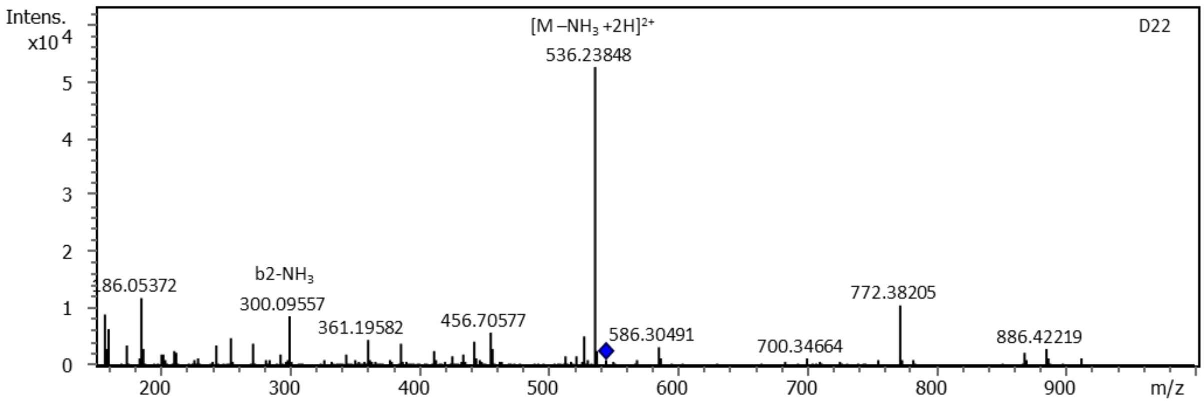

Figure S 19: MS2 spectrum of D22.

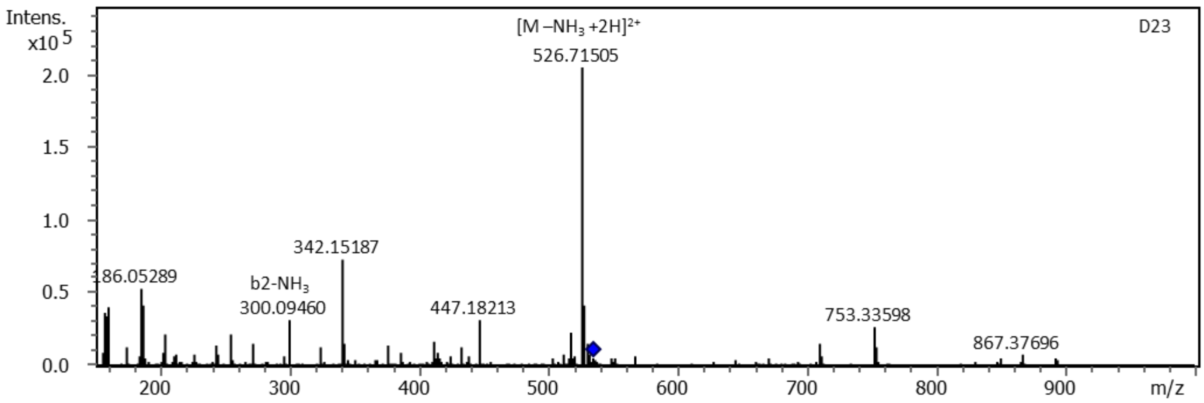

Figure S 20: MS2 spectrum of D23.

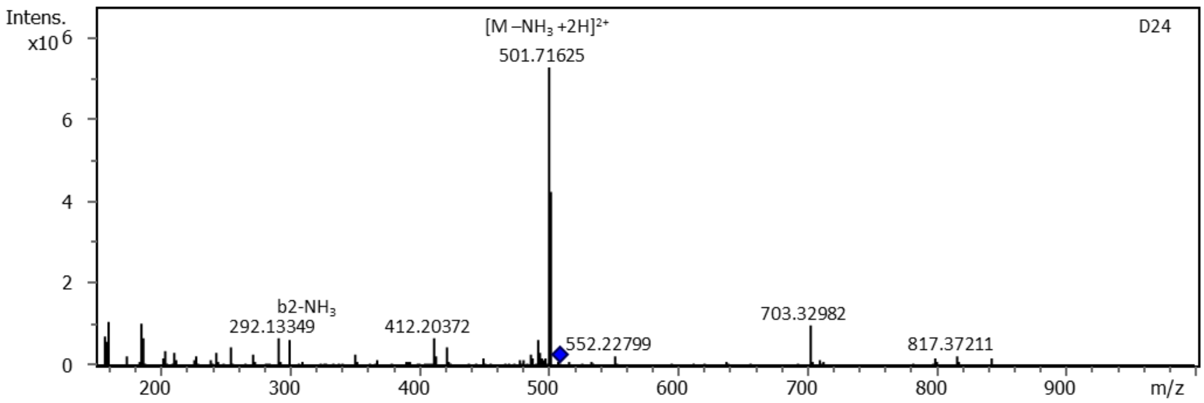

Figure S 21: MS2 spectrum of D24.

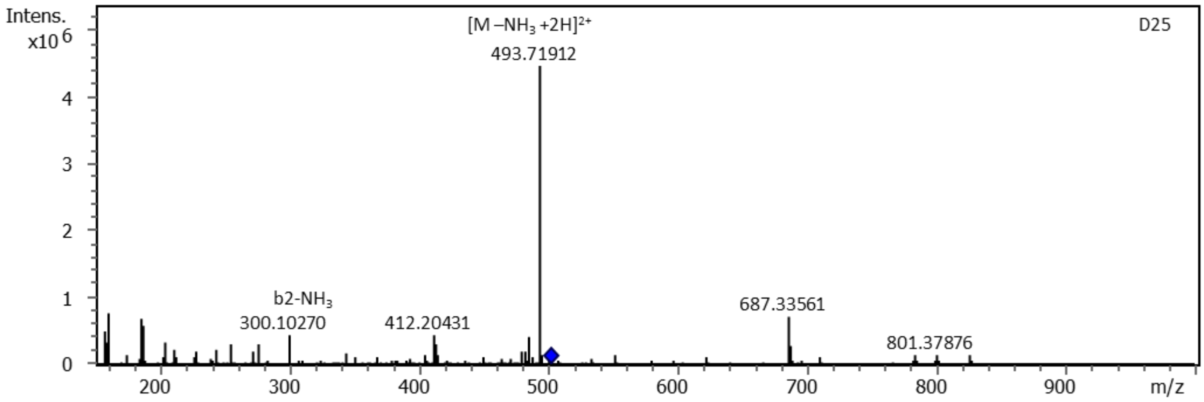

Figure S 22: MS2 spectrum of D25.

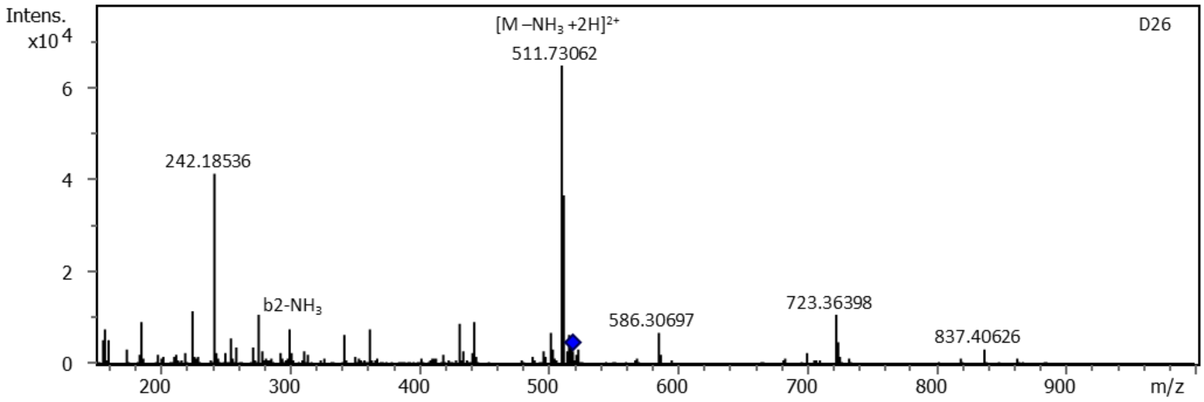

Figure S 23: MS2 spectrum of D26.

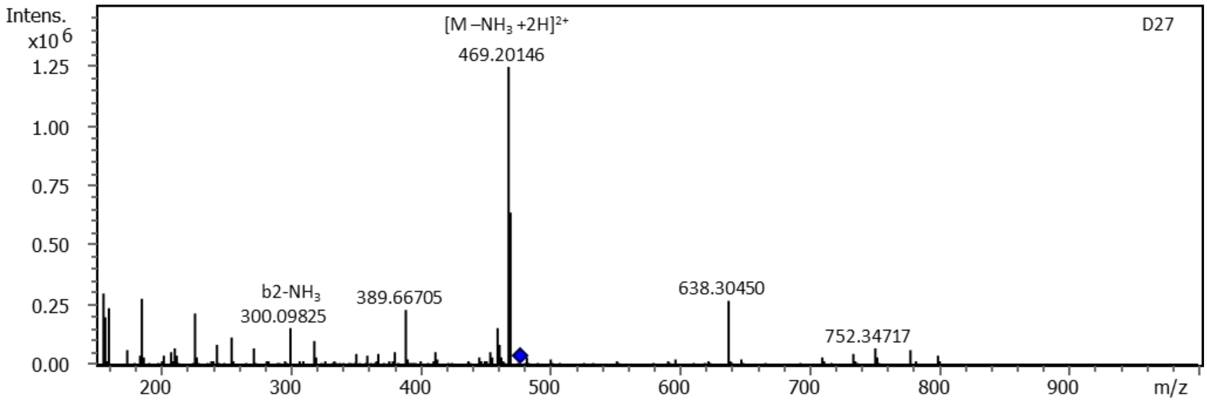

Figure S 24: MS2 spectrum of D27.

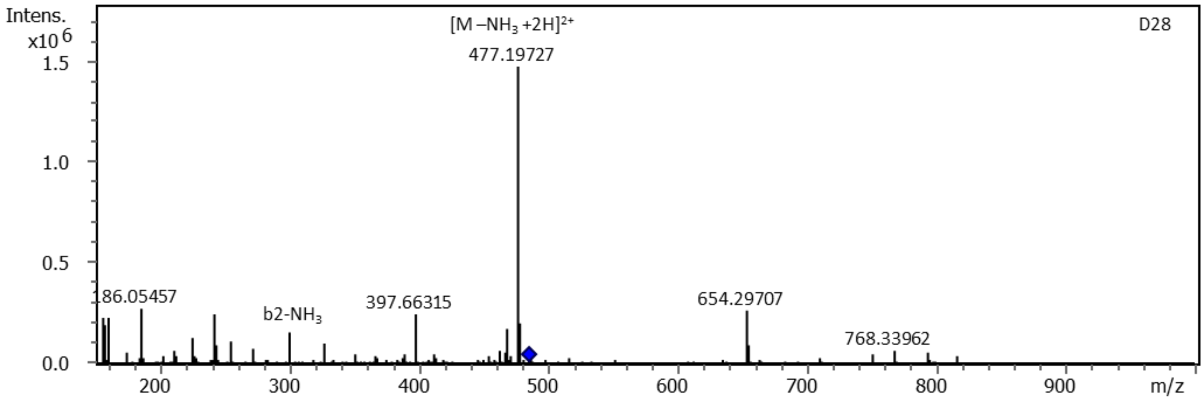

Figure S 25: MS2 spectrum of D28.

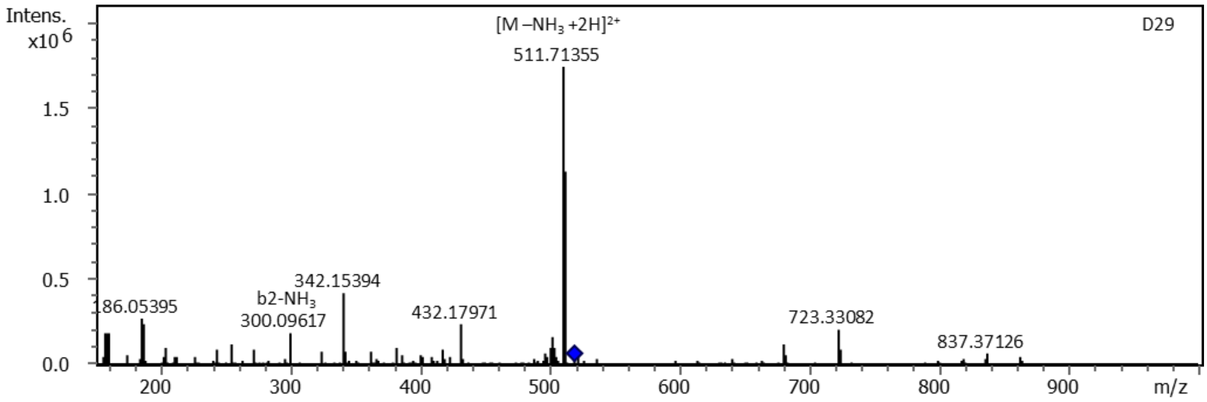

Figure S 26: MS2 spectrum of D29.

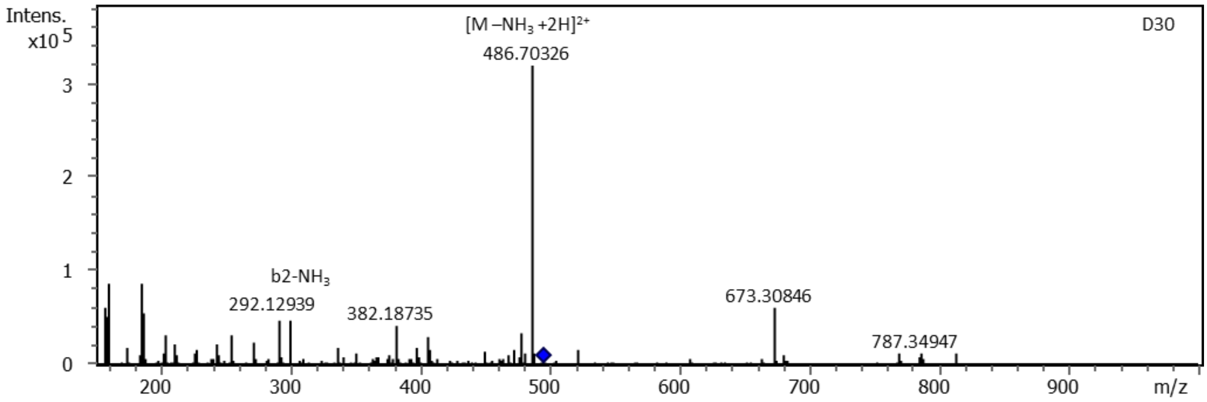

Figure S 27: MS2 spectrum of D30.

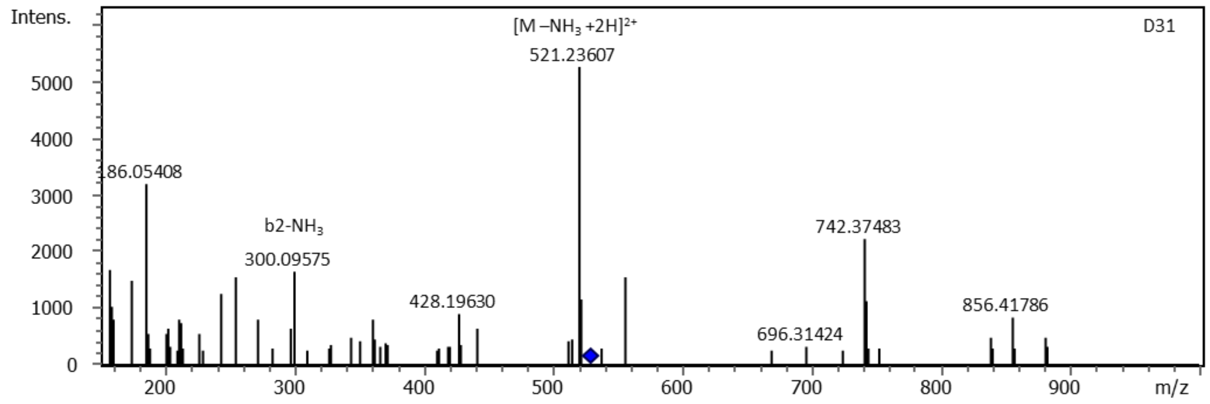

Figure S 28: MS2 spectrum of D31.

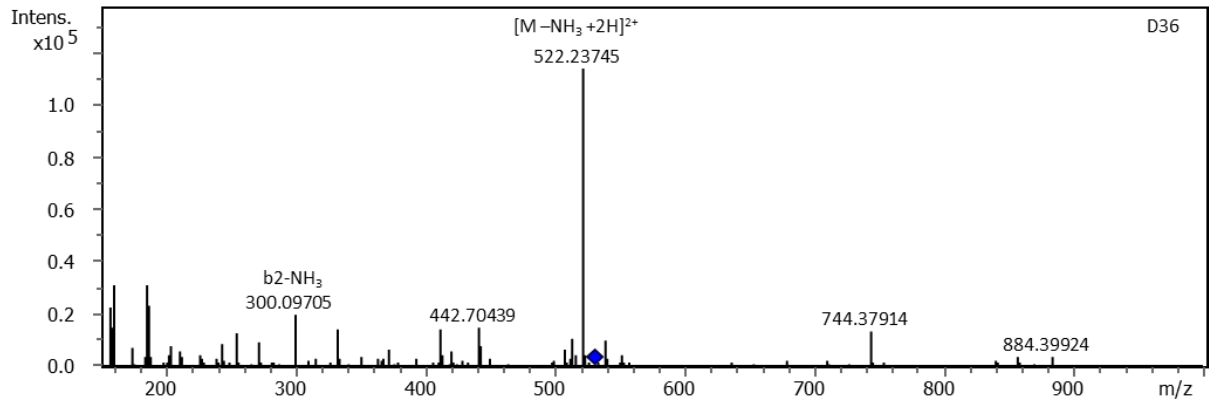

Figure S 29: MS2 spectrum of D36.

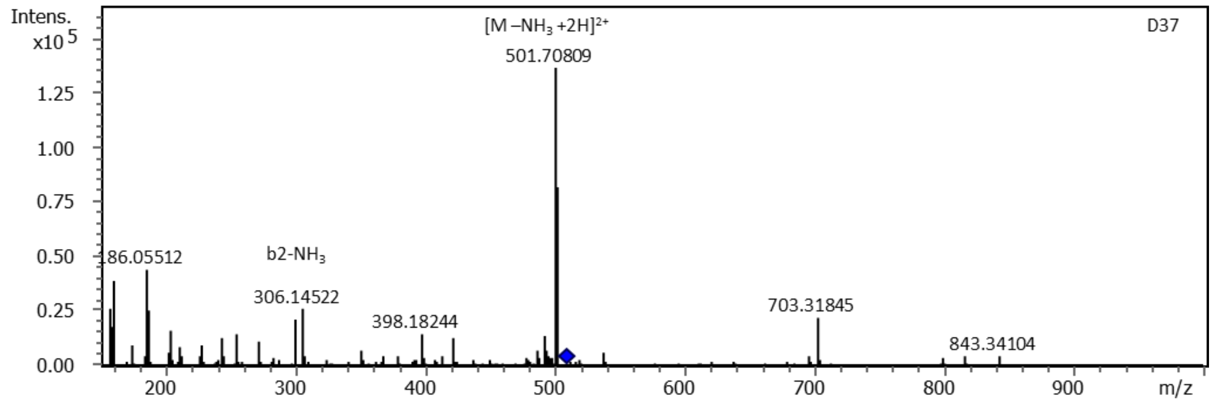

Figure S 30: MS2 spectrum of D37.

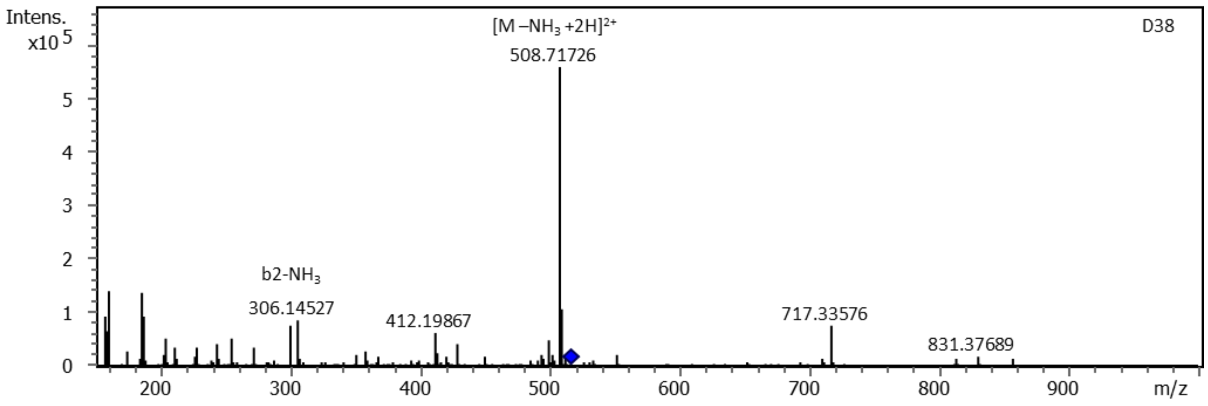

Figure S 31: MS2 spectrum of D38.

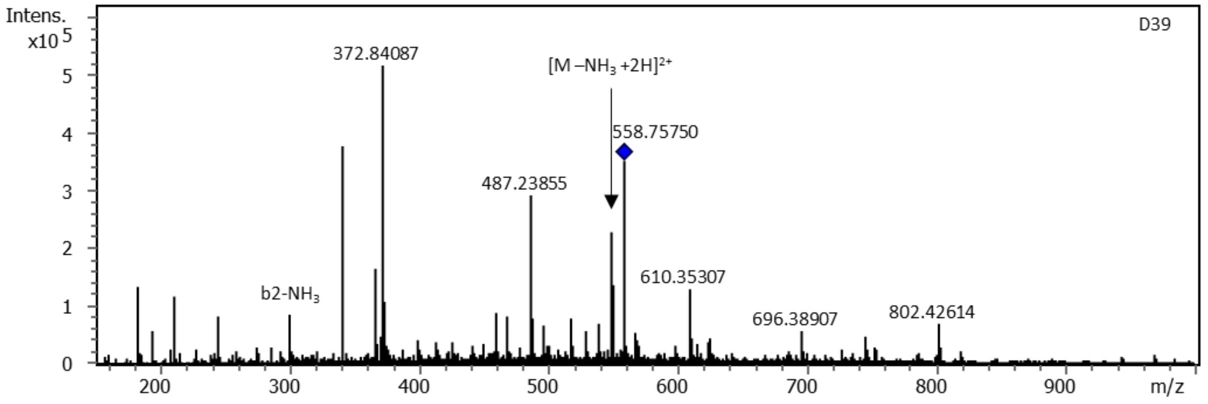

Figure S 32: MS2 spectrum of D39.

Analyses of cysteine derivatives D6 and D32 MS and MS2 spectra

The cysteine derivatives D6 and D32 had an expected mass shift of 95.5126 for the $[M+2H]^{2+}$ mass (191.0252 neutral mass), as observed before.[1] Treatment with 10 % β-mercaptoethanol and heating to 85 °C for 10 min results in appearance of the expected mass (Figures S 33 and 35). Judging from the higher AUC of the reduced D6[R] and D32[R] (Figure S 33 and Figure S 35, b), it is possible, that different, unreported protection groups are present. The main protection group, which was identified using NMR (Figure S 76 - Figure S 85) consists of cysteine and lactic acid. Chirality of lactic acid was deemed unimportant for our conclusions, based only on the overall length of position 4 sidechain, which was prolonged through residue.

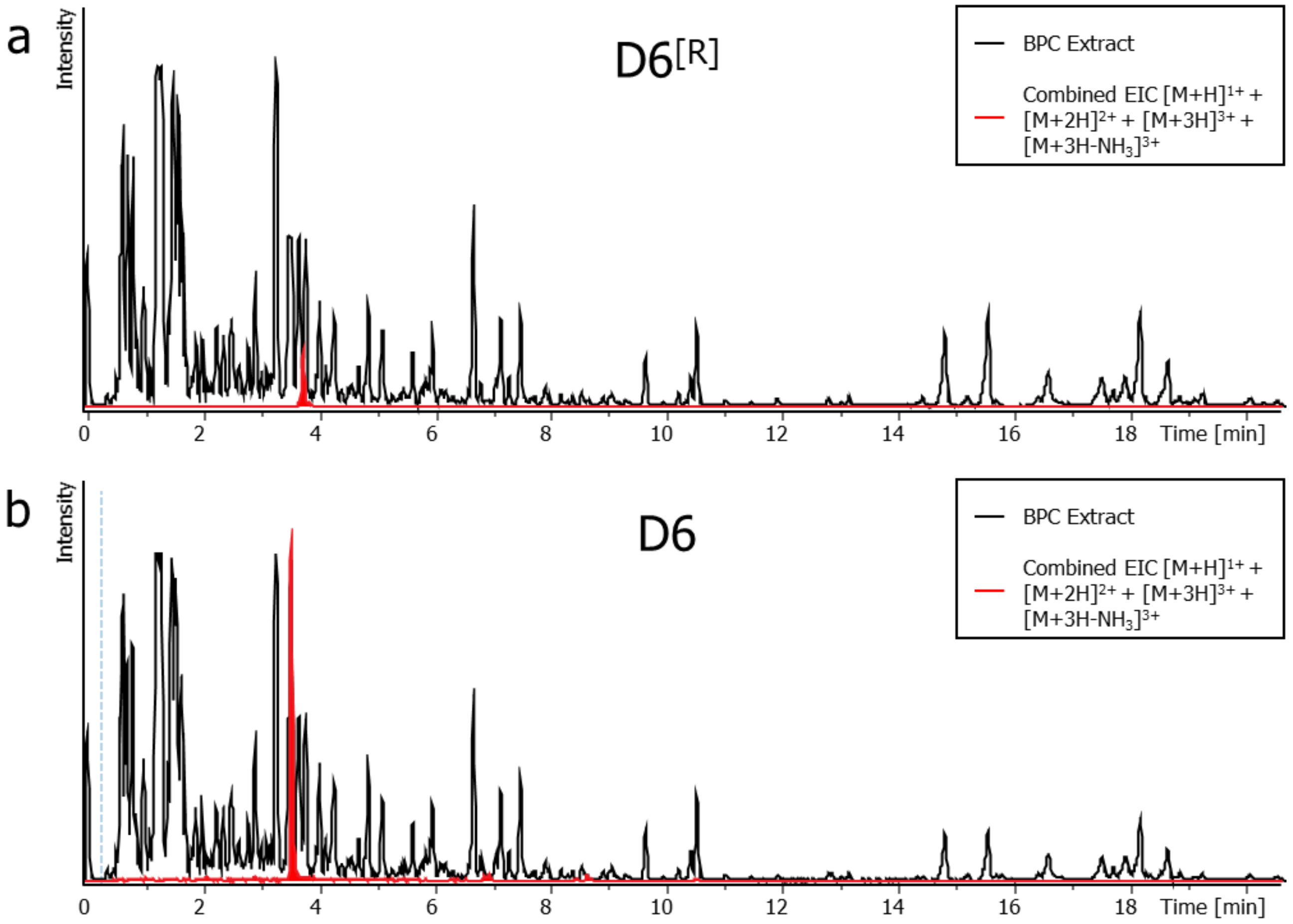

Figure S 33: Chromatogram of the *E. coli* BL21 (DE3) pNOSO-darABCDE-6 culture XAD16-N extract. The red trace displays the D6 combined EIC for the $[M+H]^{1+}$, $[M+2H]^{2+}$, $[M+3H]^{3+}$ and $[M+3H-NH_3]^{3+}$ species with expected protection group [R] on cysteine (a) and without protection group via reduction (b) at their respective calculated masses (Table S 3) ±0.02 Da. A displays the 1:5 reconstituted extract, diluted additionally with 1/10 dH_2O, while B was diluted with 1/10 β-mercaptoethanol. Both were heated 10 min at 85 °C, centrifuged and measured. The black trace displays the BPC of the whole extract from fermentation supernatant.

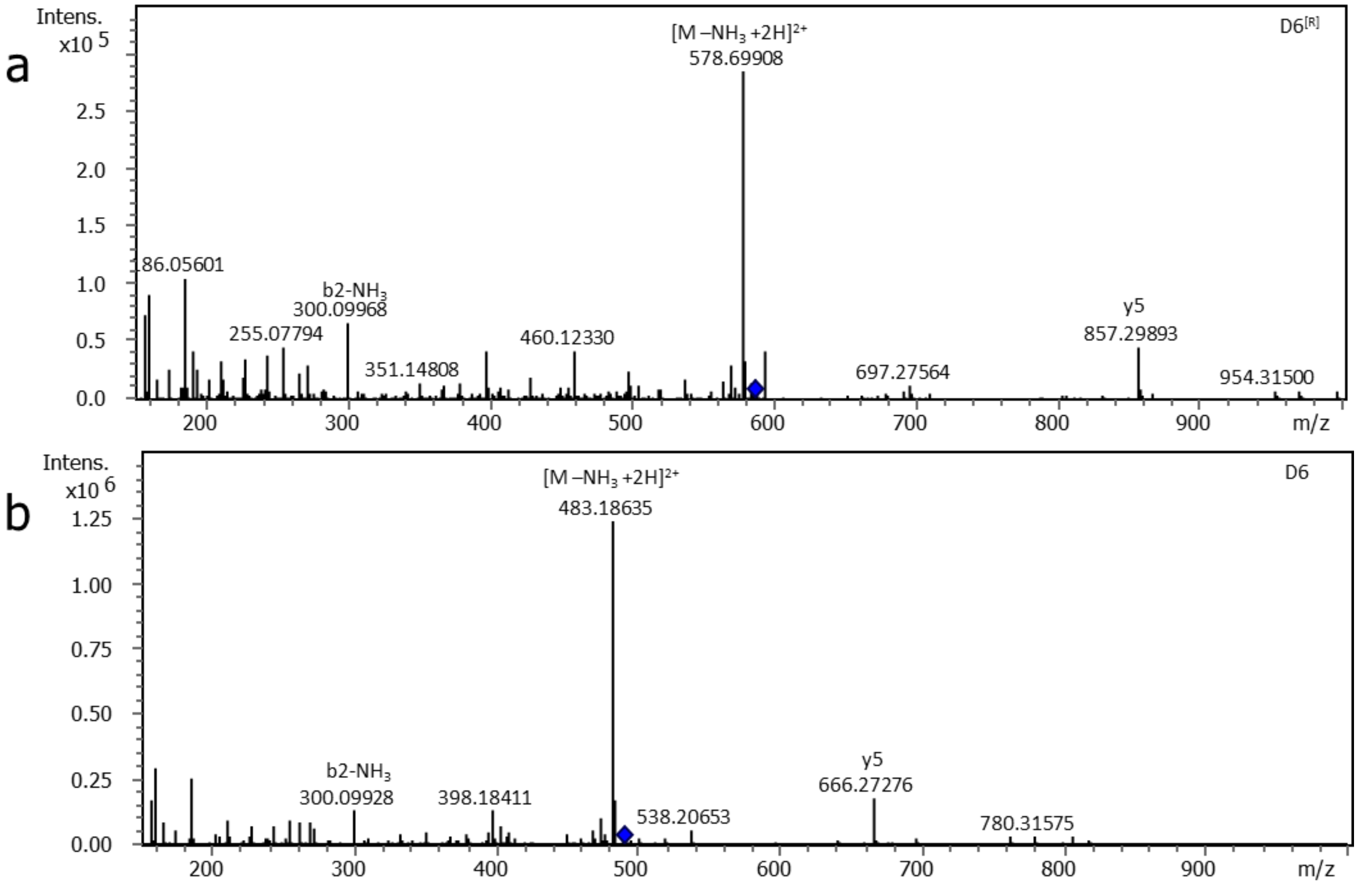

Figure S 34: MS2 spectrum of D6 before cleaving of with its most abundant protection group (a) and after reduction with β-mercaptoethanol (b).

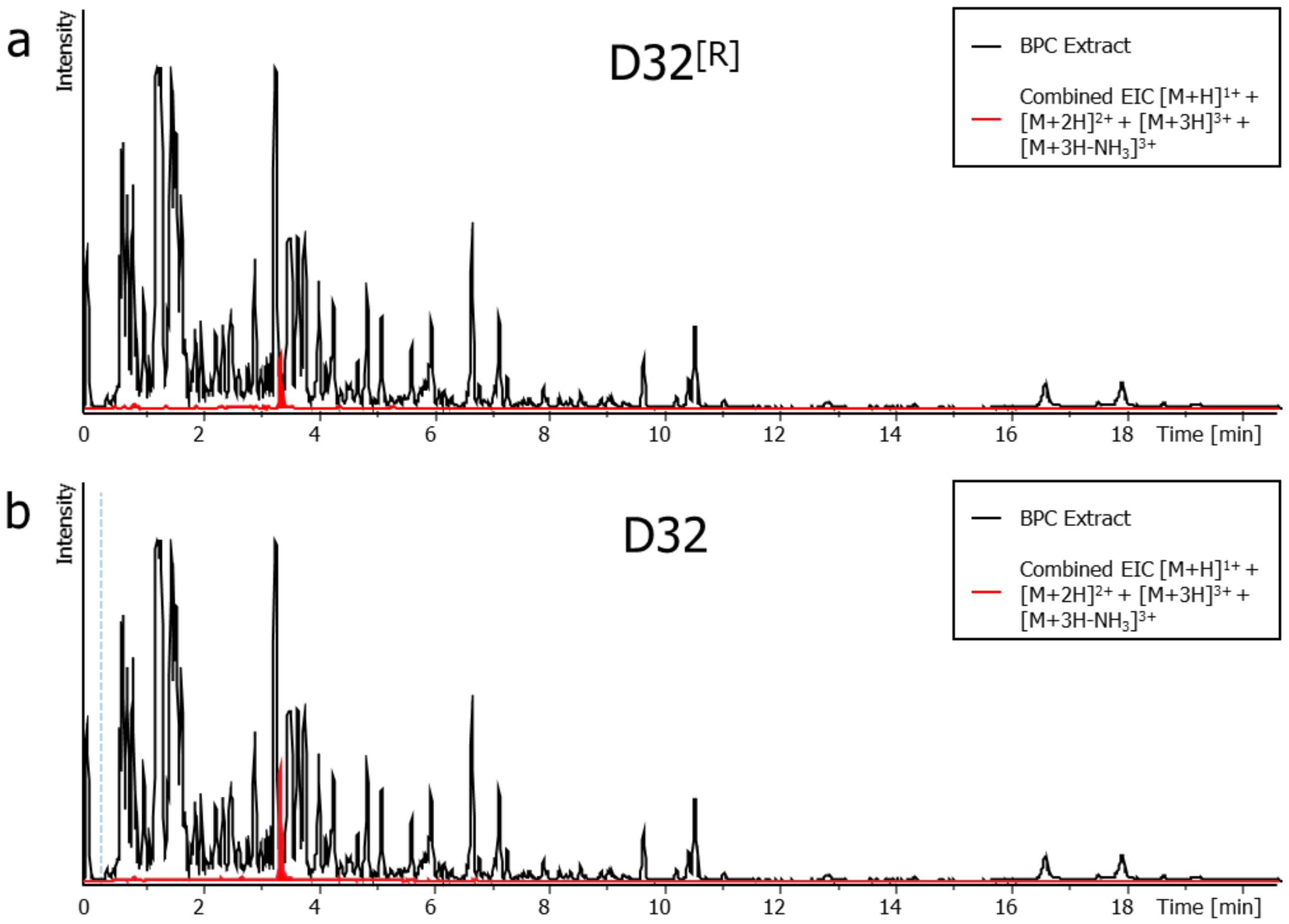

Figure S 35: Chromatogram of the *E. coli* BL21 (DE3) pNOSO-darABCDE-32 culture XAD16-N extract. The red trace displays the D32 combined EIC for the $[M+H]^{1+}$, $[M+2H]^{2+}$, $[M+3H]^{3+}$ and $[M+3H-NH_3]^{3+}$ species with expected protection group [R] on cysteine (a) and without protection group via reduction (b) at their respective calculated masses (Table S 3) ±0.02 Da. A displays the 1:5 reconstituted extract, diluted additionally with 1/10 dH_2O, while B was diluted with 1/10 β-mercaptoethanol. Both were heated 10 min at 85 °C, centrifuged and measured. The black trace displays the BPC of the whole extract from fermentation supernatant.

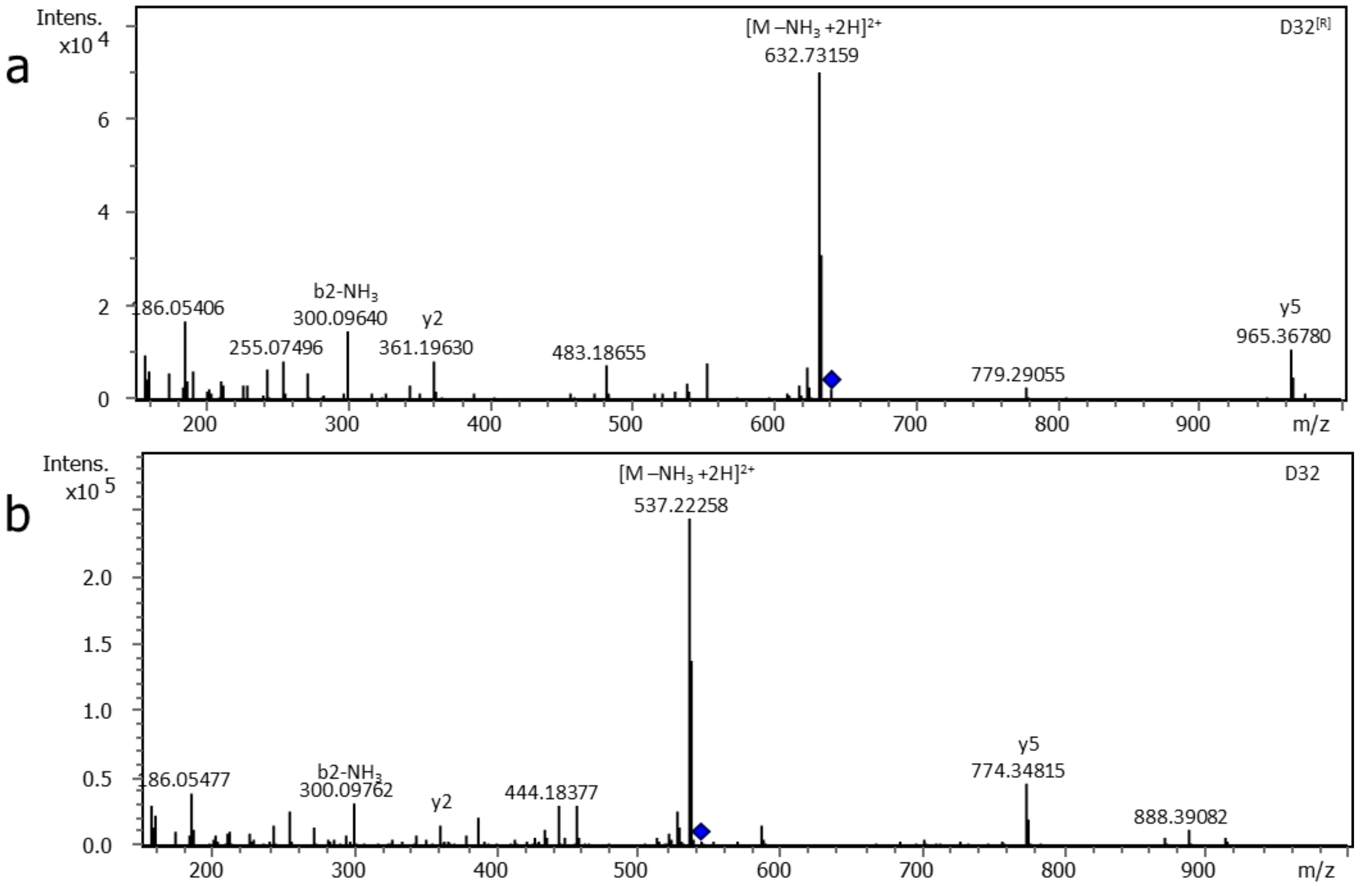

Figure S 36: MS2 spectrum of D32 before cleaving of with its most abundant protection group (a) and after reduction with β-mercaptoethanol (b).

Analyses of halogenated D9

MS and MS2 spectra

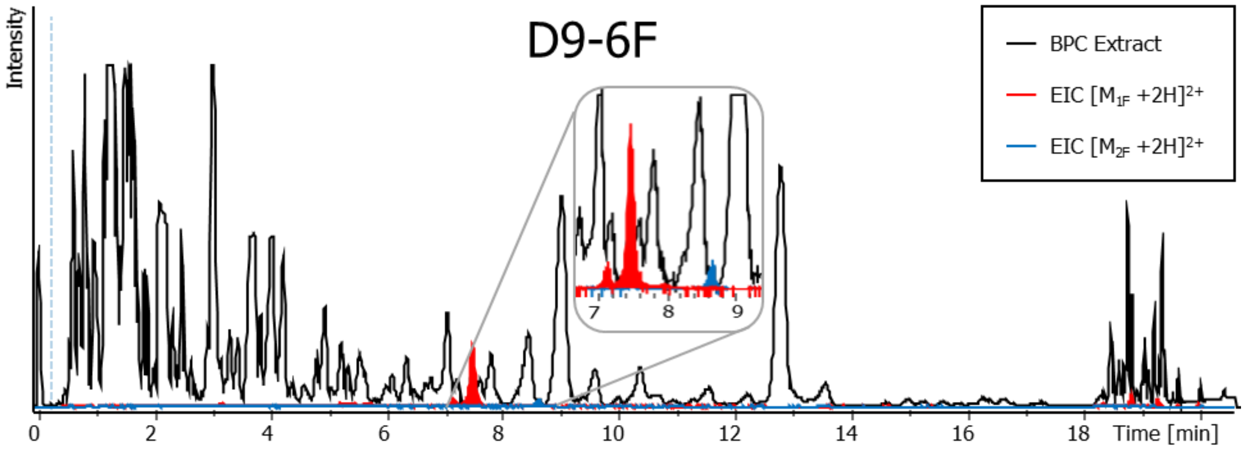

Figure S 37: Chromatogram of *E. coli* BL21 (DE3) pNOSO-darABCDE-9-6F culture XAD-16N extract. The red trace displays the two single fluorinated isomers and the blue trace the twofold fluorinated one at 7-9 min (grey: Zoom into the relevant area). Corresponding masses for fluorination on middle tryptophan (W3) differ, because of prevented ring formation between W3 and K5 and were not found with darobactin specific charge states.

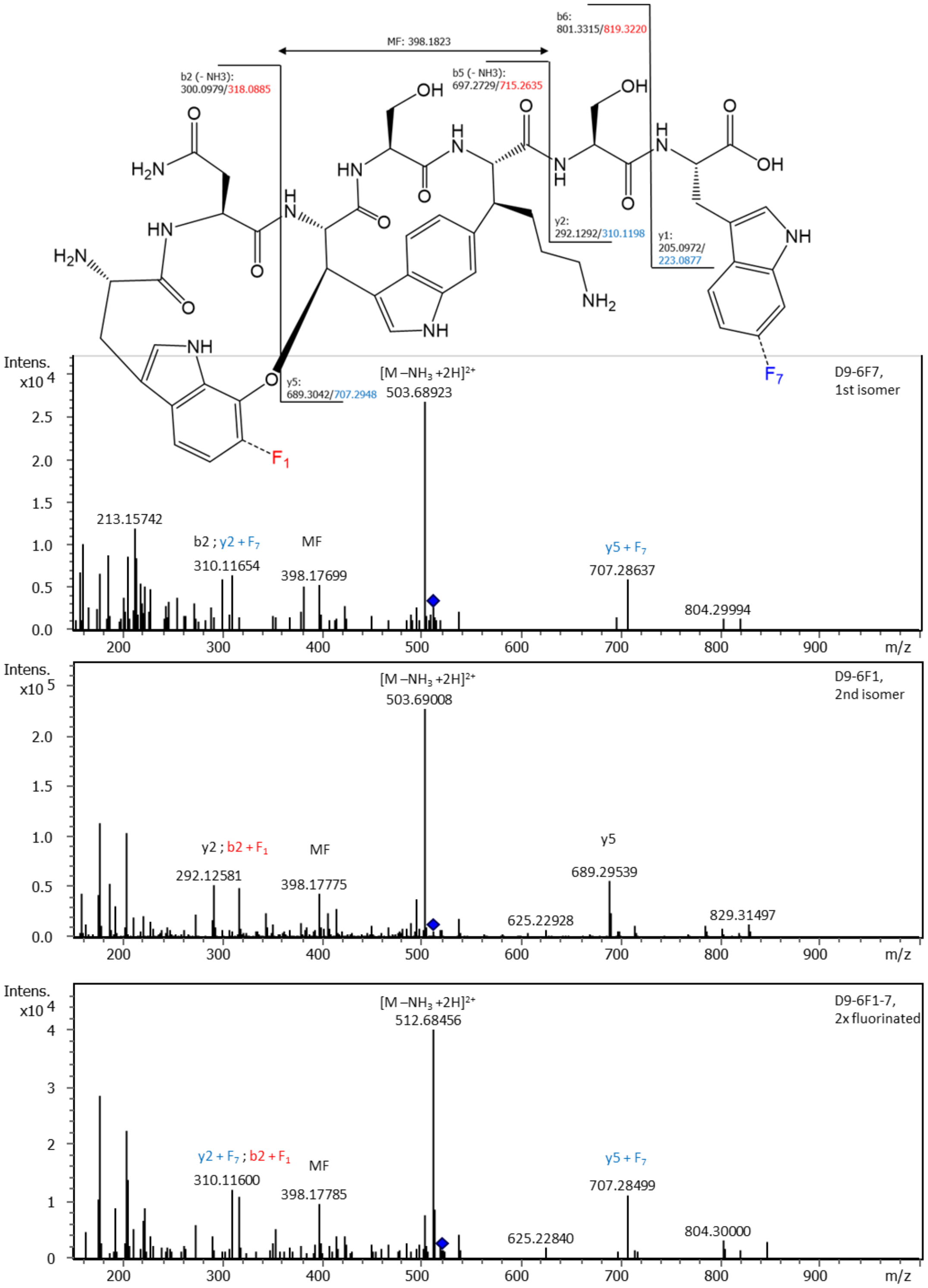

Figure S 38: MS2 fragmentation pattern of D9-6F. Possible fluorination sites from feeding 6-fluoro-L-tryptophan are highlighted in colors red (W1) and blue (W7). The colors are reflected in the potential mass shifts, which could be overserved of different b- and y- ions and (not of) middle fragment (MF). As fluorination on W3 would prevent the ring formation on this position its mass would differ by 2 hydrogens and was not observed. All ions can be assigned one of the two positions. Found mass shifts indicating the real position of fluorination are highlighted in their respective colors.

For fluorinated D9, fluorination on W3 would prevent ring formation, therefore differ in mass. The respective masses for fluorination on this position (and with other positions together) could not be found with a m/z of $[M+2H]^{2+}$ (513.2174, 522.2127, 531.2080). The other two

fluorinations are present and could be identified from their masses (Figure S 37) and MS2 fragmentation pattern (Figure S 38). First eluting, single fluorinated isomer (top) shows mass shifts of +17.99 (+F-H) on the y2 and y5 ion, therefore fluorination is only possible on last tryptophan (W7). Second eluting, single fluorinated isomer (middle) shows fluorine mass shift for b2-ion only, only possible for fluorination on W1. Two times fluorinated D9 (bottom) shows fluorine mass shifts of b2, y2 and y5 ions, so it is fluorinated on frontal and terminal tryptophans (W1,7). The main isomer (2nd eluting) was purified and was confirmed using NMR structure elucidation.

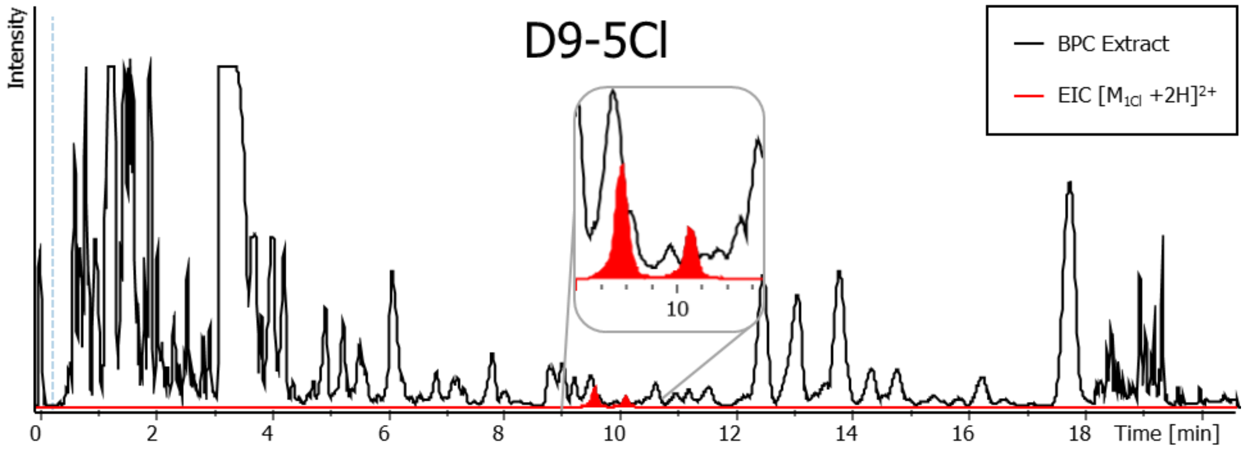

Figure S 39: Chromatogram of *E. coli* BL21 (DE3) pNOSO-darABCDE-9-5Cl culture XAD-16N extract. The red trace displays the two single chlorinated isomers between 9-10.2 min (grey: Zoom into the relevant area). Twofold and threefold chlorinated D9 could not be observed with darobactin specific charge state.

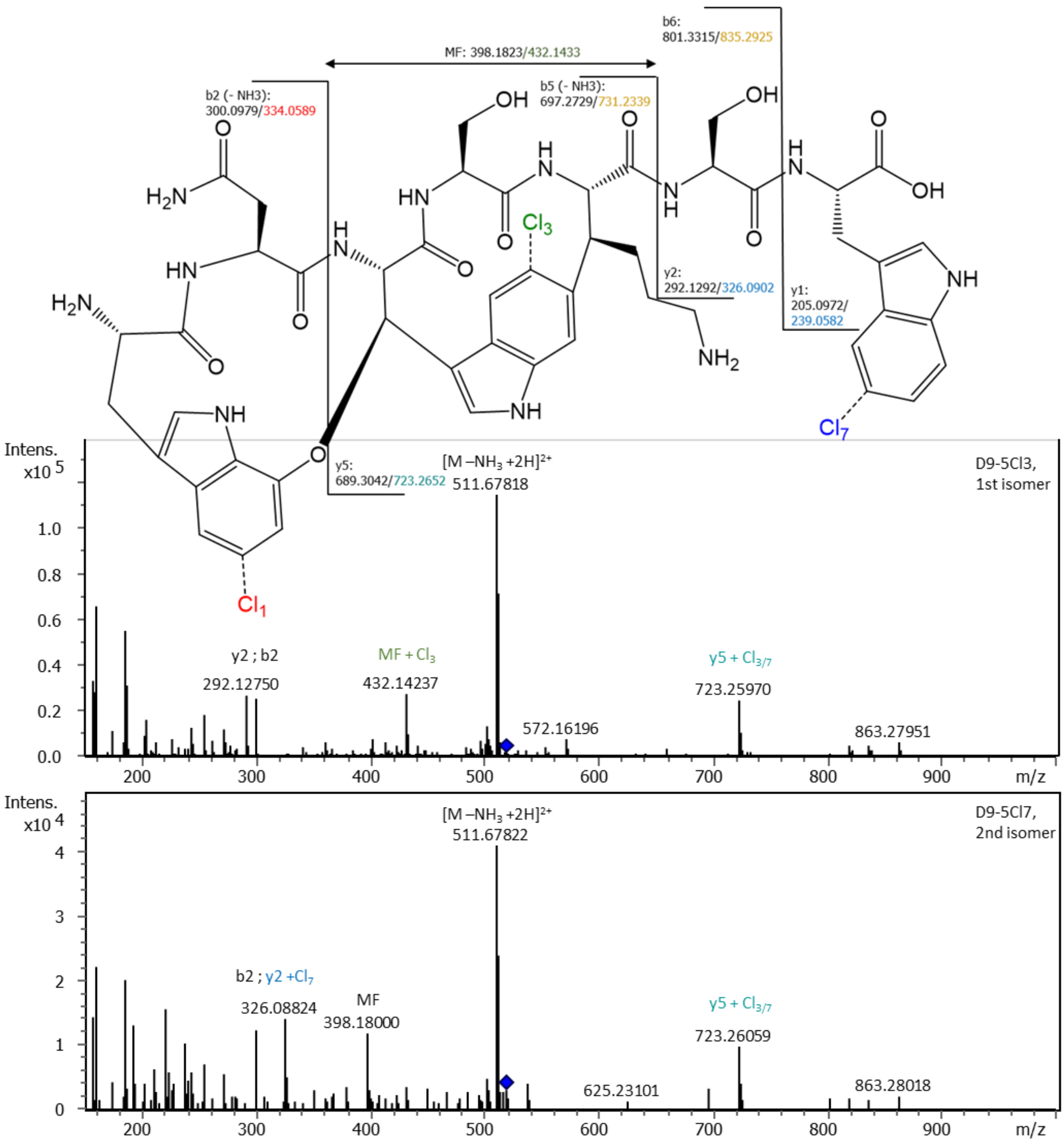

Figure S 40: MS2 fragmentation pattern of D9-5Cl. Possible chlorination sites from feeding 5-chloro-L-tryptophans are highlighted in colors red (W1), green (W3) and blue (W7). The colors are reflected in the potential mass shifts, which could be overserved of different b- and y- ions and middle fragment (MF). Found mass shifts indicating the real position of chlorination are highlighted in their respective colors. As some fragment masses indicate ambiguous chlorination sites, they are marked in the mixed colors of the two respective sites (blue + green equals turquoise, red + green equals orange).

For chlorinated D9, no two-fold (537.1753) or three-fold (554.1559) could be identified. However, two one-fold chlorinated isomers were observed (Figure S 39 and Figure S 40). The first eluting, single chlorinated isomer (top) shows a mass shift of +33.96 (+Cl-H) of middle fragment (MF) ion, and no mass shifts of y2- and b2-ions, therefore chlorination is only possible on second tryptophan (W3). Second eluting, single chlorinated isomer (bottom) shows chlorine mass shift for y2-ion only, as only possible for chlorination on W7. Two and three times chlorinated D9 could not be observed. While the chlorinated y5-ion fragment indicates

chlorination on either W3 or W7 (turquoise), based on the y2-ion and MF-ion, the position W3 for the first eluting isomer and the position W7 for the second isomer can be safely assumed.

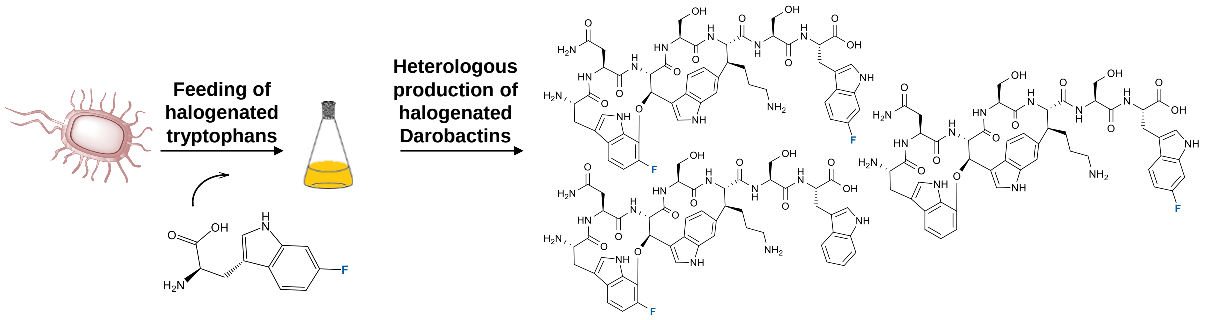

MIC [µg/mL]	*A. baumannii*		*E. coli*	*K. pneumoniae*	*P. aeruginosa*		
**Darobactin**	**DSM-30007**	**DSM-30008**	**ATCC25922**	**DSM-301204**	**DSM-22644**	**DSM-19882**	**PA14 Δ*mexAB***
**D9-6F1**	2 – 4	1	1	1	1	8	4
**D9-6F7**	2 - 4	1	0.5	0.5 - 1	0.5	8	4 - 8
**D9**	2	1	2	2	0.25	2	1
**DA**	16	2	2	2 - 4	0.5 - 1	16	4

Figure S 41: Generation and activity of fluorinated D9 against Gram-negative bacteria. *Acinetobacter baumannii* (*A. baumannii*), *Escherichia coli* (*E. coli*), *Klebsiella pneumoniae* (*K. pneumoniae*) and *Pseudomonas aeruginosa* (*P. aeruginosa*). Feeding of 6-fluoro-L-tryptophan to the production culture led to multiple halogenated D9 derivatives, where the fluorinated compound is incorporated into either the first (F1) or the terminal (F7) tryptophan. F1 and F7 are show approximately equipotent antibacterial activity to D9.

BamA interaction site

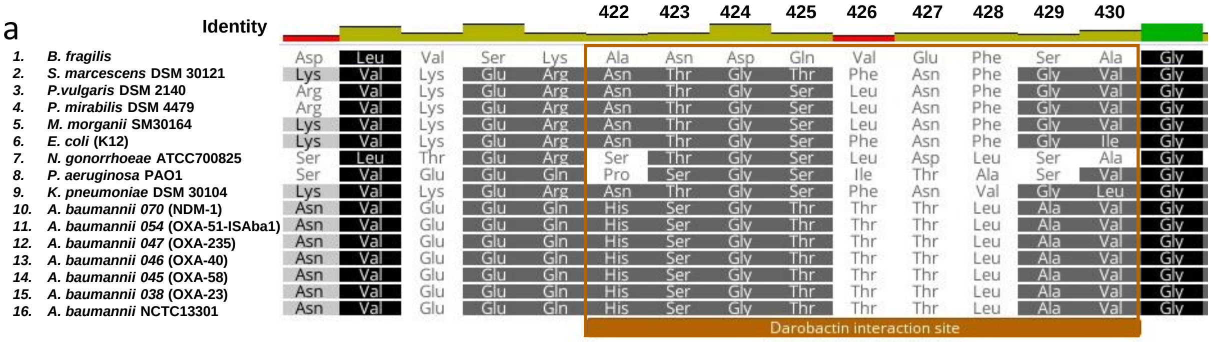

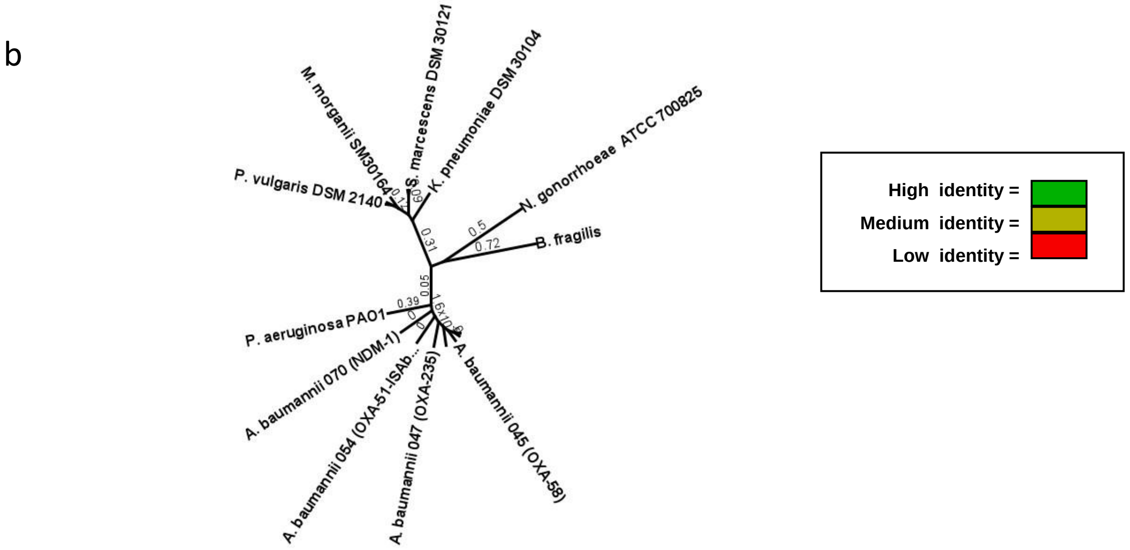

Figure S 42: Alignment of BamA sequence variations. a, BamA sequence alignment of, at least, one represent of each tested Gram-negative species (Table 2 and 3 of the manuscript) and of *Bacteroides fragilis* (*B. fragilis*) as control. b, phylogenetic tree of BamA amino acid sequences of selected bacteria. The color legend shows 100 % identity across all amino acid sequences in green, 30 % to below 100 % identity in yellow and less than 30 % in red. The graph was created with Geneious software 2021.2.2.

Cryo-EM of BAM-D22

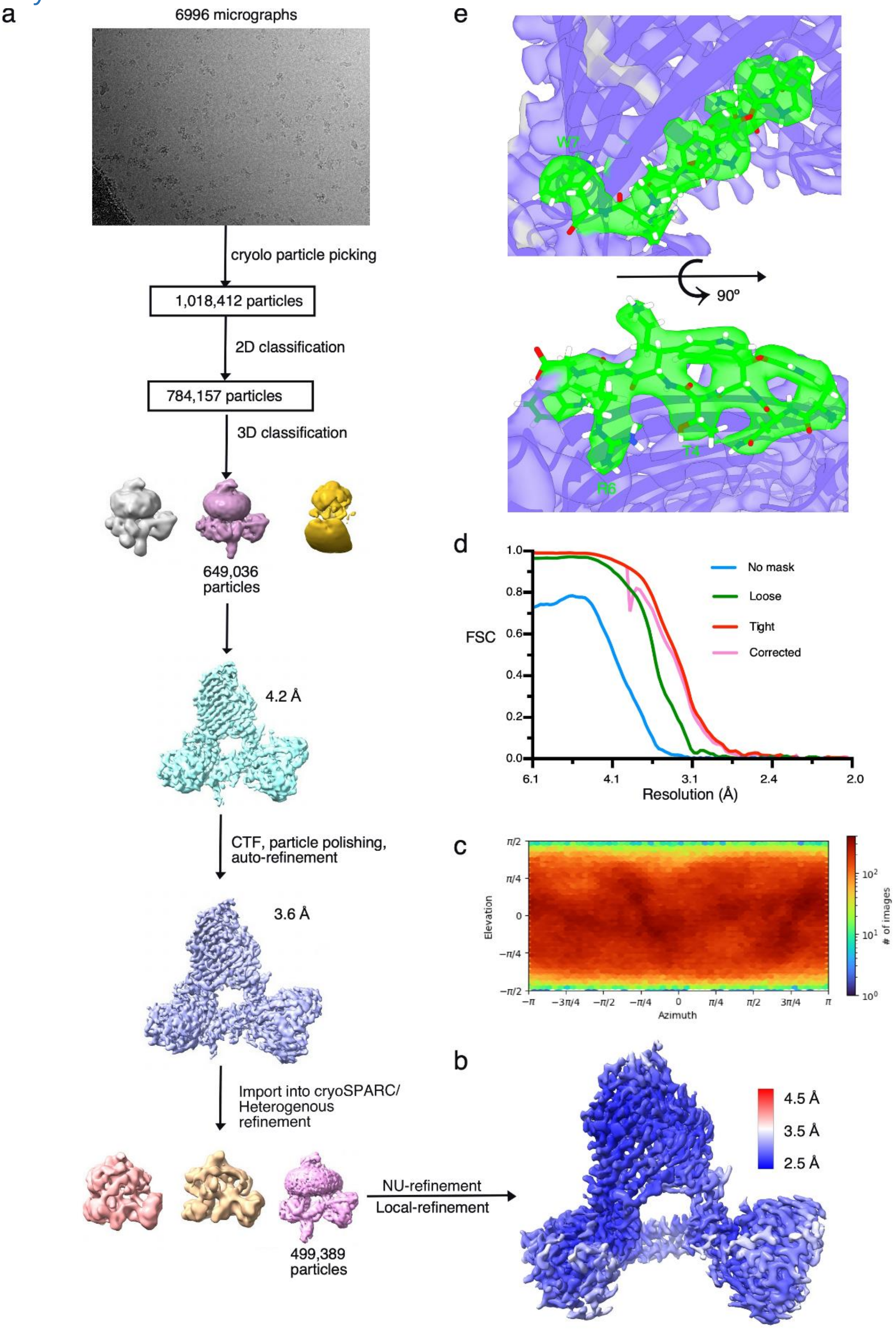

Figure S 43: Cryo-EM structure of BAM-D22 complex. a, workflow of data generation, processing and refinement to decipher BAM-D22 complex. b, BAM overview map with resolution of the EM reconstruction, highlighting local variations from 2.5 Å (blue) to 4.5 Å (red). c, angular distribution of D22 bound to BAM complex was calculated in cryoSPARC for particle projections. Heat map shows number of particles for each viewing angle. d, Fourier shell correlation (FSC) curves for corrected, tight, loose and unmasked masks. e, front (top) and 90 ° rotated (bottom) view of magnified BAM-D22 interaction area showing electron density surface of D22 (green) bound on BAM (blue). Modified positions of D22 compared to D9 were labeled (one letter code) as well as the terminal tryptophan (W_7).

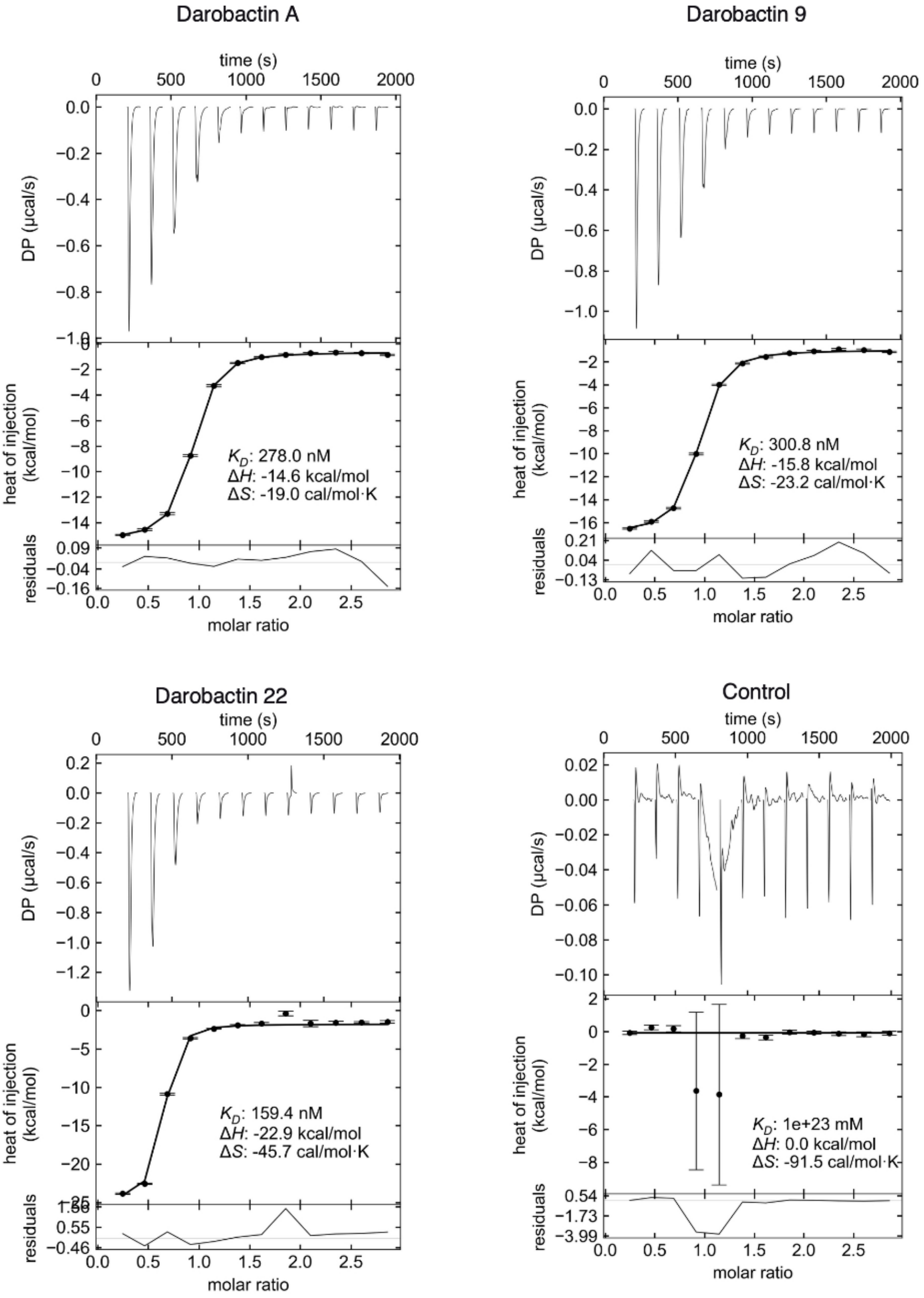

Figure S 44: Thermal titration calorimetry assays. Specific binding kinetics (K_D, Δ*H* and Δ*S*) of DA, D9 and D22 to *E. coli* BamA-β barrel were analyed and represented in the appropriate plot. Experiment was done in duplicates and repeated with similar results. Control assay was performed in absence of BamA-β barrel.

CRAB profiling

Antibiograms of clinical CRAB isolates

Table S 4: Antibiograms of clinical CRAB isolates. IMP: Imipenem; IMR: Imipenem/Relebactam; LEV: Levofloxacin; AMK: Amikacin; TOB: Tobramycin; MNO: Minocycline; TGC: Tigecyline; COL: Colistin

CRAB clinical isolate	MIC [µg/mL]							
	IMP	IMR	LEV	AMK	TOB	MNO	TGC	COL
038 (OXA-23)	32	32/4	8	16	32	1	2	0.5
045 (OXA-58)	8	8/4	8	128	1	4	1	0.5
046 (OXA-40)	64	64/4	8	>256	>128	4	1	0.5
047 (OXA-235)	8	8/4	8	>256	>128	16	1	0.5
070 (NDM-1)	32	64/4	4	>256	>128	≤0.0625	0.125	0.5
054 (OXA-51-ISAba1)	4	16/4	32	2	0.25	1	1	0.5

Table S 5: Activity assessment using 24 h population of TKC. Determining a MIC shift of *Acinetobacter baumannii* (*A. baumannii*) NCTC 13301 clones selected after a 24 h TKC experiment with high inoculum D22 at 2x, 4x and 8x MIC. No MIC shift was observed for the selected clones compared to the wild-type strain.

Activity assessment using 24 h population of TKC, MIC [µg/mL]	D22
A. baumannii NCTC 13301	8
A. baumannii NCTC 13301 (2x MIC)	8
A. baumannii NCTC 13301 (4x MIC)	8
A. baumannii NCTC 13301 (8x MIC)	8

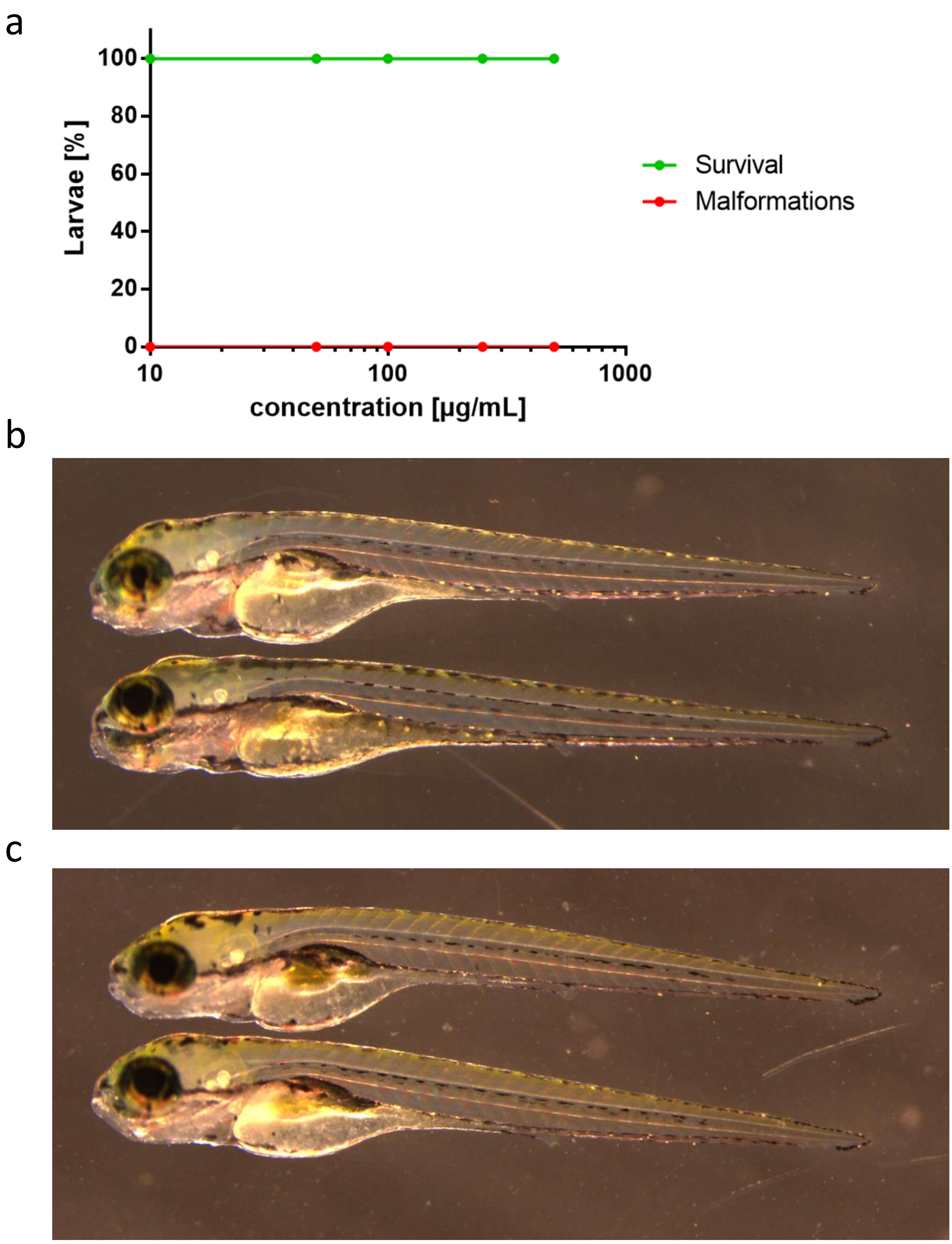

Figure S 45: No *in vivo* cytotoxicity against zebrafish larvae. D22 was added to surrounding fish water one day post fertilization (dpf) and left for four consecutive days (96 h) until five dpf. Tested concentrations were: 10, 50, 100, 250 and 500 µg/mL. Ten dechorinated larvae were tested per concentration. a, surviving rate : all zebrafish larvae survived. b, control zebrafish larvae. c, zebrafish larvae tested with 500 µg/mL. No malformation detected and no signs of toxicity detected.

Cryo-EM data collection, refinement, and validation statistics.

Table S 6: Cryo-EM data collection and refinement statistics and validation statistics of BAM-D9 and BAM-D22 data collection.

	BAM-D9 (EMDB-15363, PDB:8ADI)	BAM-D22 (EMDB-15362, PDB:8ADG)
	**Data collection and processing**	
**Microscope**	Titan Krios	Titan Krios
**Voltage (kV)**	300	300
**Detector**	K3	K3
**Magnification**	105,000	105,000
**Does per frame (e^{-}/Å^2)**	1.2	1.2
**Exposure rate (e^{-}/pixel/ s)**	15	15
**Calibrated pixel size (Å)**	0.85	0.85
**Defocus range (μm)**	–0.8 to –3	–0.6 to -3
**symmetry**	C1	C1
**Initial particles (no.)**	858,848	1,018,412
**Final particles (no.)**	406,142	499,389
**Map resolution (Å)**	3.4	3.0
**FSC threshold**	0.143	0.143
	**Refinement**	
**Initial model (PDB ID)**	7NRI	7NRI
**Model resolution (Å)**	3.5	3.1
**FSC threshold**	0.5	0.5
**Model composition**	5 chains, 1 ligand	5 chains, 1 ligand
**Non-hydrogen atoms**	11802	11,822
**Protein residues**	1499	1500
**Ligands**	D**9**	D**22**
	**B factors (Å^2)**	
**Protein**	7.35	42.32
**Ligand**	5.00	29.87
**Bond lengths (Å)**	0.003(0)	0.003 (0)
**Bond angles (°)**	0.538 (2)	0.557(3)
	**Validation**	
**MolProbity score**	1.44	1.12
**Clash score**	4.54	2.64
**Poor rotamers (%)**	0.0	0.00
	**Ramachandran plot**	
**Favored (%)**	96.7	96.9
**Allowed (%)**	3.3	3.1
**Disallowed (%)**	0.00	0.00

NMR confirmation of darobactins

D22

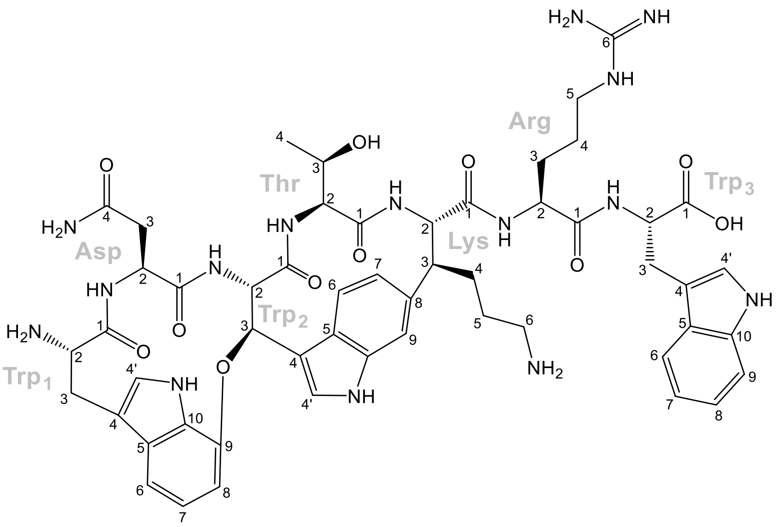

Table S 7: NMR spectroscopic data of D22.

position	δ ^{13}C	δ ^{1}H, mult (*J* in Hz)	COSY correlations	HMBC correlations
		NMR data in ACN/D_2O + 1% FA-d_4		
Trp3				
1	175.0	-	-	-
2	53.8	5.05, t (6.5)	3	1, 3, 4, *Arg*-1
3	26.7	3.70, m	2	1, 2, 3
4	109.1	-	-	-
4’	124.2	7.63, s	-	3, 4, 5, 9, 10
5	127.2	-	-	-
6	118.3	8.02, d (7.9)	7	8, 10
7	119.0	7.53, m	6, 8	5, 9
8	121.6	7.61, m	7, 9	6, 10
9	111.5	7.88, d (8.2)	8	5, 7
10	136.0	-	-	-
Arg				
1	172.0	-	-	-
2	53.0	4.66, dd (8.6, 5.6)	3	1, *Lys*-1, 3
3	27.9	1.93, 2.10, m	2, 4	1, 2, 4, 5
4	24.2	1.79, m	3, 5	3, 5
5	40.2	3.40, t (7.0)	4	3, 4, 6
6	156.4	-	-	-

NMR data in ACN/D_2O + 1% FA-d_4				
position	δ ^{13}C	δ ^{1}H, mult (*J* in Hz)	COSY correlations	HMBC correlations
Lys				
1	171.0	-	-	-
2	59.8	4.44, d (10.6)	3	1, 3, 4, *Thr*-1, *Trp₂*-8
3	47.7	3.29, dt (10.6, 3.0)	2, 4	1, 2, 4, 5, *Trp₂*-7, *Trp₃*-8, *Trp₂*-9
4	25.2	1.88, m	3, 5	3, 5, 6, *Trp₂*-8
5	25.3	2.13, m	4, 6	3, 4, 6
6	39.0	3.15, dq (11.6, 6.0)	5	4, 5
Thr				
1	167.5	-	-	-
2	57.7	4.08, d (6.3)	3	1, 3, 4, *Trp₂*-1
3	67.7	3.71, m	2, 4	2, 4
4	18.0	1.12, d (6.3)	3	2, 3
Trp₂				
1	167.7	-	-	-
2	63.2	4.99, d (8.9)	3	1, 3, 4, *Asp*-1
3	76.3	6.50, d (8.9)	2, 4‘	2, 4, 4‘, *Trp₃*-9
4	111.7	-	-	-
4‘	124.0	8.17, s	3	3, 4, 5, 10
5	124.6	-	-	-
6	117.0	7.74, d (8.3)	7	4, 8, 10
7	124.6	7.24, d (8.3)	6	5, 9, *Lys*-3
8	132.4	-	-	-
9	110.1	7.73, s	-	5, 7, *Lys*-3
10	136.8	-	-	-
Asp				
1	168.2	-	-	-
2	50.4	3.63, m	3	1, 3, 4, *Trp3*-1
3	38.6	2.49, d (6.8)	2	1, 2, 4
4	173.0	-	-	-
Trp₁				
1	168.0	-	-	-
2	54.5	4.33, dd (10.8, 7.2)	3	1, 3
3	26.0	3.61, 3.87, dd (13.4, 7.2)	2	1, 2, 4, 4‘, 5
4	107.9	-	-	-
4‘	124.7	7.68, s	-	3, 4, 5, 9, 10
5	128.9	-	-	-
6	119.8	7.52, m	7	4, 8, 10
7	113.1	7.53, m	6, 8	5, 9
8	108.12	7.53, m	7	6, 10
9	145.2	-	-	-
10	128.7	-	-	-

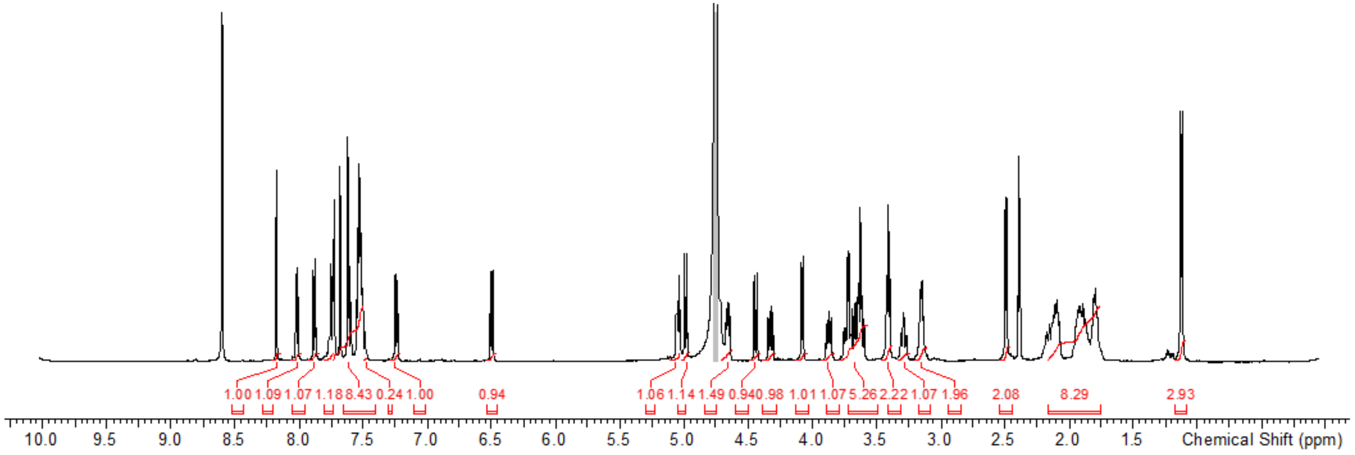

Figure S 46: ^{1}H spectrum of D22 in ACN/D2O + 1% FA at 45 °C and 500 MHz.

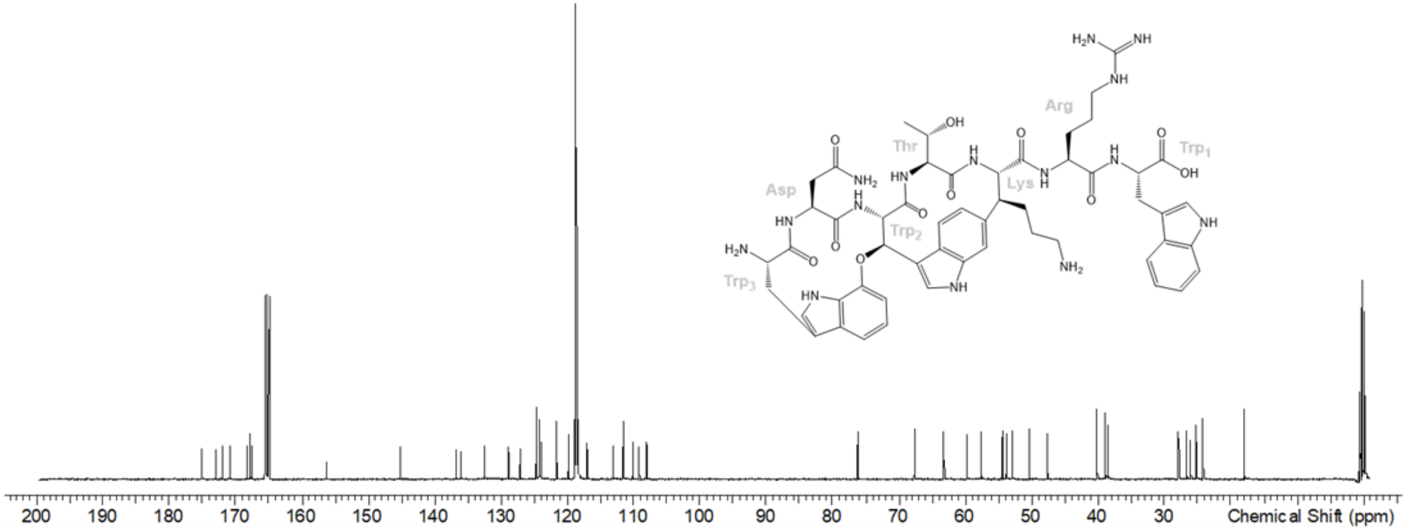

Figure S 47: ^{13}C spectrum of D22 in ACN/D2O + 1% FA at 45 °C and 125 MHz.

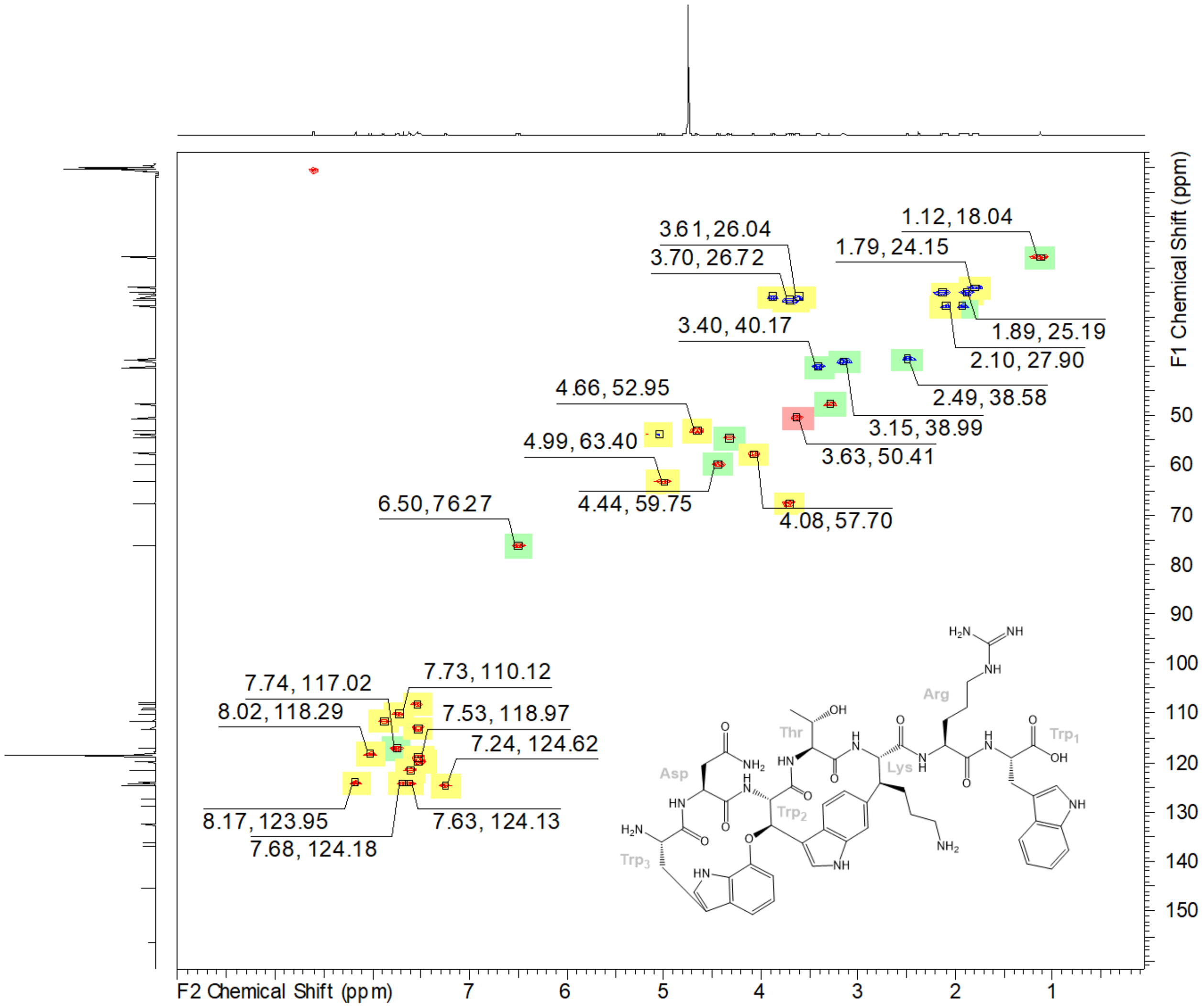

Figure S 48: HSQC spectrum of D22 in ACN/D2O + 1% FA at 45 °C and 500/125 MHz.

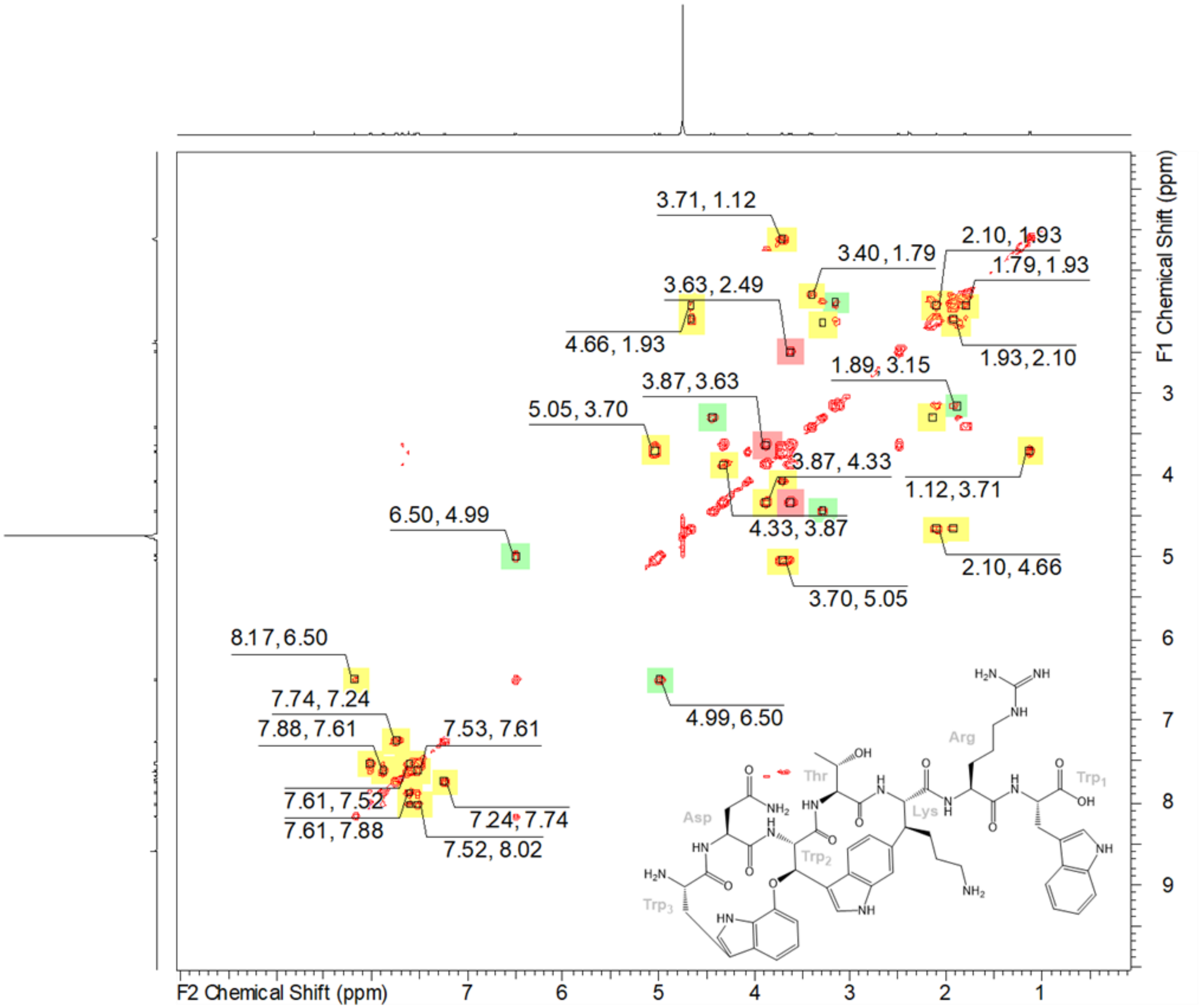

Figure S 49: COSY spectrum of D22 in ACN/D2O + 1% FA at 45 °C and 500/125 MHz.

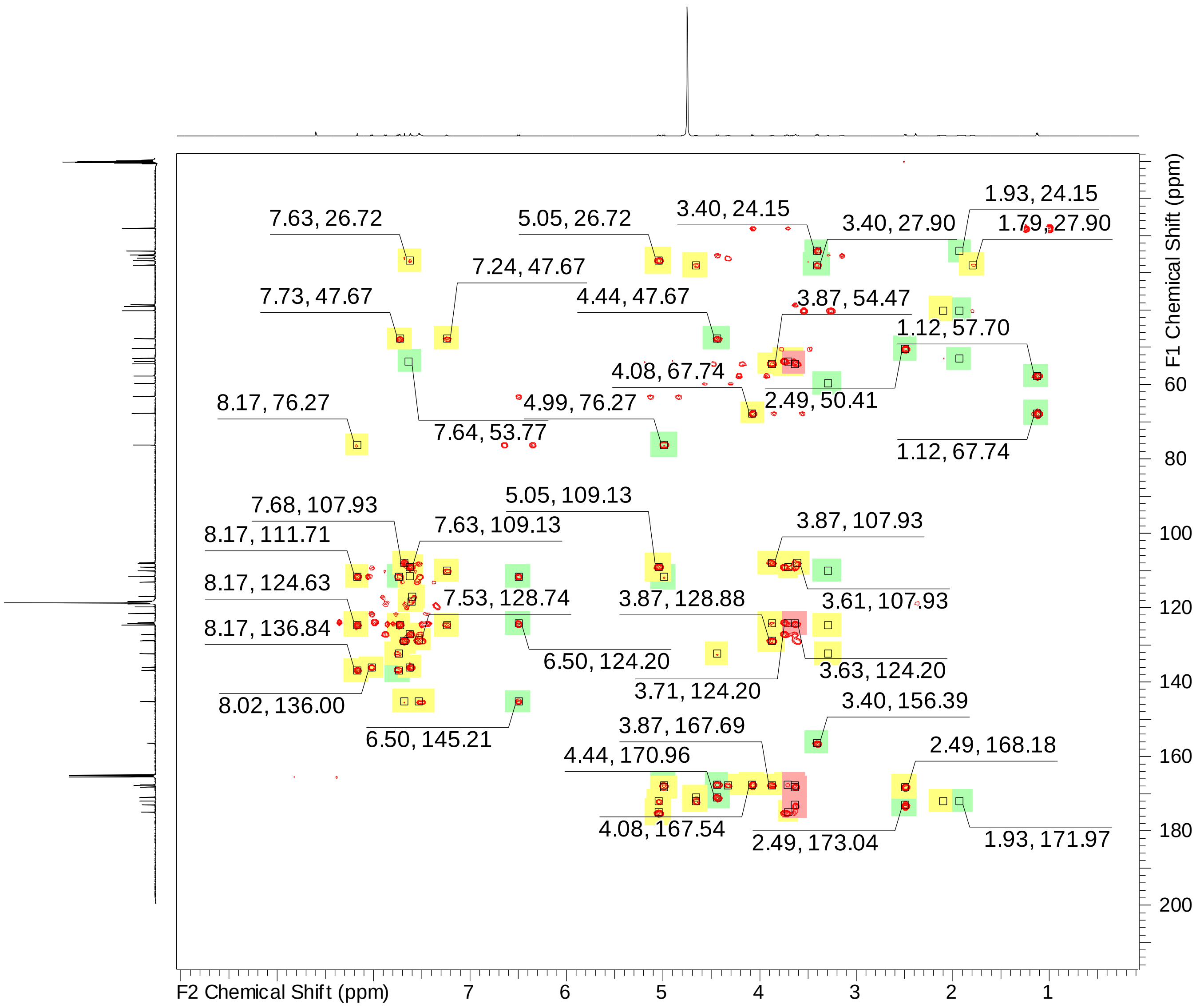

Figure S 50: HMBC spectrum of D22 in ACN/D2O + 1% FA at 45 °C and 500/125 MHz.

D23

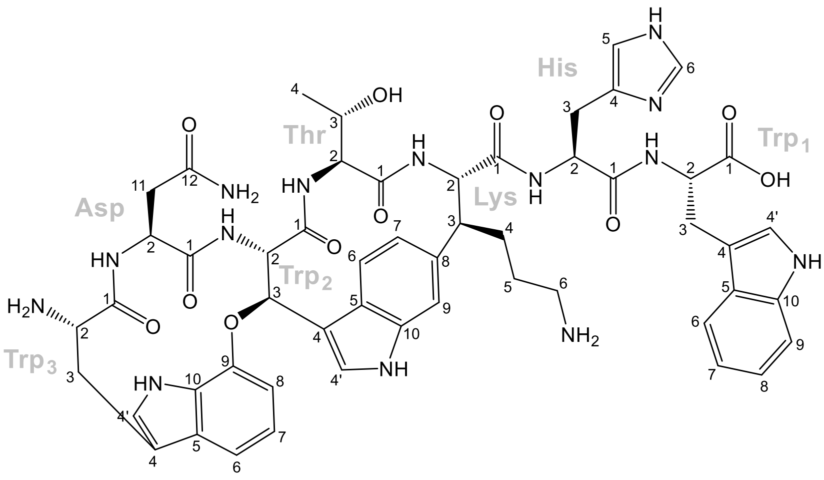

Table S 8: NMR spectroscopic data of D23.

		NMR data in ACN/D_2O + 1% FA-d_4		
position	δ ^{13}C	δ ^{1}H, mult (*J* in Hz)	COSY correlations	HMBC correlations
Trp₃				
1	176.3	-	-	-
2	55.4	5.04, m	3	1, 3, 4, *His*-1
3	27.9	3.71, m	2	1, 2, 3
				-
4	110.4	-	-	
				3, 4, 5, 9, 10
4’	125.4	7.62, s	-	
5	128.4	-	-	-
6	119.5	8.02, d (7.6)	7	8, 10
7	120.3	7.53, m	6, 8	5, 9
8	122.9	7.61, m	7, 9	6, 10
9	112.9	7.89, d (7.9)	8	5, 7
10	136.0	-	-	-
His				
1	171.5	-	-	-
2	53.1	5.05, m	3	1, *Lys*-1, 3, 4
3	27.4	3.39, 3.55, m	2	2, 4, 5
4	24.2	-	-	-
5	118.1	7.49, s	6	3, 4, 6
6	134.6	8.81, s	5	4, 5

		NMR data in ACN/D_2O + 1% FA-d_4		
position	δ ^{13}C	δ ^1H, mult (*J* in Hz)	COSY correlations	HMBC correlations
Lys				
1	171.2	-	-	-
2	60.9	4.41, d (9.8)	3	1, 3, 4, *Thr*-1, *Trp₂*-8
3	49.1	3.23, m	2, 4	1, 2, 4, 5, *Trp₂*-7, *Trp₃*-8, *Trp₂*-9
4	26.5	2.10, m	3, 5, 6	3, 5, 6, *Trp₂*-8
5	26.4	1.84, m	4, 6	3, 4, 6
6	40.2	3.10, m	4, 5	4, 5
Thr				
1	168.8	-	-	-
2	58.8	4.05, d (4.6)	3	1, 3, 4, *Trp₂*-1
3	69.2	3.67, m	2, 4	2, 4
4	19.3	1.04, d (4.8)	3	2, 3
Trp₂				
1	169.0	-	-	-
2	64.5	4.99, bd (8.2)	3	1, 3, 4, *Asp*-1
3	77.5	6.49, d (8.2)	2, 4‘	2, 4, 4‘, *Trp₃*-9
4	113.0	-	-	-
4‘	125.3	8.17, s	3	3, 4, 5, 10
5	125.9	-	-	-
6	118.3	7.74, d (7.7)	7	4, 8, 10
7	125.9	7.22, d (7.7)	6	5, 9, *Lys*-3
8	133.5	-	-	-
9	111.4	7.71, s	-	5, 7, *Lys*-3
10	138.1	-	-	-
Asp				
1	169.5	-	-	-
2	51.7	3.63, m	3	1, 3, 4, *Trp3*-1
3	39.8	2.49, d (6.4)	2	1, 2, 4
4	174.3	-	-	-
Trp₁				
1	169.0	-	-	-
2	55.7	4.33, m	3	1, 3
3	27.3	3.61, 3.87, dd (12.8, 6.0)	2	1, 2, 4, 4‘, 5
4	109.2	-	-	-
4‘	125.5	7.68, s	-	3, 4, 5, 9, 10
5	130.0	-	-	-
6	121.1	7.51, m	7	4, 8, 10
7	114.4	7.52, m	6, 8	5, 9
8	109.4	7.54, m	7	6, 10
9	146.5	-	-	-
10	130.1	-	-	-

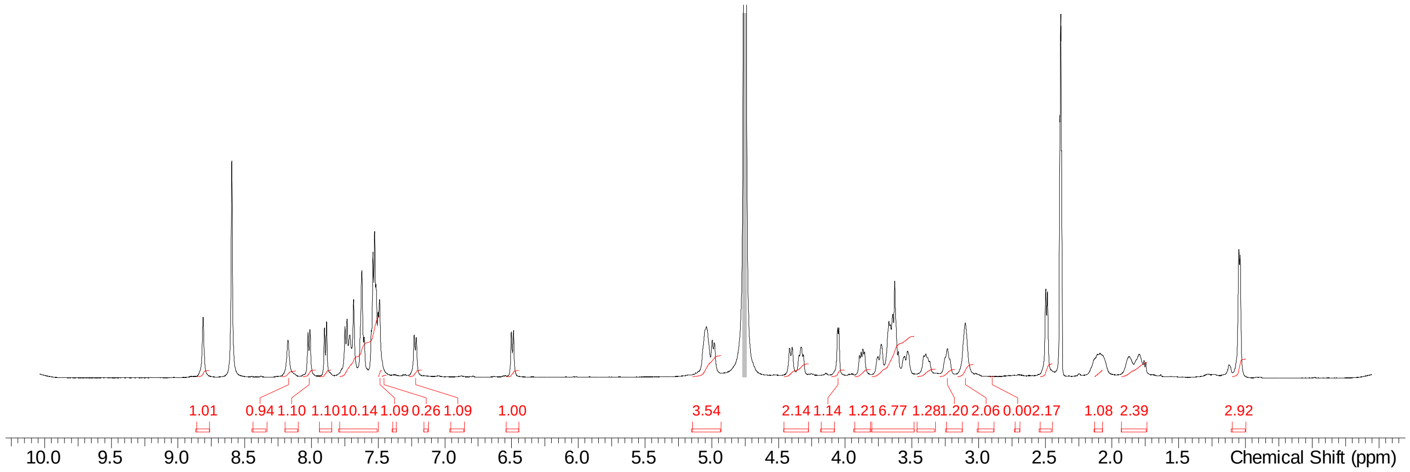

Figure S 51: ^{1}H spectrum of D23 in ACN/D2O + 1% FA at 45 °C and 500 MHz.

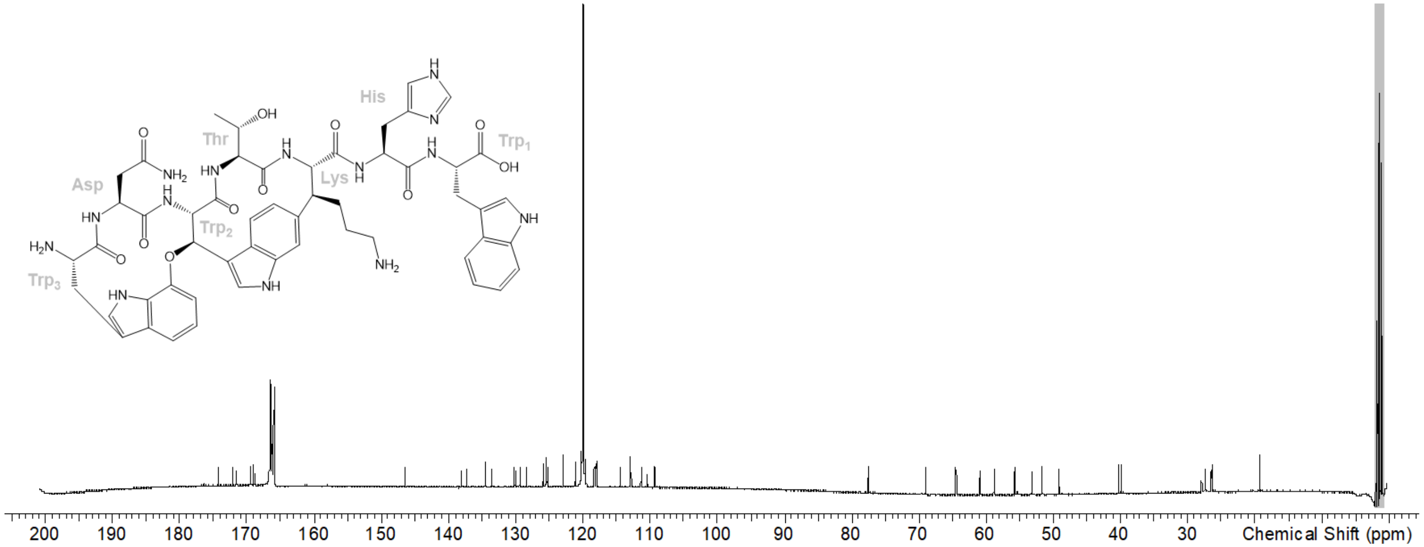

Figure S 52: ^{13}C spectrum of D23 in ACN/D2O + 1% FA at 45 °C and 125 MHz.

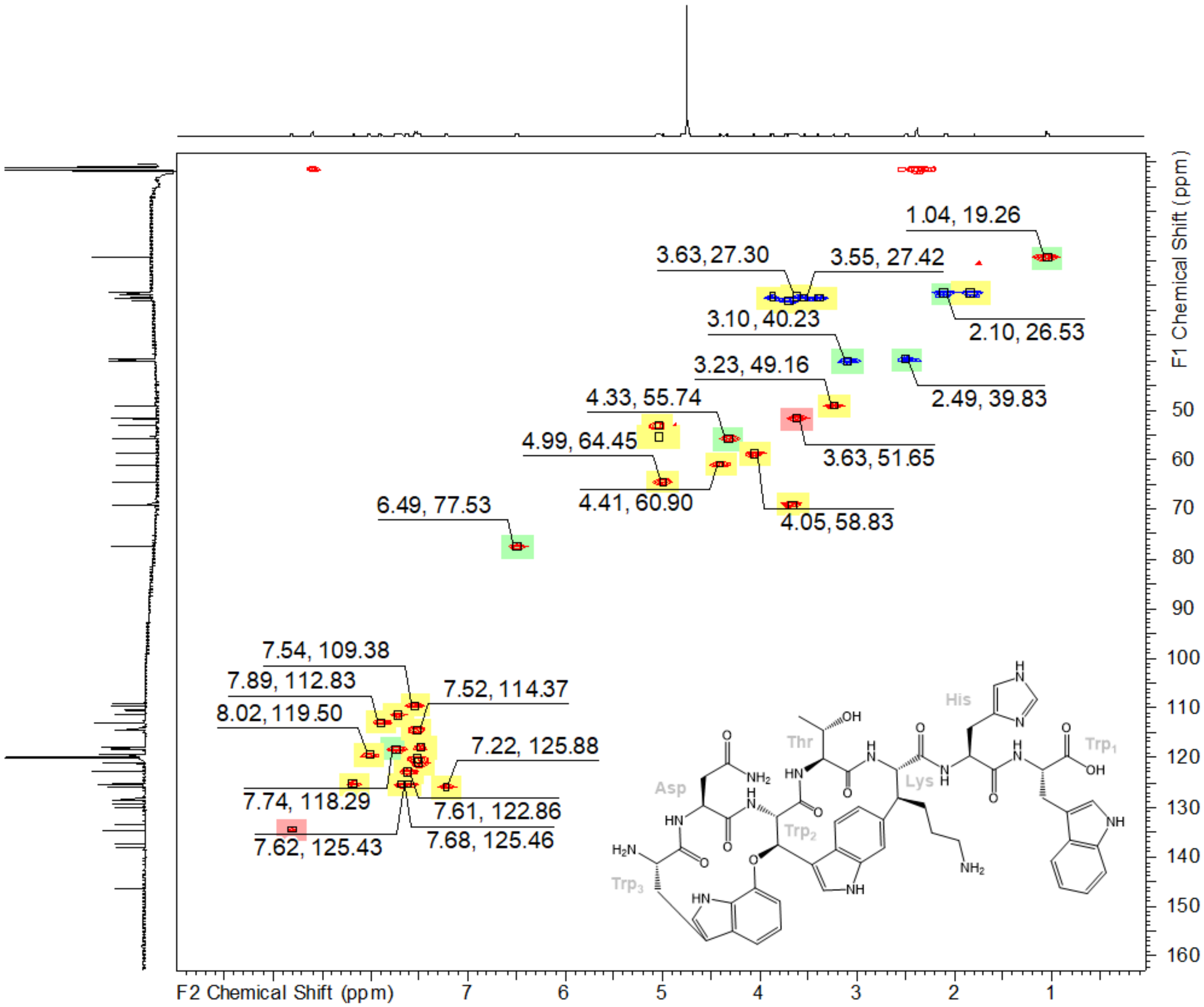

Figure S 53: HSQC spectrum of D23 in ACN/D2O + 1% FA at 45 °C and 500/125 MHz.

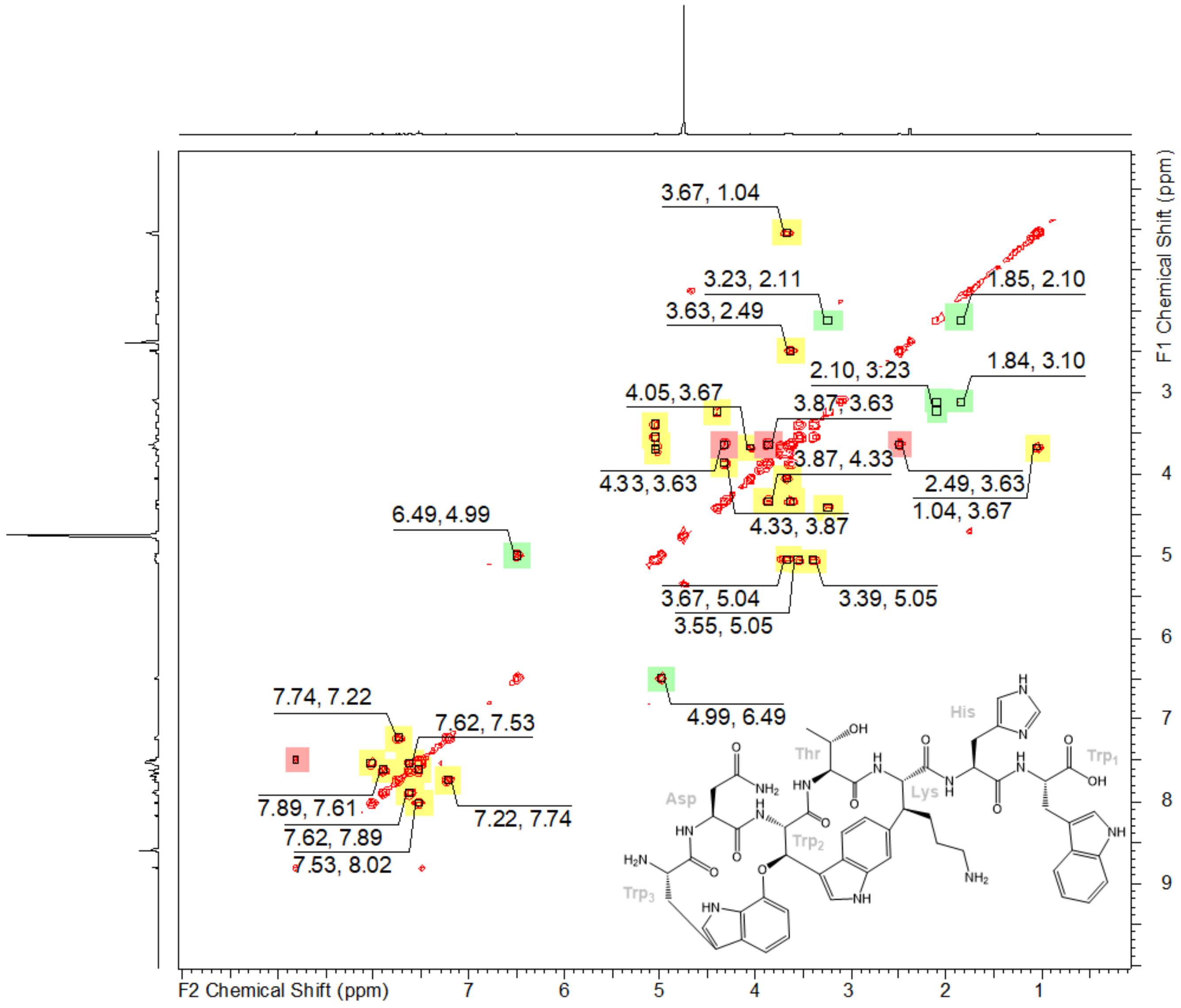

Figure S 54: COSY spectrum of D23 in ACN/D2O + 1% FA at 45 °C and 500/125 MHz.

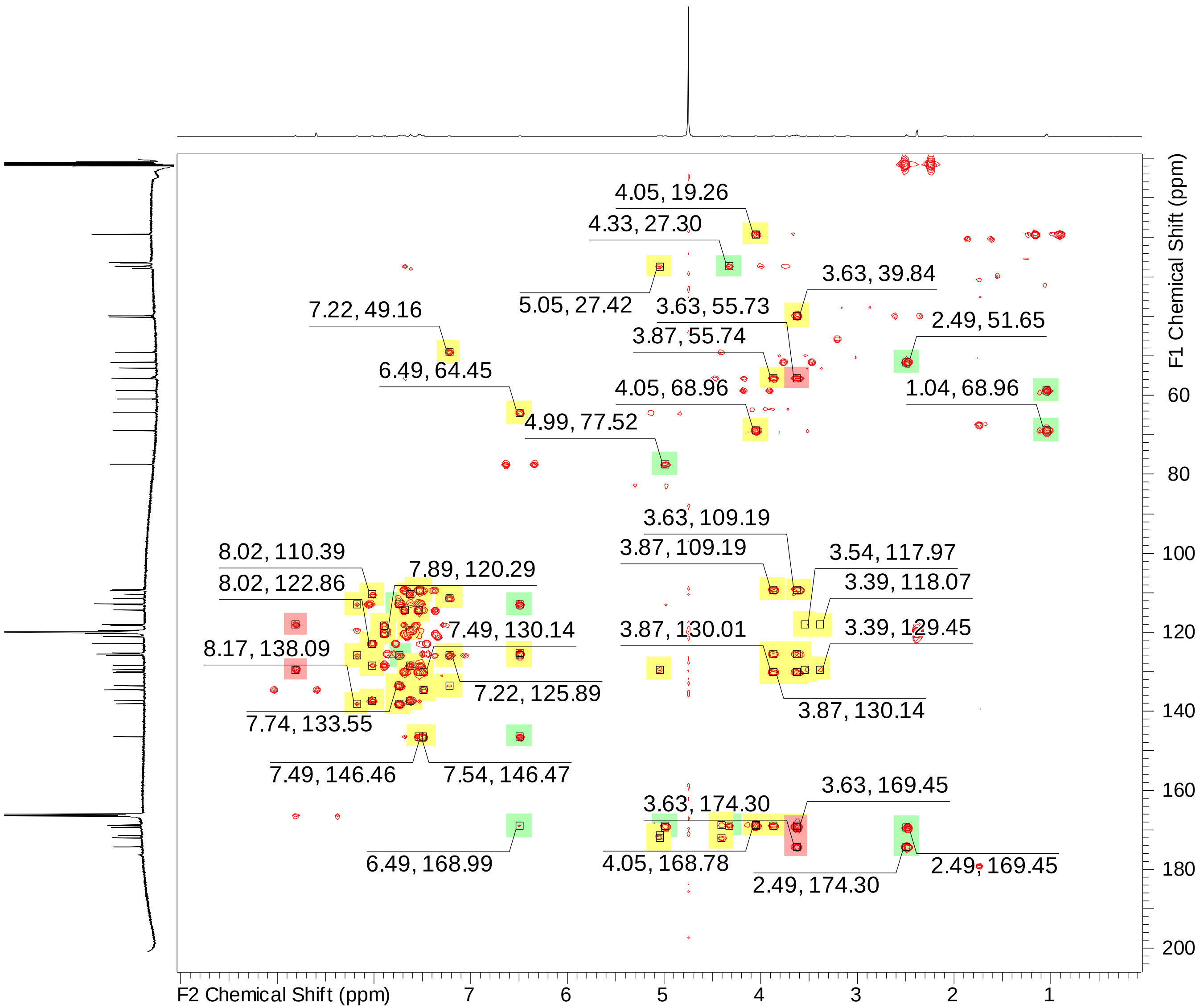

Figure S 55: HMBC spectrum of D23 in ACN/D2O + 1% FA at 45 °C and 500/125 MHz.

D31

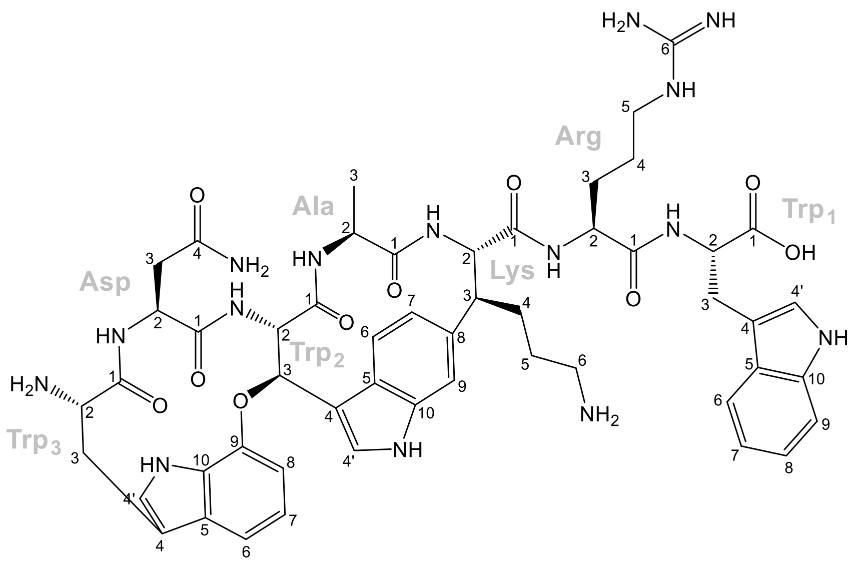

Table S 9: NMR spectroscopic data of D31.

		NMR data in ACN/D_2O + 1% FA-d_4		
position	δ ^{13}C	δ ^{1}H, mult (*J* in Hz)	COSY correlations	HMBC correlations
Trp$_3$				
1	175.0	-	-	-
2	53.9	5.16, bt (6.1)	3	1, 3, 4, *Arg*-1
3	26.9	3.77, 3.82, dd (14.6, 5.3)	2	1, 2, 3
4	109.4	-	-	-
4'	124.4	7.73, s	-	3, 4, 5, 9, 10
5	127.4	-	-	-
6	118.5	8.13, d (7.9)	7	8, 10
7	119.2	7.62, m	6, 8	5, 9
8	121.7	7.70, m	7, 9	6, 10
9	111.7	7.98, d (8.2)	8	5, 7
10	136.3	-	-	-
Arg				
1	172.2	-	-	-
2	53.3	4.75, q (6.9)	3	1, *Lys*-1, 3, 4
3	28.2	2.08, 2.22, m	2, 4	1, 2, 4, 5
4	24.4	1.93, m	3, 5	2, 3, 5
5	40.5	3.52, t (7.0)	3	3, 4, 6
6	156.7	-	-	-

position	δ ^{13}C	δ ^{1}H, mult (*J* in Hz)	COSY correlations	HMBC correlations
		NMR data in ACN/D_2O + 1% FA-d_4		
Lys				
1	171.2	-	-	-
2	60.1	4.53, d (10.5)	3	1, 3, 4, *Ala*-1, *Trp$_2$*-8
3	48.2	3.37, m	2, 4	1, 2, 4, 5, *Trp$_2$*-7, *Trp$_3$*-8, *Trp$_2$*-9
4	22.6	2.32, 2.03, m	3, 5, 6	3, 5, 6, *Trp$_2$*-8
5	25.4	2.23, m	4, 6	3, 4, 6
6	39.3	3.28, m	4, 5	4, 5
Ala				
1	170.9	-	-	-
2	48.1	4.36, q (6.9)	3	1, 3, *Trp$_2$*-1
3	18.4	1.26, d (6.9)	2	1, 2
Trp$_2$				
1	166.9	-	-	-
2	63.4	5.04, d (8.9)	3	1, 3, 4, *Asp*-1
3	76.3	6.58, d (8.9)	2, 4‘	2, 4, 4‘, *Trp$_3$*-9
4	112.1	-	-	-
4‘	124.2	8.28, s	3	3, 4, 5, 10
5	125.1	-	-	-
6	117.1	7.86, d (8.2)	7	4, 8, 10
7	125.0	7.35, d (8.2)	6	5, 9, *Lys*-3
8	132.6	-	-	-
9	110.4	7.84, s	-	5, 7, *Lys*-3
10	137.0	-	-	-
Asp				
1	168.3	-	-	-
2	50.7	3.72, m	3	1, 3, 4, *Trp3*-1
3	39.0	2.59, m	2	1, 2, 4
4	173.4	-	-	-
Trp$_1$				
1	167.9	-	-	-
2	54.8	4.43, dd (10.8, 7.2)	3	1, 3
3	26.3	3.97, 3.72, dd (13.5, 7.2)	2	1, 2, 4, 4‘, 5
4	108.2	-	-	-
4‘	124.4	7.79, s	-	3, 4, 5, 9, 10
5	128.9	-	-	-
6	120.1	7.61, m	7	4, 8, 10
7	113.2	7.62, m	6, 8	5, 9
8	108.0	7.62, m	7	6, 10
9	145.5	-	-	-
10	129.2	-	-	-

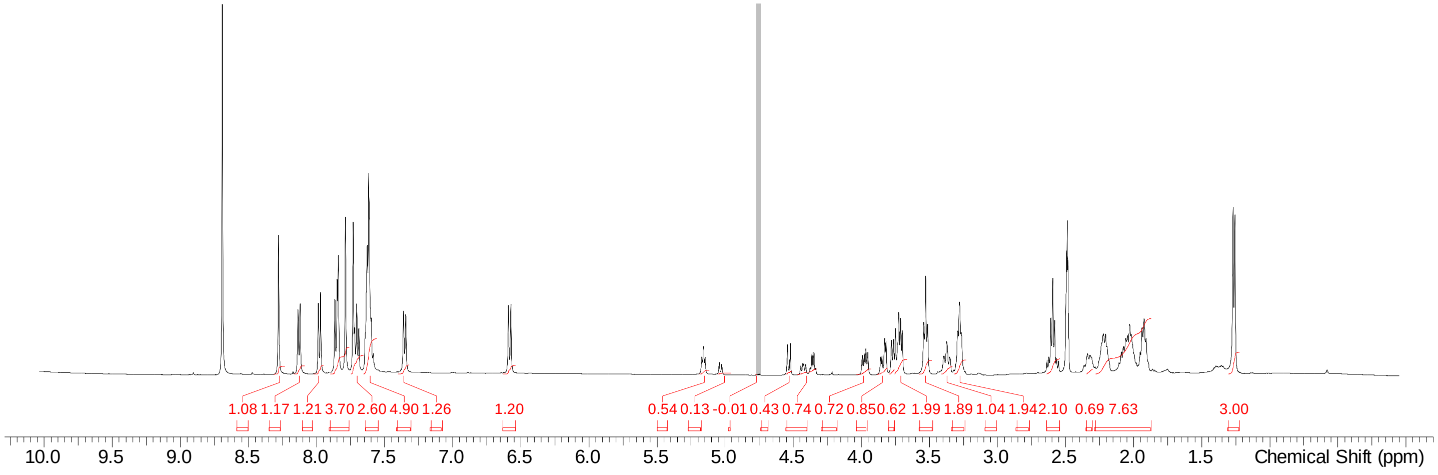

Figure S 56: Water suppressed ^{1}H spectrum of D31 in ACN/D2O + 1% FA at 45 °C and 500 MHz.

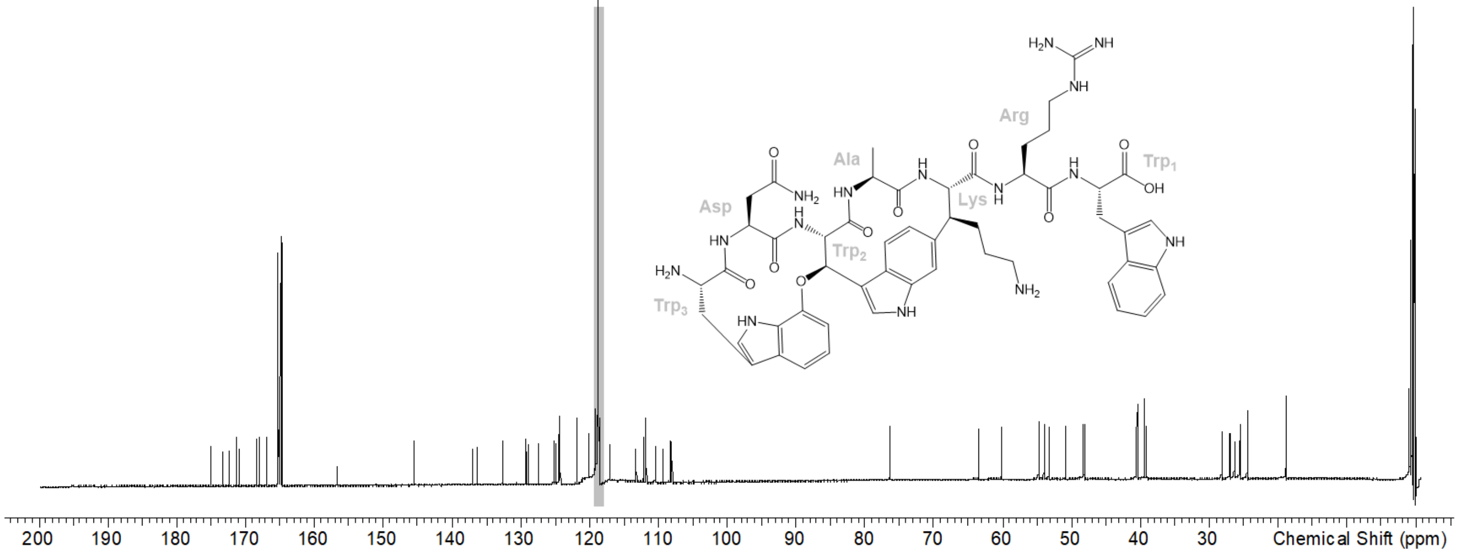

Figure S 57: ^{13}C spectrum of D31 in ACN/D2O + 1% FA at 45 °C and 125 MHz.

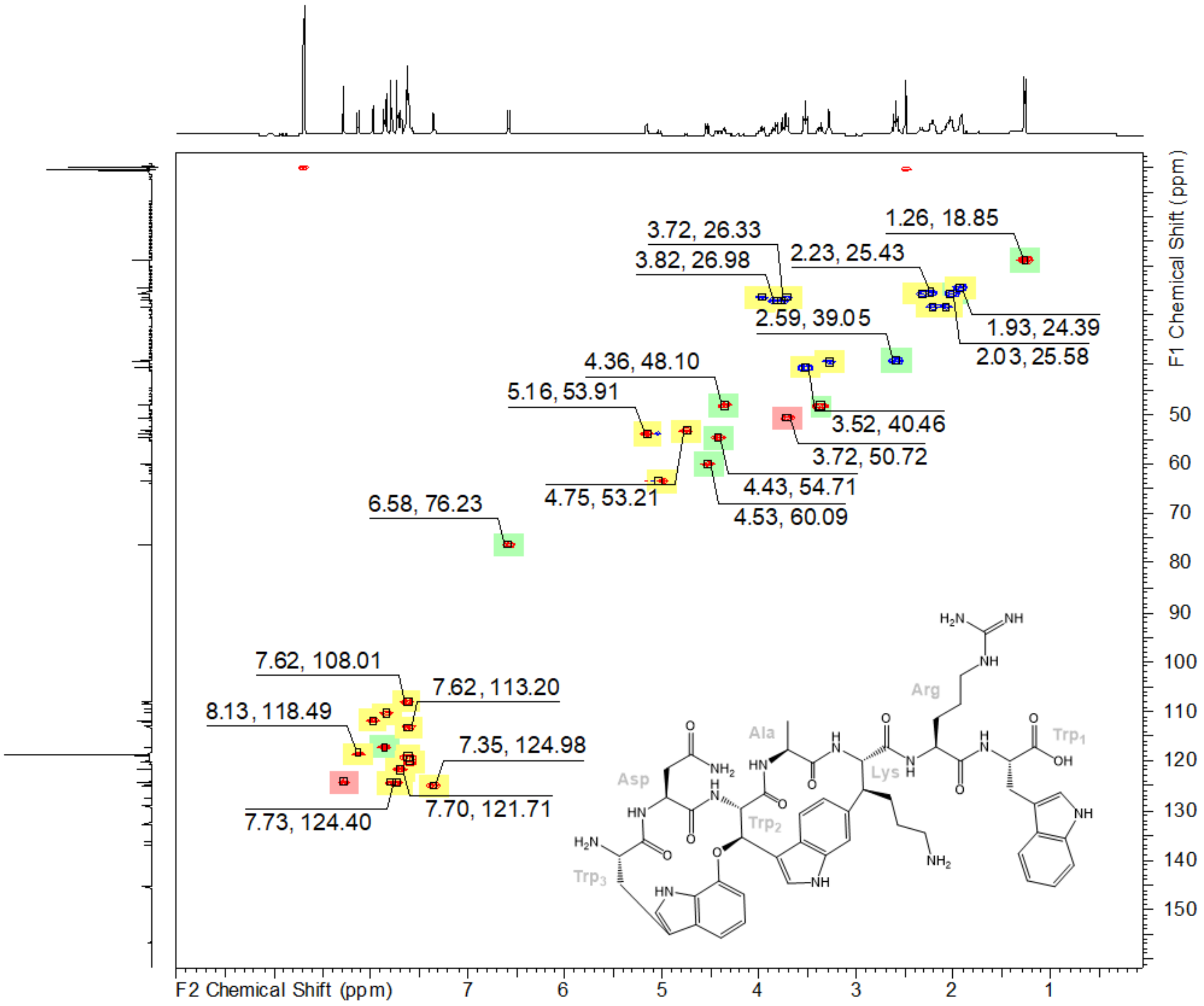

Figure S 58: HSQC spectrum of D31 in ACN/D2O + 1% FA at 45 °C and 500/125 MHz.

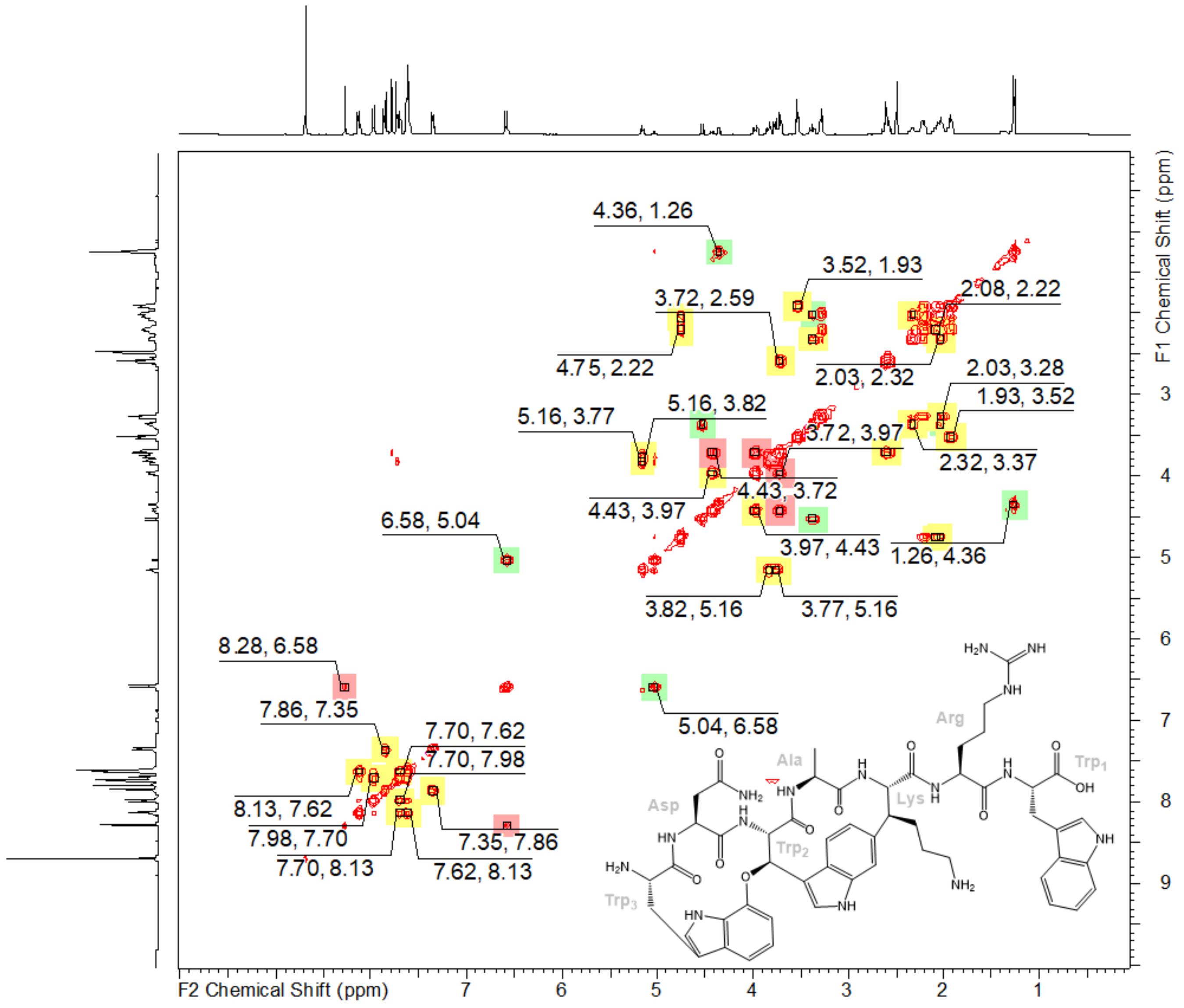

Figure S 59: COSY spectrum of D31 in ACN/D2O + 1% FA at 45 °C and 500/125 MHz.

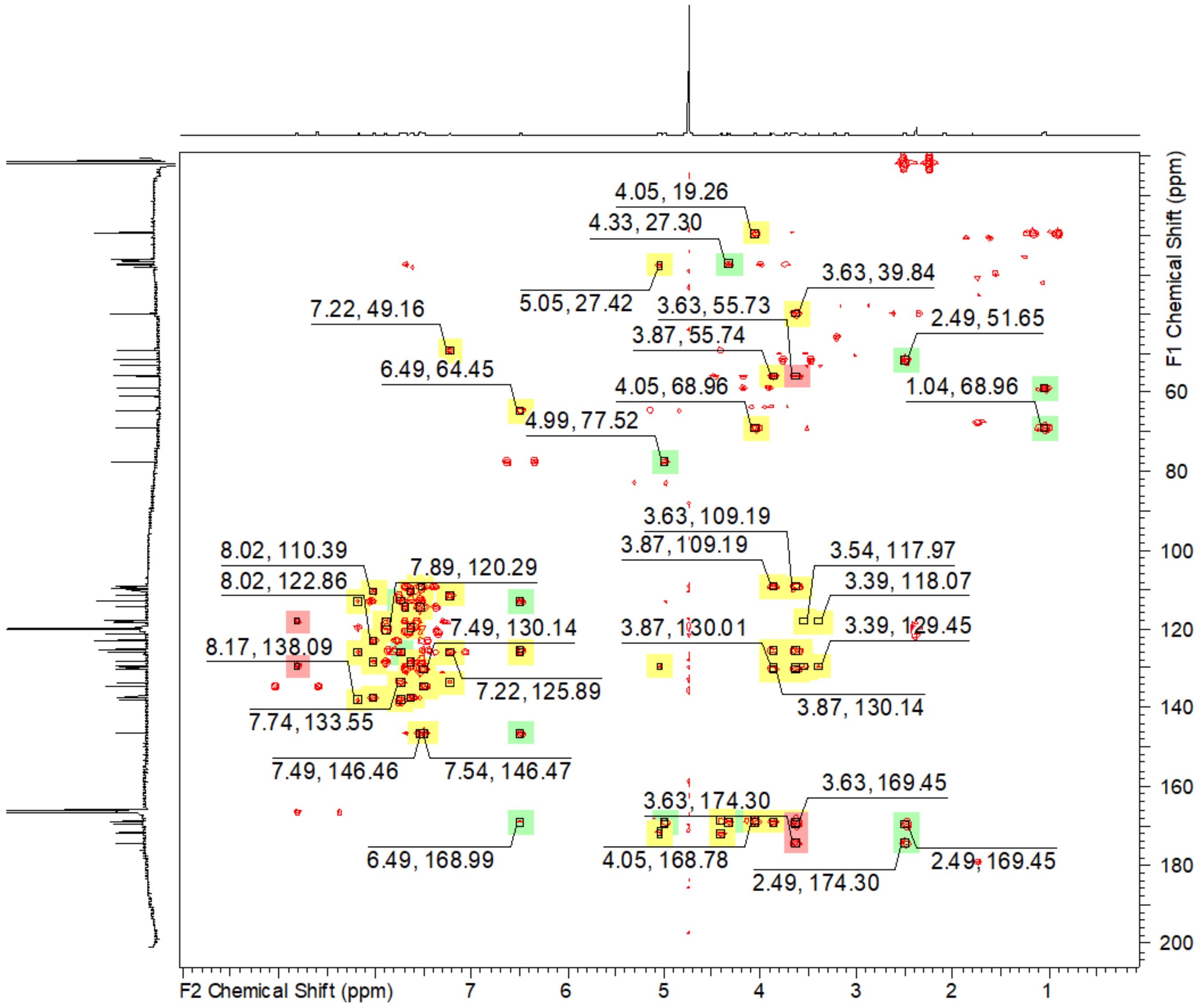

Figure S 60: HMBC spectrum of D31 in ACN/D2O + 1% FA at 45 °C and 500/125 MHz.

D36

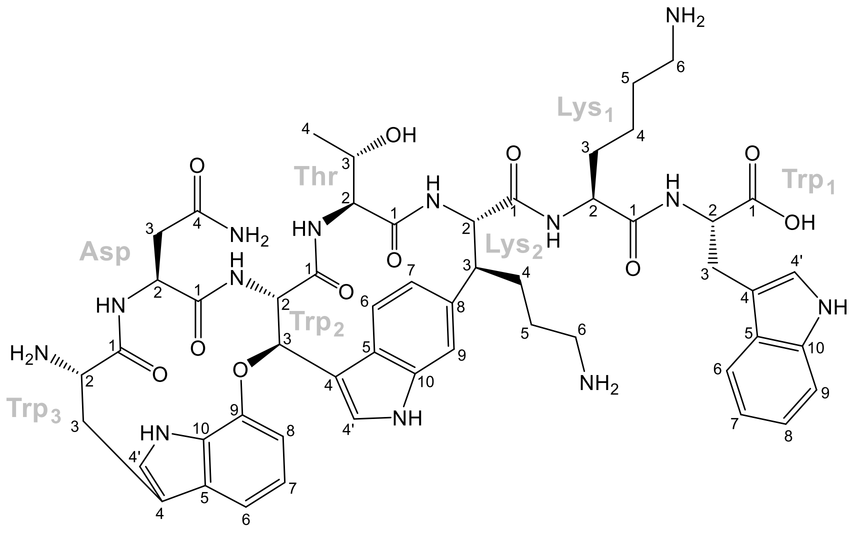

Table S 10: NMR spectroscopic data of D36.

	NMR data in ACN/D_2O + 1% FA-d_4			
position	δ ^{13}C	δ ^{1}H, mult (*J* in Hz)	COSY correlations	HMBC correlations
Trp₃				
1	175.0	-	-	-
2	53.7	5.03, bt (6.2)	3	1, 3, 4, *Lys₁*-1
3	26.7	3.66, m	2	1, 2, 3
4	109.1	-	-	-
4’	124.2	7.62, s	-	3, 4, 5, 9, 10
5	127.1	-	-	-
6	118.3	8.02, d (7.9)	7	8, 10
7	119.0	7.51, m	6, 8	5, 9
8	121.5	7.61, m	7, 9	6, 10
9	111.5	7.88, d (8.1)	8	5, 7
10	135.9	-	-	-
Lys₁				
1	172.2	-	-	-
2	53.1	4.62, dd (8.6, 5.7)	3	1, 3, 4, *Lys₂*-1
3	30.1	2.06, 1.91, m	2, 4	1, 2, 4
4	21.7	1.59, m	3, 5	2, 3, 5, 6
5	25.8	1.88, m	4, 6	2, 3, 6
6	38.8	3.17, t (7.7)	5	4, 5

position	δ ^{13}C	δ ^{1}H, mult (*J* in Hz)	COSY correlations	HMBC correlations
		NMR data in ACN/D_2O + 1% FA-d_4		
Lys_2				
1	170.9	-	-	-
2	59.7	4.42, d (10.5)	3	1, 3, 4, *Thr*-1, *Trp_2*-8
3	47.7	3.27, dt (10.9, 2.9)	2, 4	1, 2, 4, 5, *Trp_2*-7, *Trp_3*-8, *Trp_2*-9
4	25.1	1.85, m	3, 5	3, 5, 6, *Trp_2*-8
5	25.2	2.09, m	4, 6	3, 4, 6
6	38.9	3.12, m	5	4, 5
Thr				
1	167.5	-	-	-
2	57.6	4.06, d (6.1)	3	1, 3, 4, *Trp_2*-1
3	67.7	3.70, m	2, 4	2, 4
4	18.0	1.12, d (6.3)	3	2, 3
Trp_2				
1	167.6	-	-	-
2	63.2	4.98, d (8.7)	3	1, 3, 4, *Asp*-1
3	76.2	6.49, d (8.8)	2, 4‘	2, 4, 4‘, *Trp_3*-9
4	111.6	-	-	-
4‘	123.9	8.16, s	3	3, 4, 5, 10
5	124.	-	-	-
6	117.0	7.74, d (8.3)	7	4, 8, 10
7	124.6	7.24, d (8.2)	6	5, 9, *Lys_2*-3
8	132.4	-	-	-
9	110.1	7.73, s	-	5, 7, *Lys_2*-3
10	136.8	-	-	-
Asp				
1	168.1	-	-	-
2	50.4	3.61, m	3	1, 3, 4, *Trp3*-1
3	38.5	2.48, d (6.8)	2	1, 2, 4
4	173.0	-	-	-
Trp_1				
1	167.7	-	-	-
2	54.4	4.32, dd (10.8, 7.2)	3	1, 3
3	26.0	3.61, 3.86, dd (13.4, 7.1)	2	1, 2, 4, 4‘, 5
4	107.9	-	-	-
4‘	124.1	7.67, s	-	3, 4, 5, 9, 10
5	128.7	-	-	-
6	119.8	7.49, m	7	4, 8, 10
7	113.1	7.52, m	6, 8	5, 9
8	108.1	7.52, m	7	6, 10
9	145.1	-	-	-
10	128.8	-	-	-

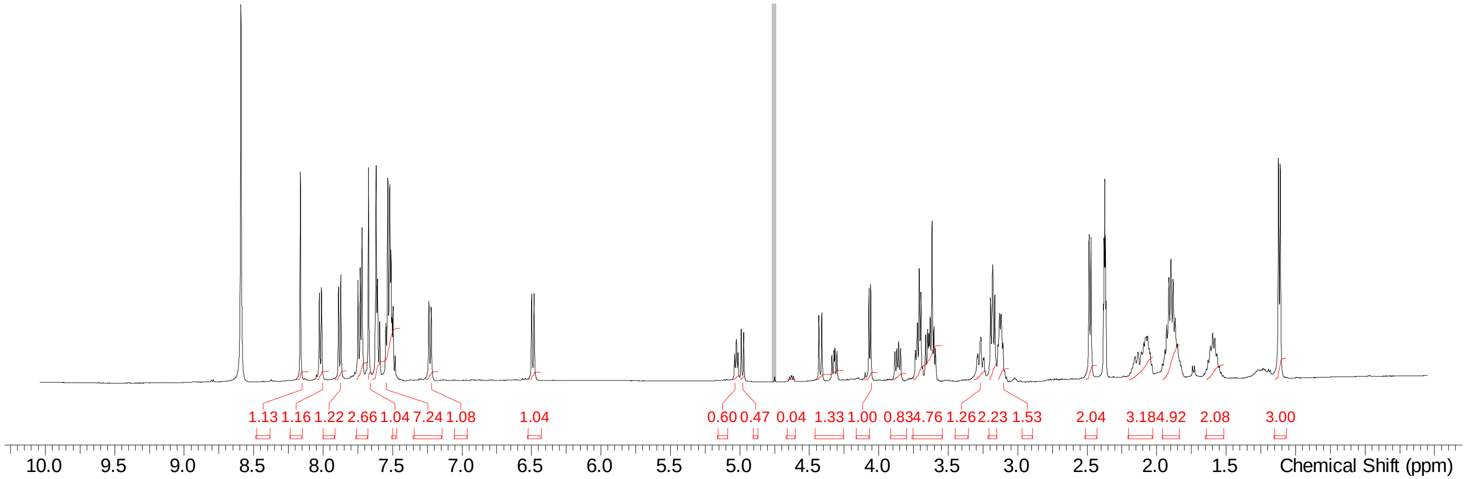

Figure S 61: Water suppressed ^{1}H spectrum of D36 in ACN/D2O + 1% FA at 45 °C and 500 MHz.

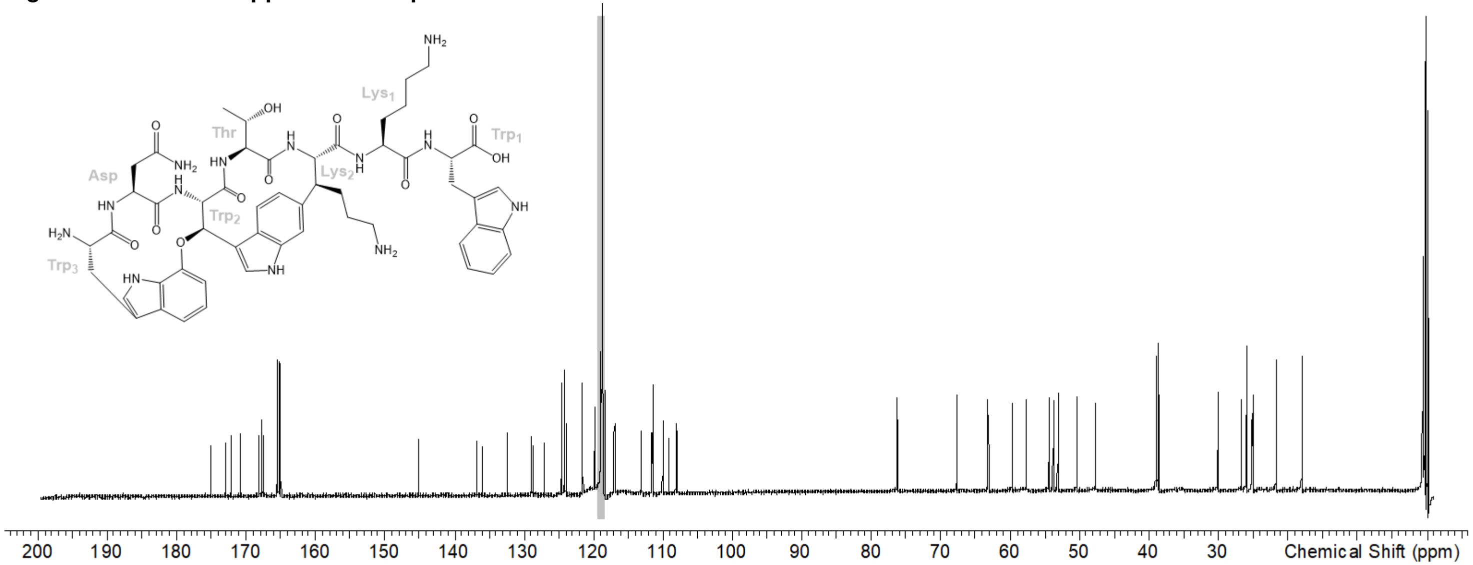

Figure S 62: ^{13}C spectrum of D36 in ACN/D2O + 1% FA at 45 °C and 125 MHz.

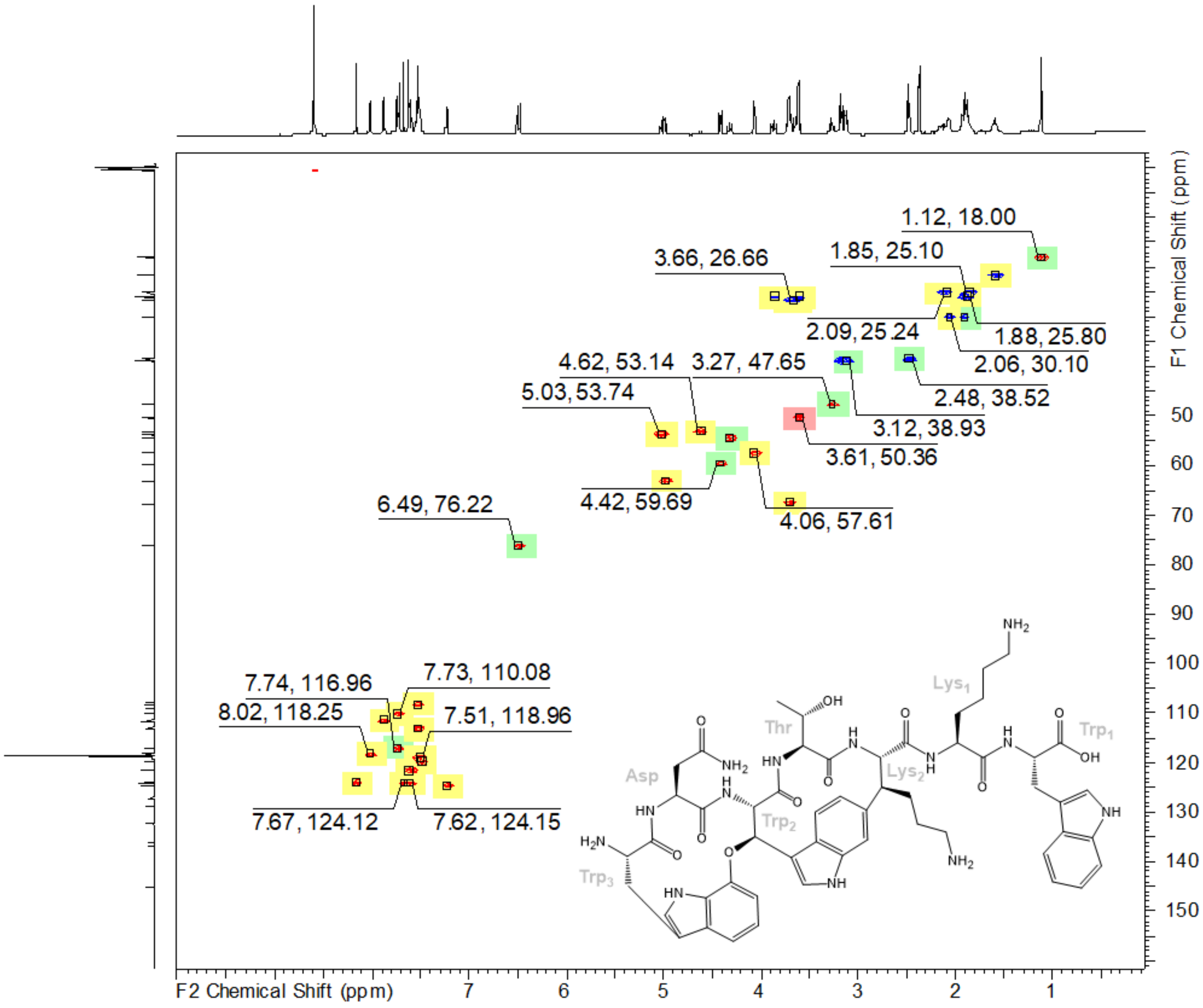

Figure S 63: HSQC spectrum of D36 in ACN/D2O + 1% FA at 45 °C and 500/125 MHz.

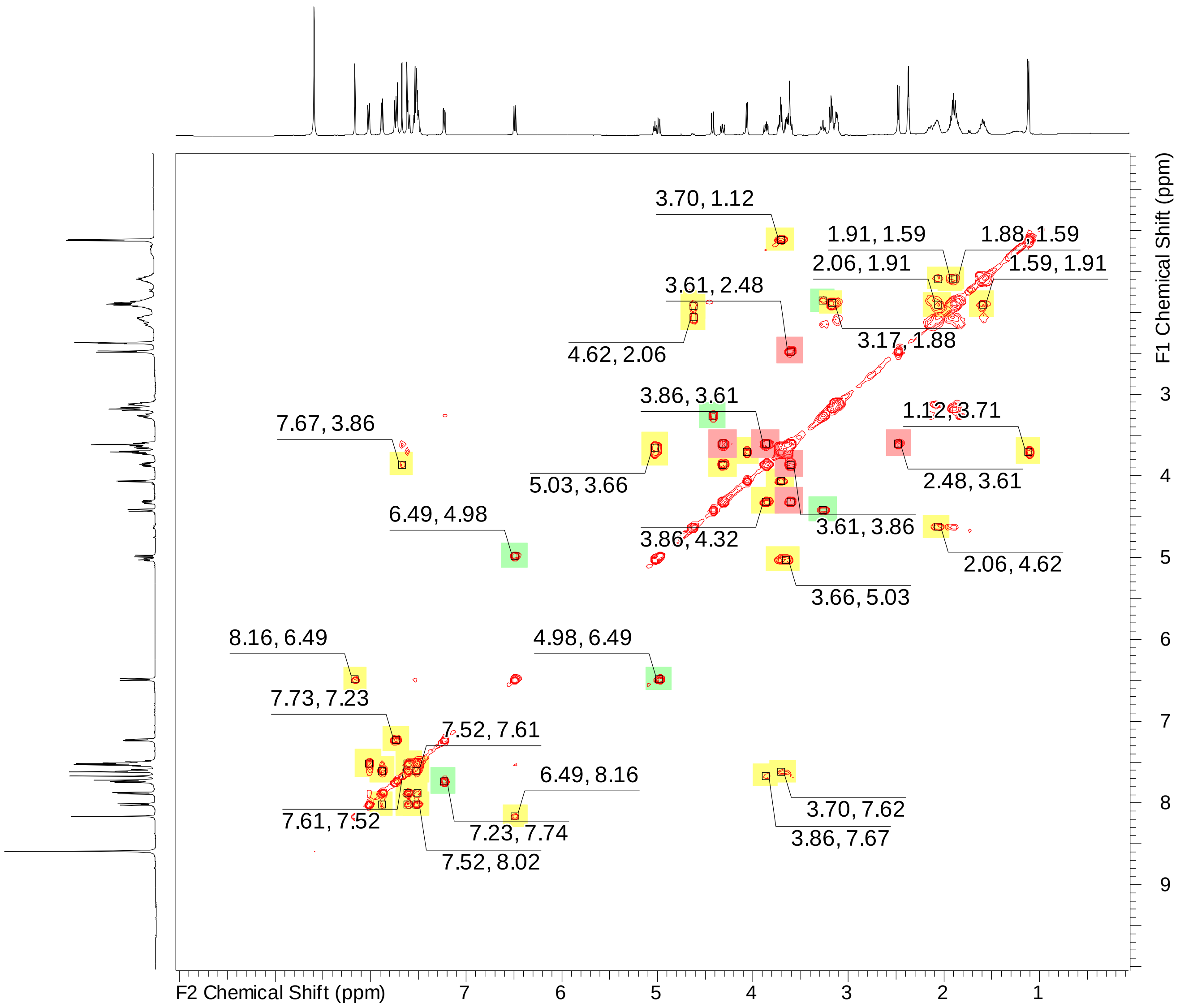

Figure S 64: COSY spectrum of D36 in ACN/D2O + 1% FA at 45 °C and 500/125 MHz.

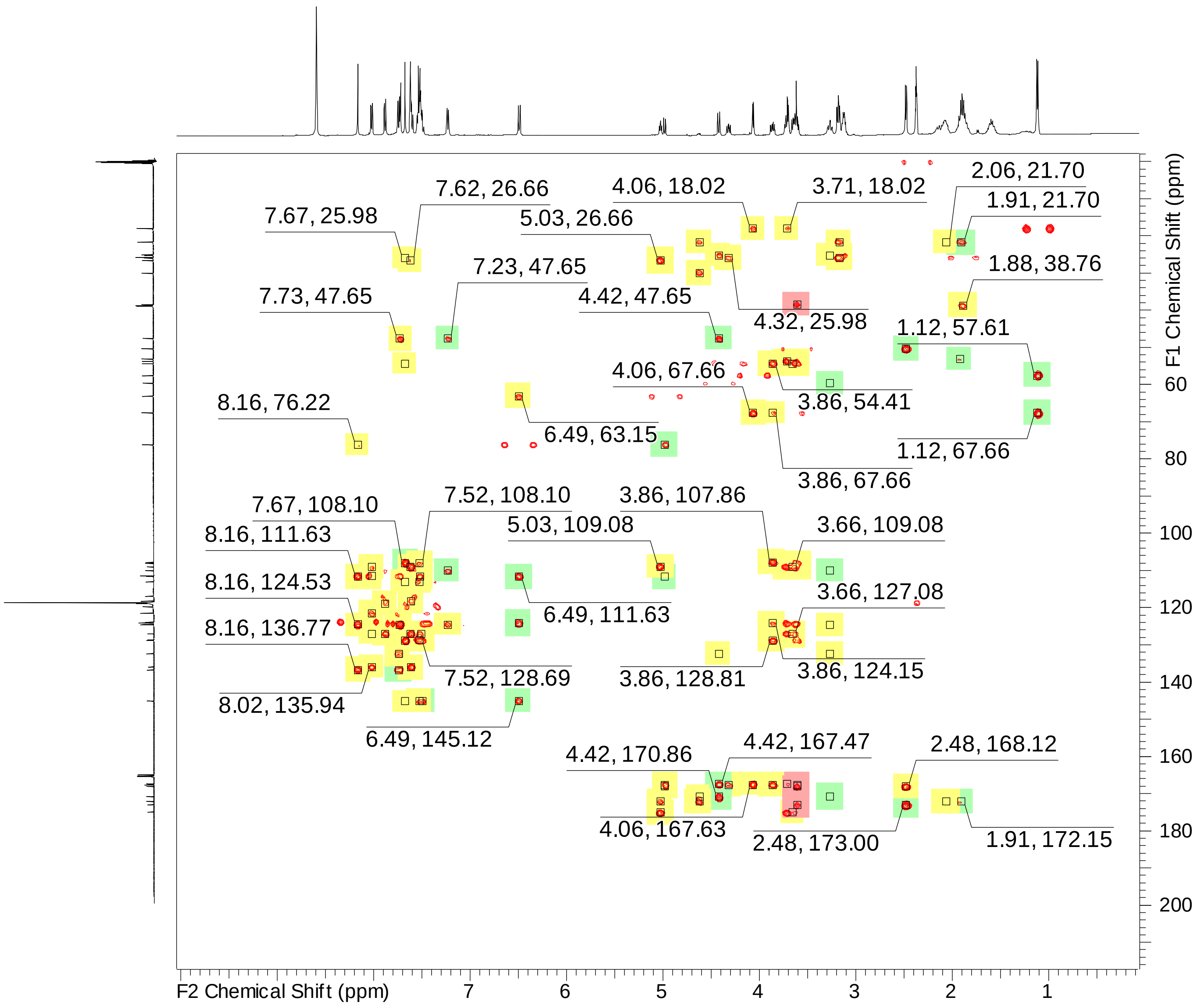

Figure S 65: HMBC spectrum of D36 in ACN/D2O + 1% FA at 45 °C and 500/125 MHz.

D37

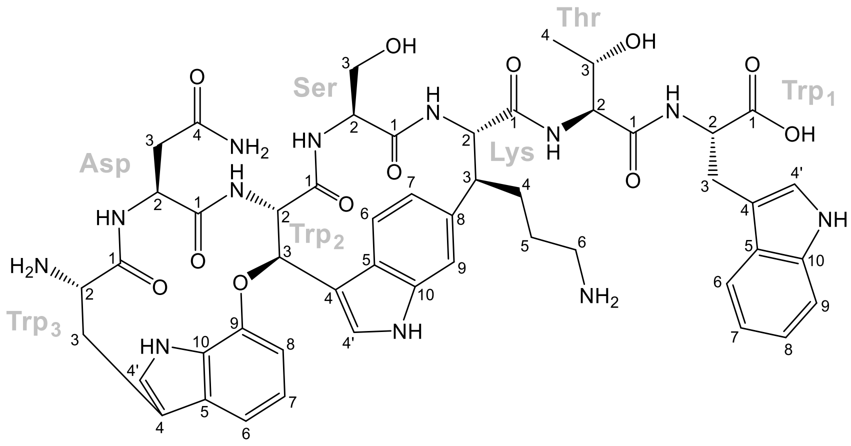

Table S 11: NMR spectroscopic data of D37.

		NMR data in ACN/D_2O + 1% FA-d_4		
position	δ ^{13}C	δ ^{1}H, mult (*J* in Hz)	COSY correlations	HMBC correlations
Trp₃				
1	174.9	-	-	-
2	53.9	5.17, t (5.8)	3	1, 3, 4, *Thr*-1
3	27.0	3.79, m	2	1, 2, 3
4	109.2	-	-	-
4'	124.3	7.72, s	-	3, 4, 5, 9, 10
5	127.4	-	-	-
6	118.6	8.12, d (7.9)	7	8, 10
7	119.1	7.61, m	6, 8	5, 9
8	121.8	7.71, m	7, 9	6, 10
9	111.7	7.99, d (8.2)	8	5, 7
10	136.2	-	-	-
Thr				
1	170.4	-	-	-
2	58.7	4.80, m	3	1, 3, 4, *Lys*-1
3	66.7	4.62, m	2, 4	1, 2, 4
4	18.6	1.51, d (6.3)	3	2, 3
Lys				
1	171.4	-	-	-
2	60.1	4.58, bd (10.7)	3	1, 3, 4, *Ser*-1, *Trp₂*-8
3	48.1	3.39, m	2, 4	1, 2, 4, 5, *Trp₂*-7, *Trp₃*-8, *Trp₂*-9
4	25.3	1.97, 2.18, m	3, 5	3, 5, 6, *Trp₂*-8
5	26.1	2.08, m	4, 6	3, 4, 6
6	39.1	3.21, m	5	4, 5

position	δ ^{13}C	NMR data in ACN/D_2O + 1% FA-d_4<br>δ ^{1}H, mult (*J* in Hz)	COSY correlations	HMBC correlations
Ser				
1	167.9	-	-	-
2	53.9	4.40, m	3	1, 3, *Trp$_2$*-1
3	61.9	3.63, 3.55, m	2	1, 2
Trp$_2$				
1	167.6	-	-	-
2	63.3	5.07, d (8.9)	3	1, 3, 4, *Asp*-1
3	76.3	6.56, d (9.0)	2, 4‘	2, 4, 4‘, *Trp$_3$*-9
4	111.9	-	-	-
4‘	124.1	8.25, s	3	3, 4, 5, 10
5	124.9	-	-	-
6	117.0	7.84, bd (8.1)	7	4, 8, 10
7	124.9	7.34, bd (8.2)	6	5, 9, *Lys*-3
8	132.6	-	-	-
9	110.3	7.82, s	-	5, 7, *Lys*-3
10	136.9	-	-	-
Asp				
1	168.2	-	-	-
2	50.5	3.74, m	3	1, 3, 4, *Trp3*-1
3	38.6	2.56, bd (6.0)	2	1, 2, 4
4	173.0	-	-	-
Trp$_1$				
1	167.8	-	-	-
2	54.6	4.42, m	3	1, 3
3	26.2	3.69, 3.95, dd (13.7, 7.3)	2	1, 2, 4, 4‘, 5
4	108.1	-	-	-
4‘	124.3	7.73, s	-	3, 4, 5, 9, 10
5	128.8	-	-	-
6	119.9	7.56, m	7	4, 8, 10
7	113.1	7.59, m	6, 8	5, 9
8	108.0	7.59, m	7	6, 10
9	145.4	-	-	-
10	128.5	-	-	-

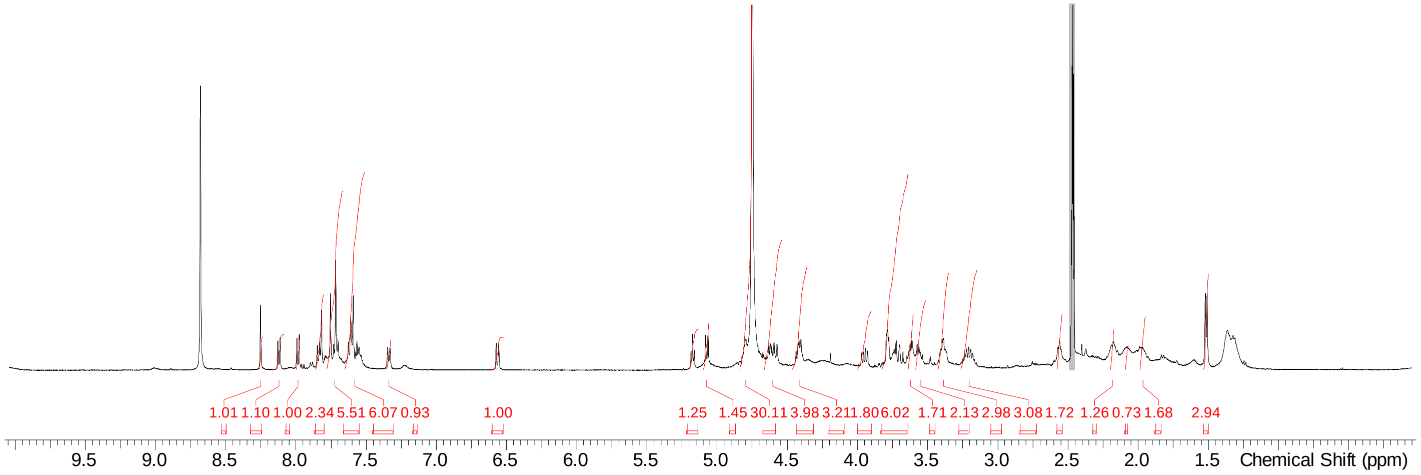

Figure S 66: ^{1}H spectrum of D37 in ACN/D2O + 1% FA at 45 °C and 500 MHz.

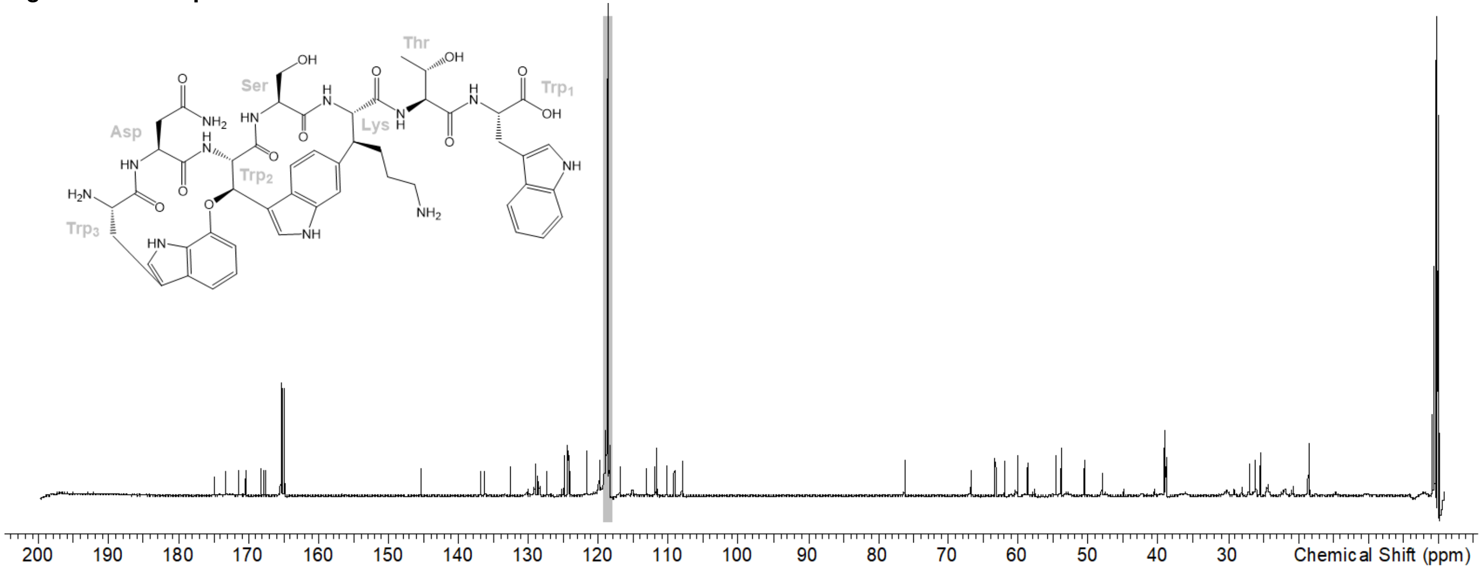

Figure S 67: ^{13}C spectrum of D37 in ACN/D2O + 1% FA at 45 °C and 125 MHz.

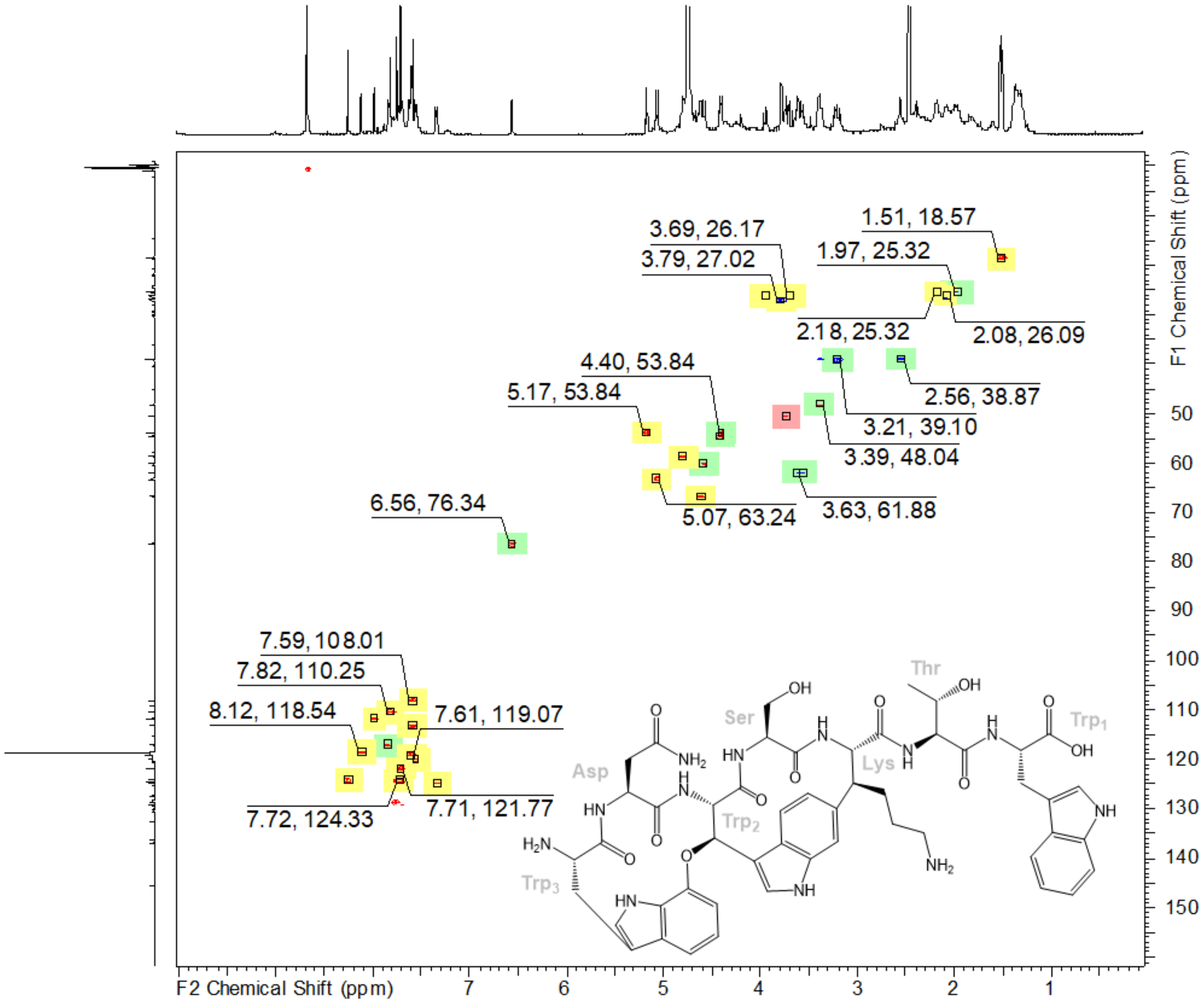

Figure S 68: HSQC spectrum of D37 in ACN/D2O + 1% FA at 45 °C and 500/125 MHz.

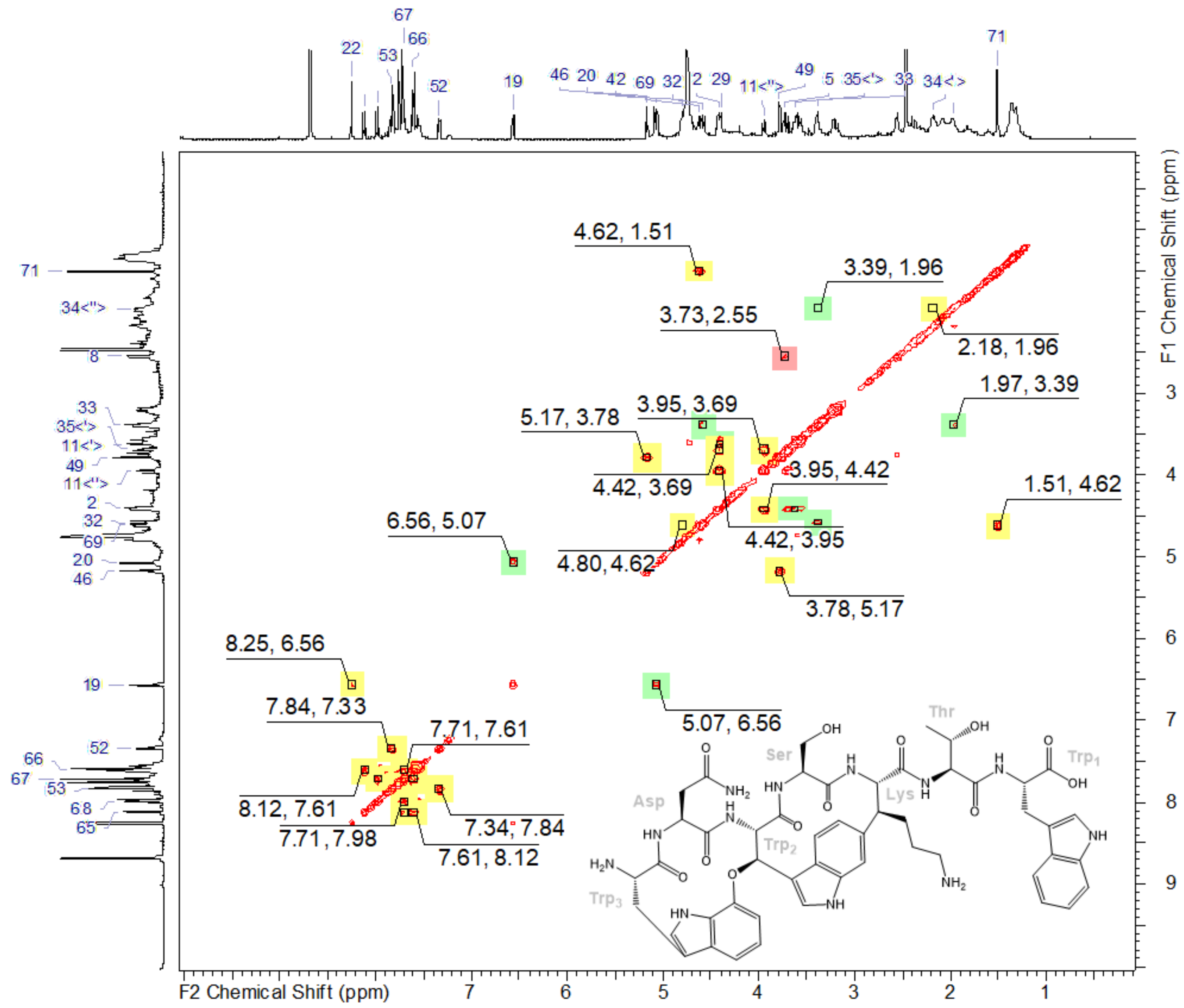

Figure S 69: COSY spectrum of D37 in ACN/D2O + 1% FA at 45 °C and 500/125 MHz.

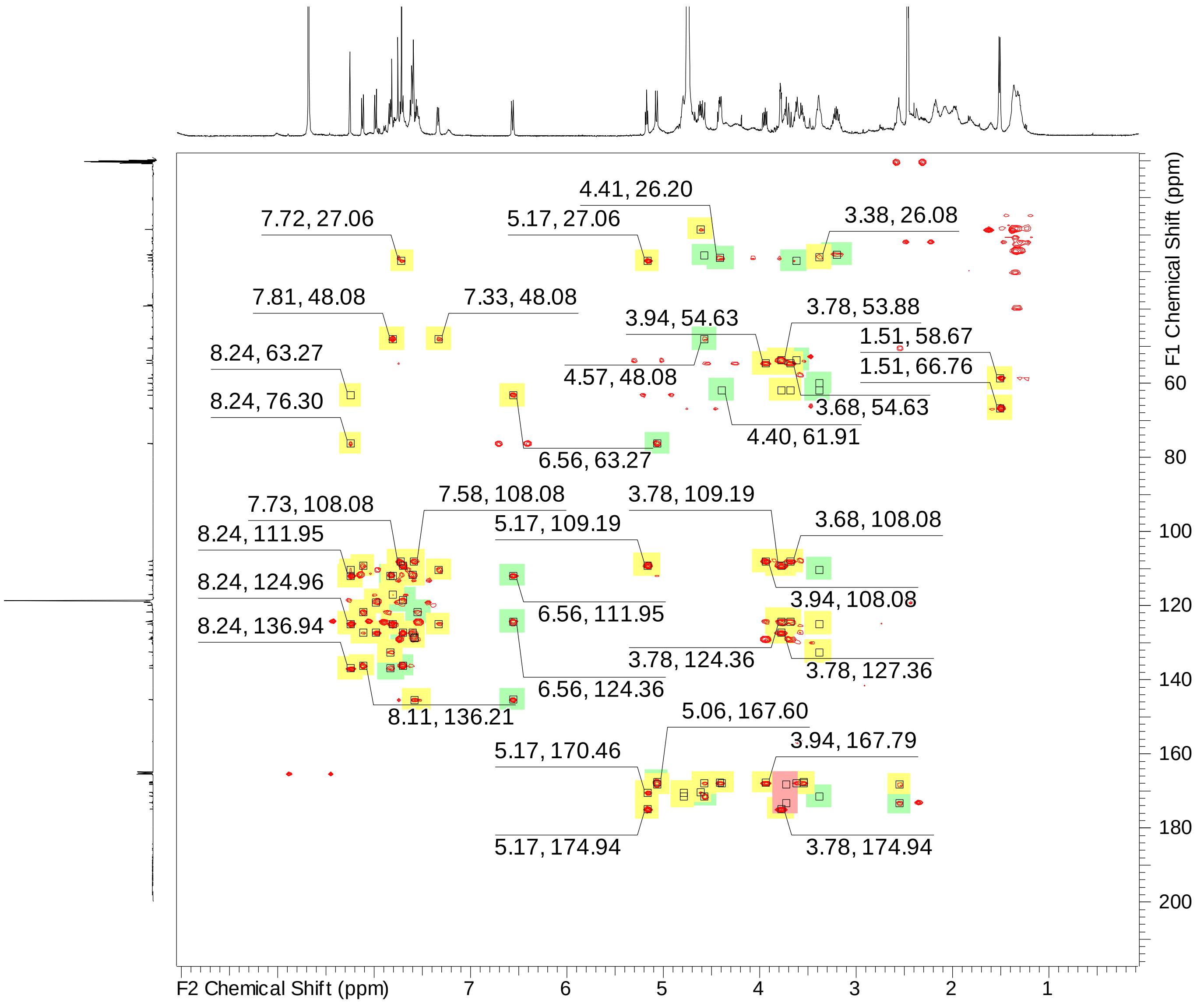

Figure S 70: HMBC spectrum of D37 in ACN/D2O + 1% FA at 45 °C and 500/125 MHz.

D38

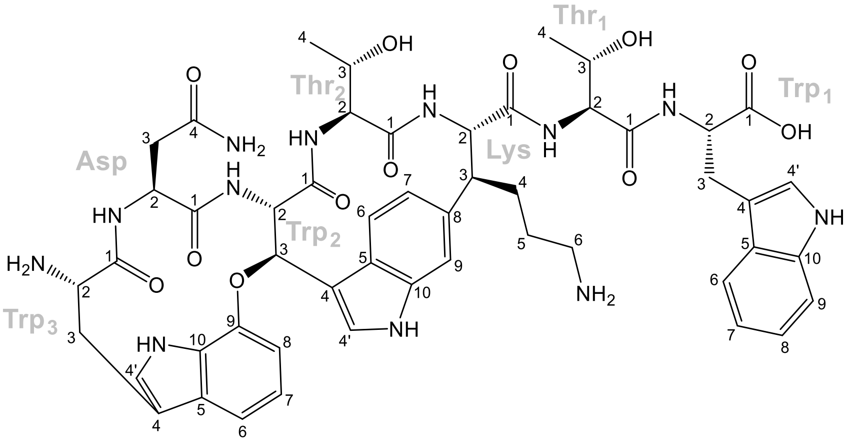

Table S 12: NMR spectroscopic data of D38.

		NMR data in ACN/D_2O + 1% FA-d_4		
position	δ ^{13}C	δ ^{1}H, mult (*J* in Hz)	COSY correlations	HMBC correlations
Trp₁				
1	174.7	-	-	-
2	53.5	5.05, t (6.7)	3	1, 3, 4, *Thr₁*-1
3	26.7	3.66, m	2	1, 2, 3
4	108.8	-	-	-
4’	124.2	7.60, s	-	3, 4, 5, 9, 10
5	127.0	-	-	-
6	118.3	8.00, d (7.9)	7	8, 10
7	118.9	7.50, m	6, 8	5, 9
8	121.6	7.60, m	7, 9	6, 10
9	111.5	7.87, d (8.2)	8	5, 7
10	135.9	-	-	-
Thr₁				
1	170.2	-	-	-
2	58.4	4.66, bd (3.9)	3	1, 3, 4, *Lys*-1
3	66.3	4.48, m	2, 4	1, 2, 4
4	18.3	1.35, d (6.4)	3	2, 3
Lys				
1	171.1	-	-	-
2	59.7	4.38, d (10.5)	3	1, 3, 4, *Thr₂*-1, *Trp₂*-8
3	47.6	3.25, m	2, 4	1, 2, 4, 5, *Trp₂*-7, *Trp₃*-8, *Trp₂*-9
4	25.0	1.89, 2.00, m	3, 5	3, 5, 6, *Trp₂*-8
5	25.9	1.94, m	4, 6	3, 4, 6
6	38.8	3.03, m	5	4, 5

position	δ ^{13}C	NMR data in ACN/D_2O + 1% FA-d_4<br>δ ^{1}H, mult (*J* in Hz)	COSY correlations	HMBC correlations
Thr$_2$				
1	167.4	-	-	-
2	57.6	4.04, d (6.3)	3	1, 3, 4, *Trp$_2$*-1
3	67.6	3.66, m	2, 4	2, 4
4	18.0	1.06, d (6.3)	3	2, 3
Trp$_2$				
1	167.5	-	-	-
2	63.0	4.95, d (8.9)	3	1, 3, 4, *Asp*-1
3	76.2	6.43, d (8.9)	2, 4‘	2, 4, 4‘, *Trp$_3$*-9
4	111.5	-	-	-
4‘	123.9	8.12, s	3	3, 4, 5, 10
5	124.1	-	-	-
6	116.9	7.68, d (8.2)	7	4, 8, 10
7	124.5	7.18, d (8.2)	6	5, 9, *Lys*-3
8	132.3	-	-	-
9	110.0	7.67, s	-	5, 7, *Lys*-3
10	136.7	-	-	-
Asp				
1	168.0	-	-	-
2	50.3	3.58, m	3	1, 3, 4, *Trp3*-1
3	38.4	2.43, d (6.9)	2	1, 2, 4
4	172.9	-	-	-
Trp$_3$				
1	167.6	-	-	-
2	54.3	4.28, dd (10.9, 7.3)	3	1, 3
3	25.9	3.58, 3.82, dd (13.3, 7.1)	2	1, 2, 4, 4‘, 5
4	107.7	-	-	-
4‘	124.1	7.62, s	-	3, 4, 5, 9, 10
5	128.6	-	-	-
6	119.7	7.44, m	7	4, 8, 10
7	113.0	7.47, m	6, 8	5, 9
8	108.0	7.46, m	7	6, 10
9	145.0	-	-	-
10	128.7	-	-	-

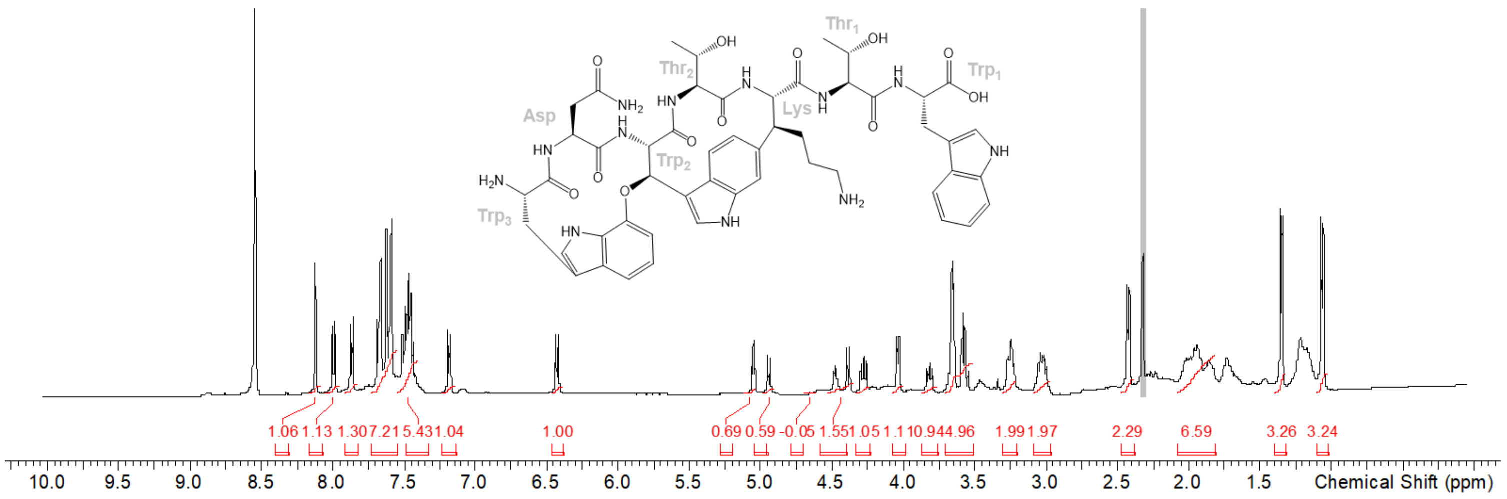

Figure S 71: Water-suppressed ^{1}H spectrum of D38 in ACN/D2O + 1% FA at 45 °C and 500 MHz.

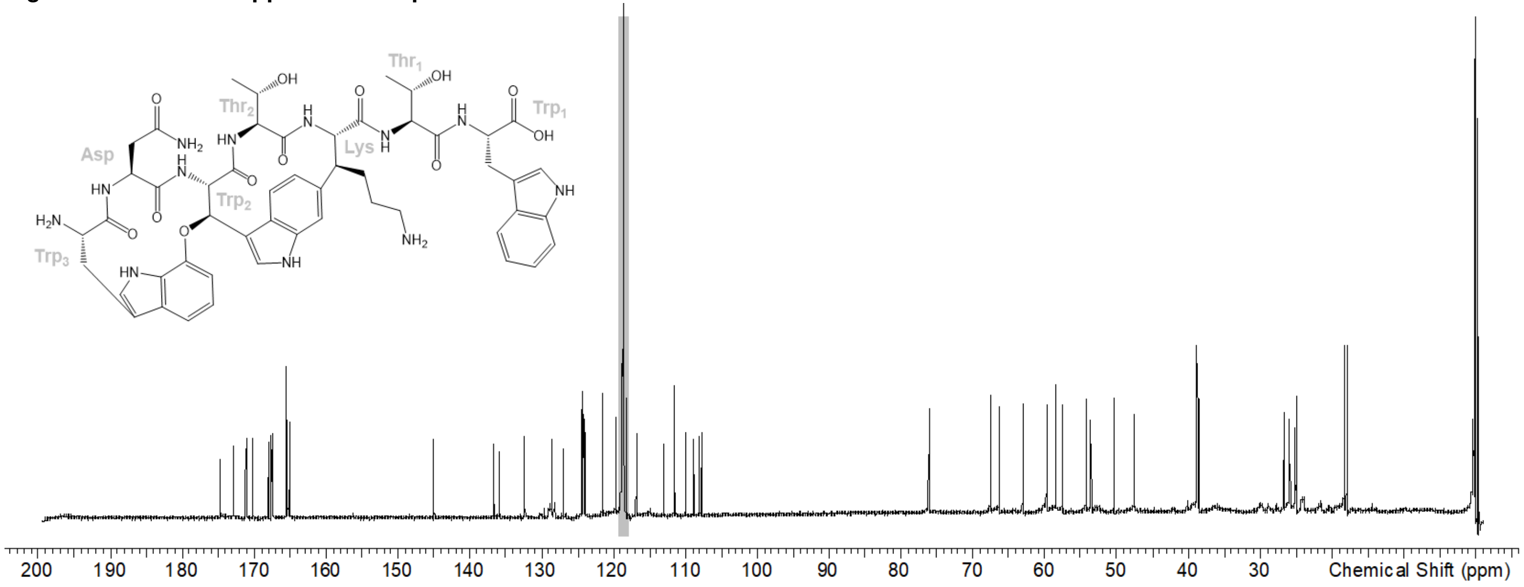

Figure S 72: ^{13}C spectrum of D38 in ACN/D2O + 1% FA at 45 °C and 125 MHz.

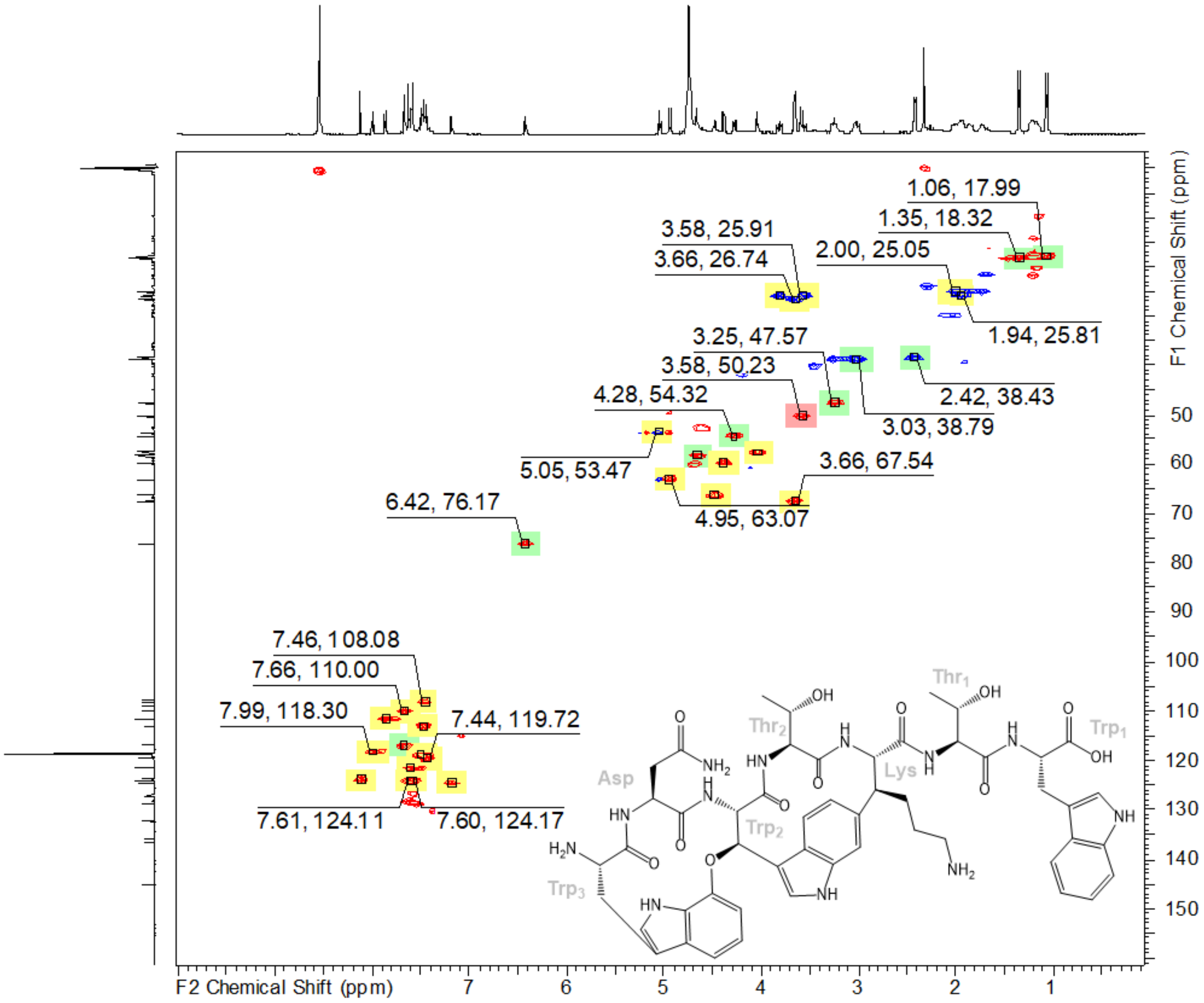

Figure S 73: HSQC spectrum of D38 in ACN/D2O + 1% FA at 45 °C and 500/125 MHz.

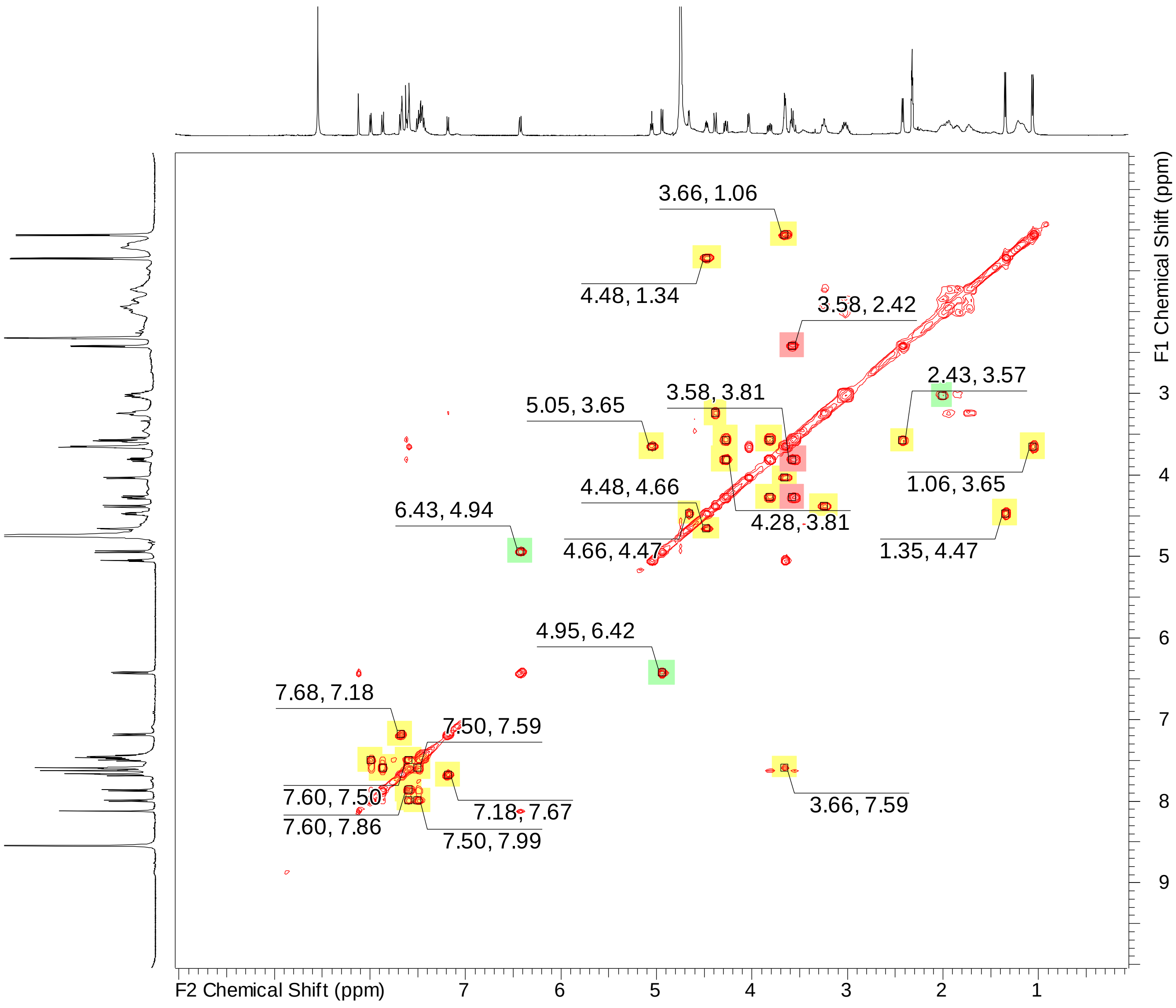

Figure S 74: COSY spectrum of D38 in ACN/D2O + 1% FA at 45 °C and 500/125 MHz.

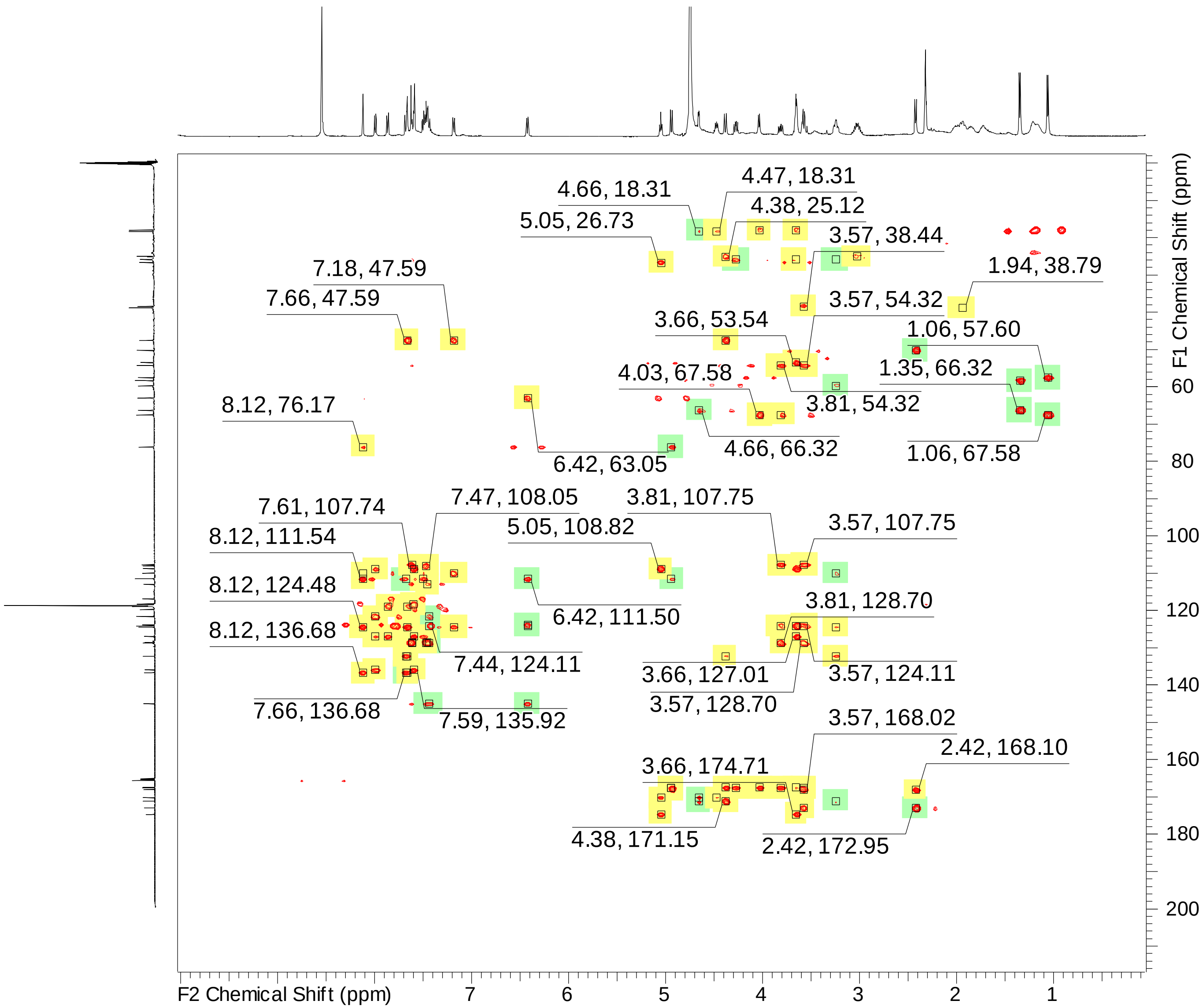

Figure S 75: HMBC spectrum of D38 in ACN/D2O + 1% FA at 45 °C and 500/125 MHz.

D6[R]

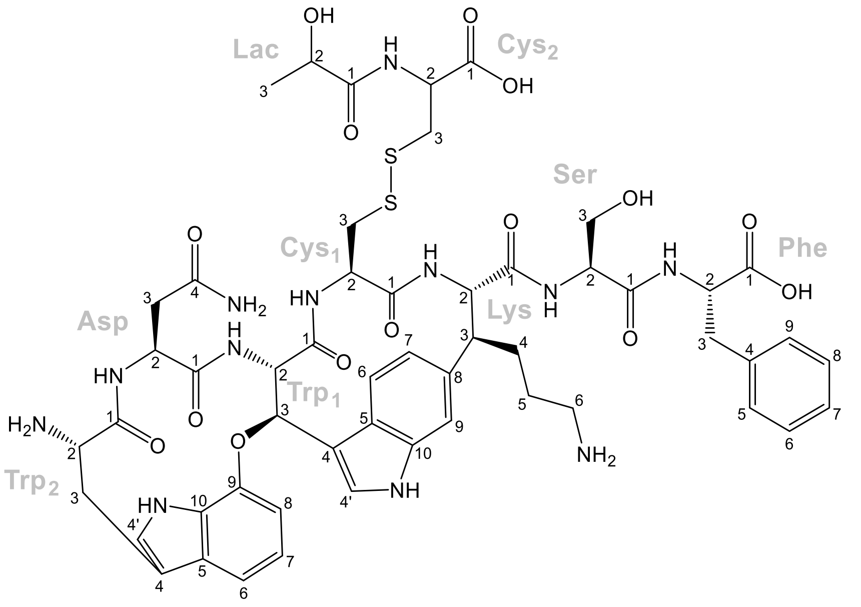

Table S 13: NMR spectroscopic data of D6[R].

		NMR data in ACN/D_2O + 1% FA-d_4		
position	δ ^{13}C	δ ^{1}H, mult (*J* in Hz)	COSY correlations	HMBC correlations
Phe				
1	174.0	-	-	-
2	54.3	5.07, m	3	1, 3, 4, *Ser*-1
3	36.7	3.64, 3.51, m	2	1, 2, 4, 5/9
4	136.7	-	-	-
5/9	129.4	7.70, m	6/8	3, 7
6/8	129.4	7.80, m	5/9, 7	4, 6/8
7	127.0	7.75, m	6/8	5/9
Ser				
1	167.8	-	-	-
2	54.2	4.35, m	3	1, 3, *Lys*-1
3	62.2	3.61, 3.49, m	2	1, 2
Lys				
1	167.7	-	-	-
2	60.2	4.58, m	3	1, 3, 4, *Cys*-1, Trp_1-8
3	48.2	3.41, m	2, 4	1, 2, 4, 5, Trp_1-7, Trp_3-8, Trp_1-9
4	25.8	2.48, 2.13, m	3, 5	3, 5, 6, Trp_1-8
5	25.5	2.29, m	4, 6	3, 4, 6
6	39.4	3.40, m	5	4, 5

		NMR data in ACN/D_2O + 1% FA-d_4		
position	δ ^{13}C	δ ^{1}H, mult (*J* in Hz)	COSY correlations	HMBC correlations
Cys_1				
1	171.2	-	-	-
2	52.6	5.07, m	3	1, 3, *Trp_1*-1
3	39.0	3.29, 3.50, m	2	1, 2, *Cys_2*-3
Cys_2				
1	173.4	-	-	-
2	52.6	5.10, m	3	1, 3, *Lac*-1
3	39.3	3.46, 3.67, m	2	1, 2, *Cys_1*-3
Lac				
1	176.7	-	-	-
2	67.6	4.65, m	3	1, 3
3	19.8	1.75, t (5.3)	2	1, 2
Trp_1				
1	170.6	-	-	-
2	63.4	5.03, d (9.0)	3	1, 3, 4, *Asp*-1
3	76.4	6.54, d (8.9)	2, 4‘	2, 4, 4‘, *Trp_2*-9
4	112.0	-	-	-
4‘	124.2	8.23, s	3	3, 4, 5, 10
5	125.0	-	-	-
6	117.2	7.82, d (8.3)	7	4, 8, 10
7	125.0	7.32, d (8.2)	6	5, 9, *Lys*-3
8	132.6	-	-	-
9	110.4	7.83, s	-	5, 7, *Lys*-3
10	137.1	-	-	-
Asp				
1	168.3	-	-	-
2	50.7	3.67, m	3	1, 3, 4, *Trp_2*-1
3	39.0	2.54, t (6.1)	2	1, 2, 4
4	173.3	-	-	-
Trp_2				
1	167.8	-	-	-
2	54.7	4.38, m	3	1, 3
3	26.3	3.67, 3.92, m	2	1, 2, 4, 4‘, 5
4	108.1	-	-	-
4‘	120.0	7.55, s	-	3, 4, 5, 9, 10
5	129.4	-	-	-
6	124.4	7.73, m	7	4, 8, 10
7	113.2	7.57, m	6, 8	5, 9
8	108.2	7.59, m	7	6, 10
9	145.5	-	-	-
10	129.1	-	-	-

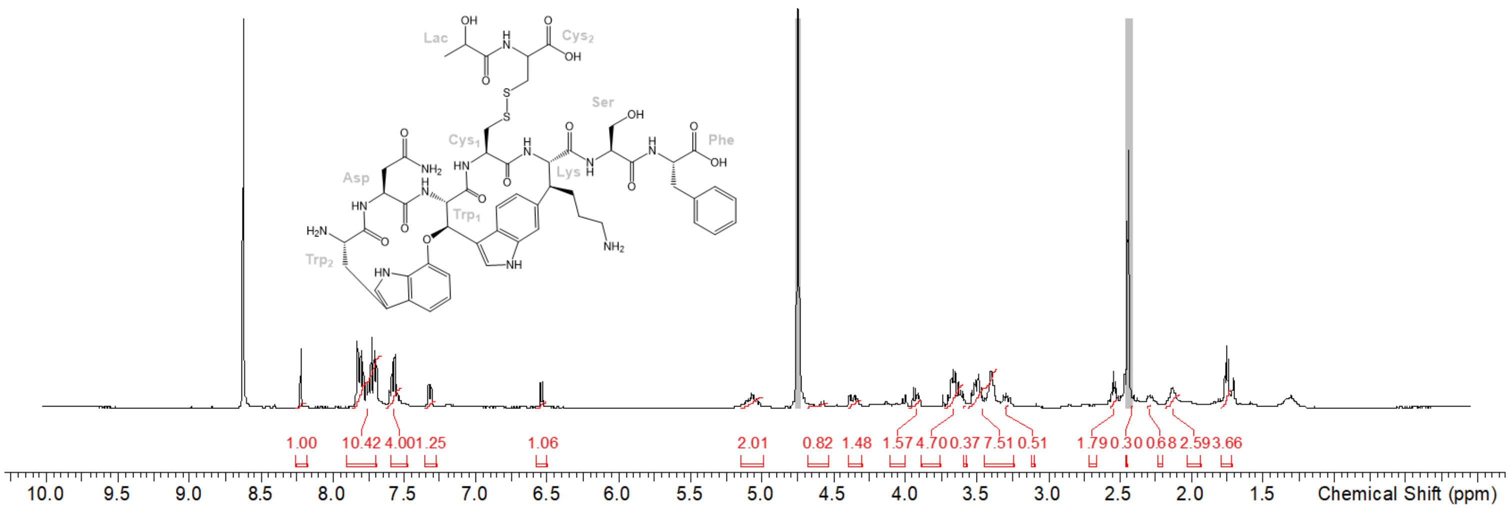

Figure S 76: ^{1}H spectrum of D6[R] in ACN/D2O + 1% FA at 45 °C and 500 MHz.

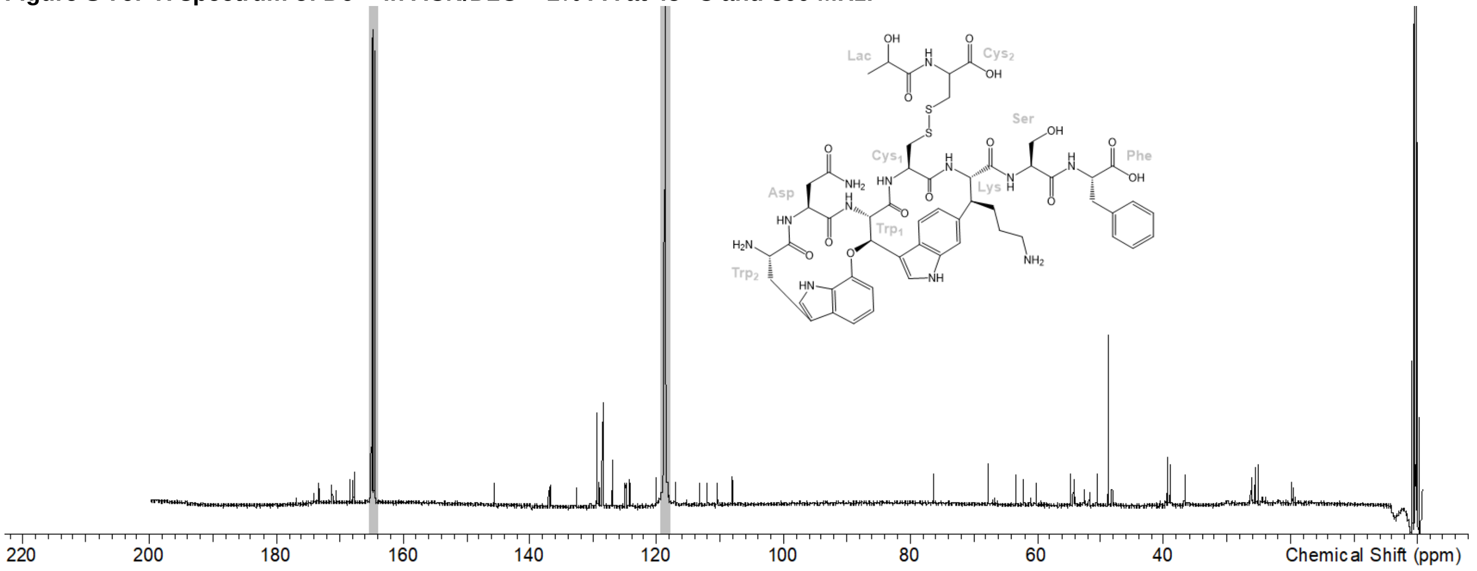

Figure S 77: ^{13}C spectrum of D6[R] in ACN/D2O + 1% FA at 45 °C and 125 MHz.

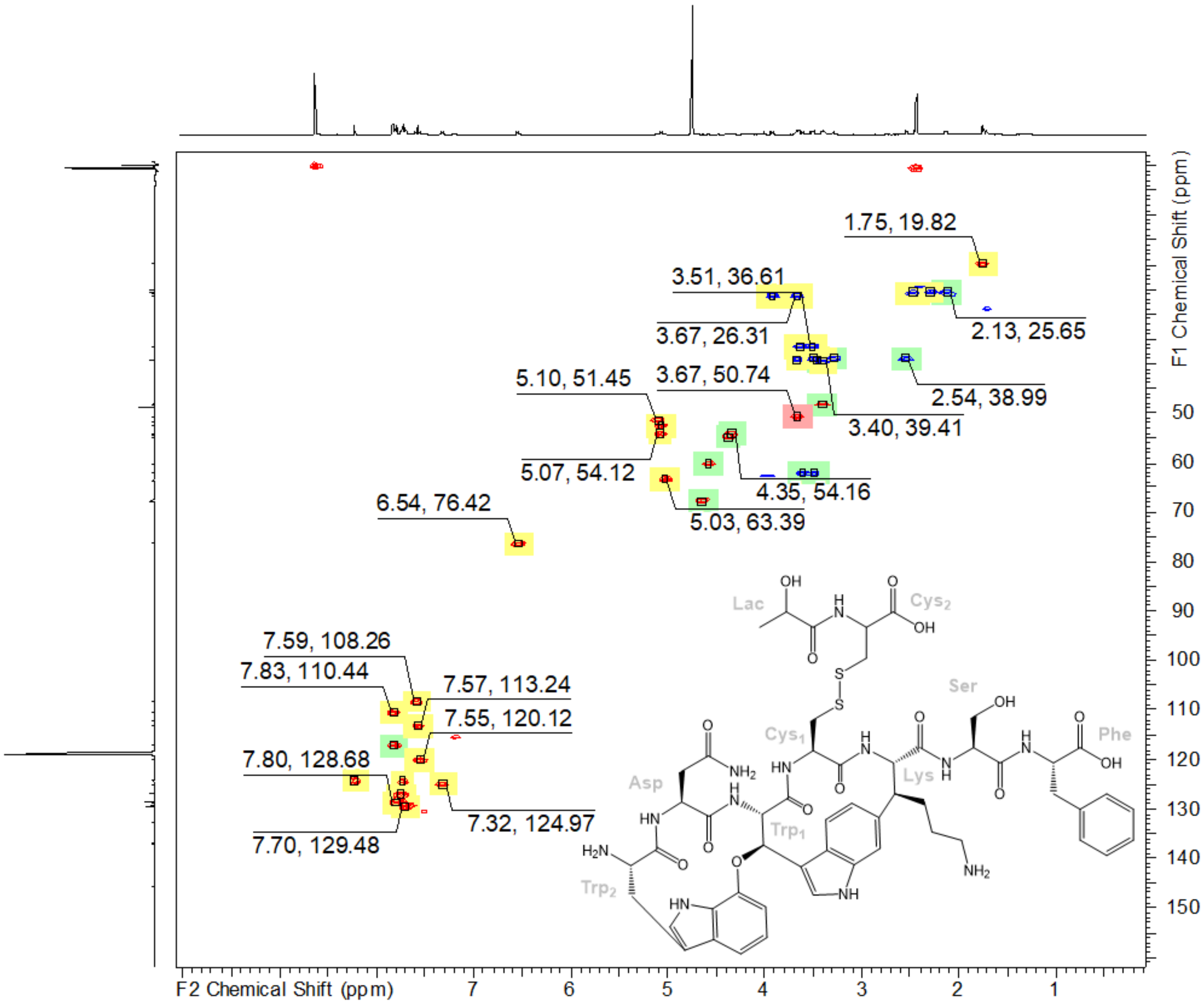

Figure S 78: HSQC spectrum of D6[R] in ACN/D2O + 1% FA at 45 °C and 500/125 MHz.

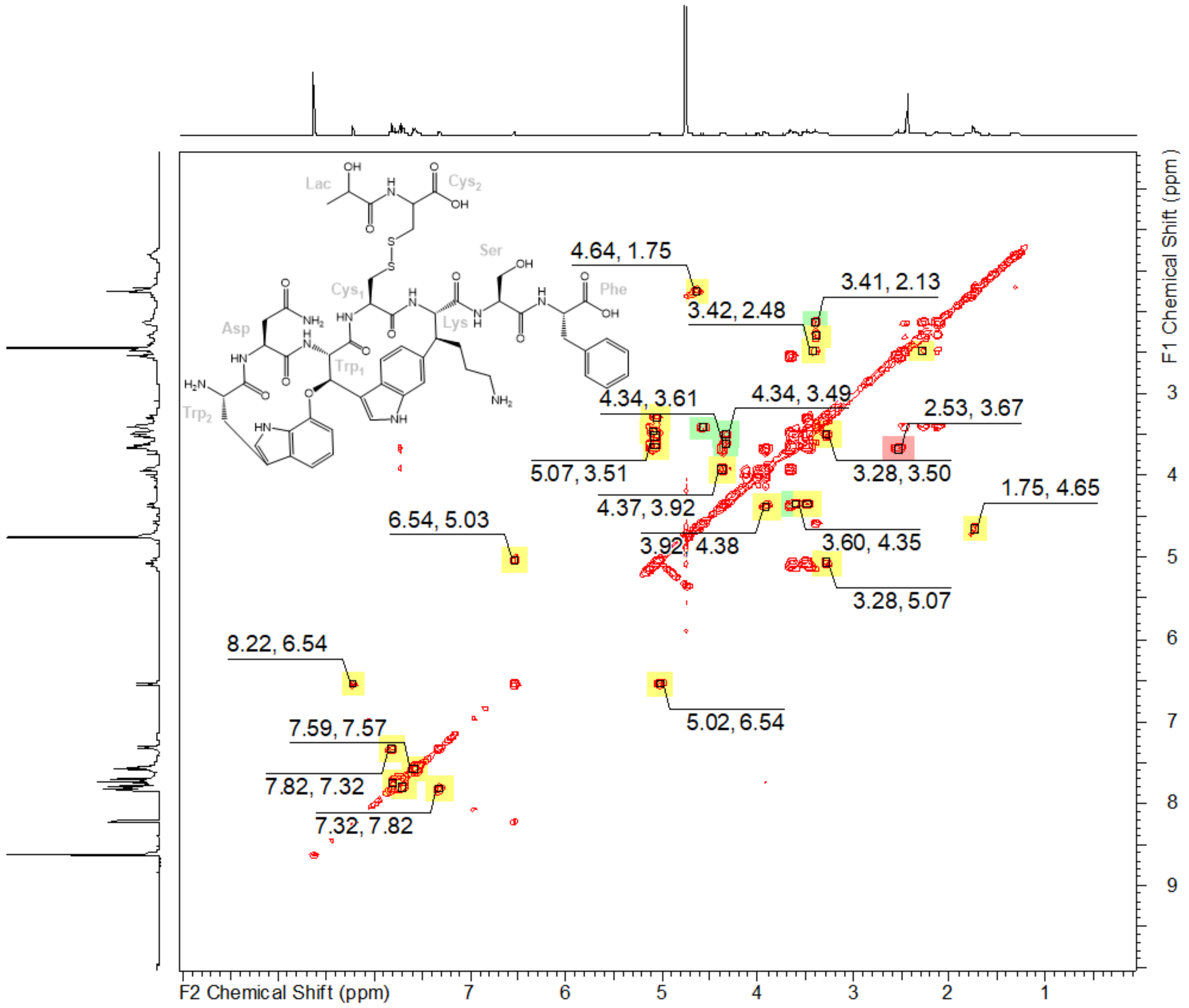

Figure S 79: COSY spectrum of D6[R] in ACN/D2O + 1% FA at 45 °C and 500/125 MHz.

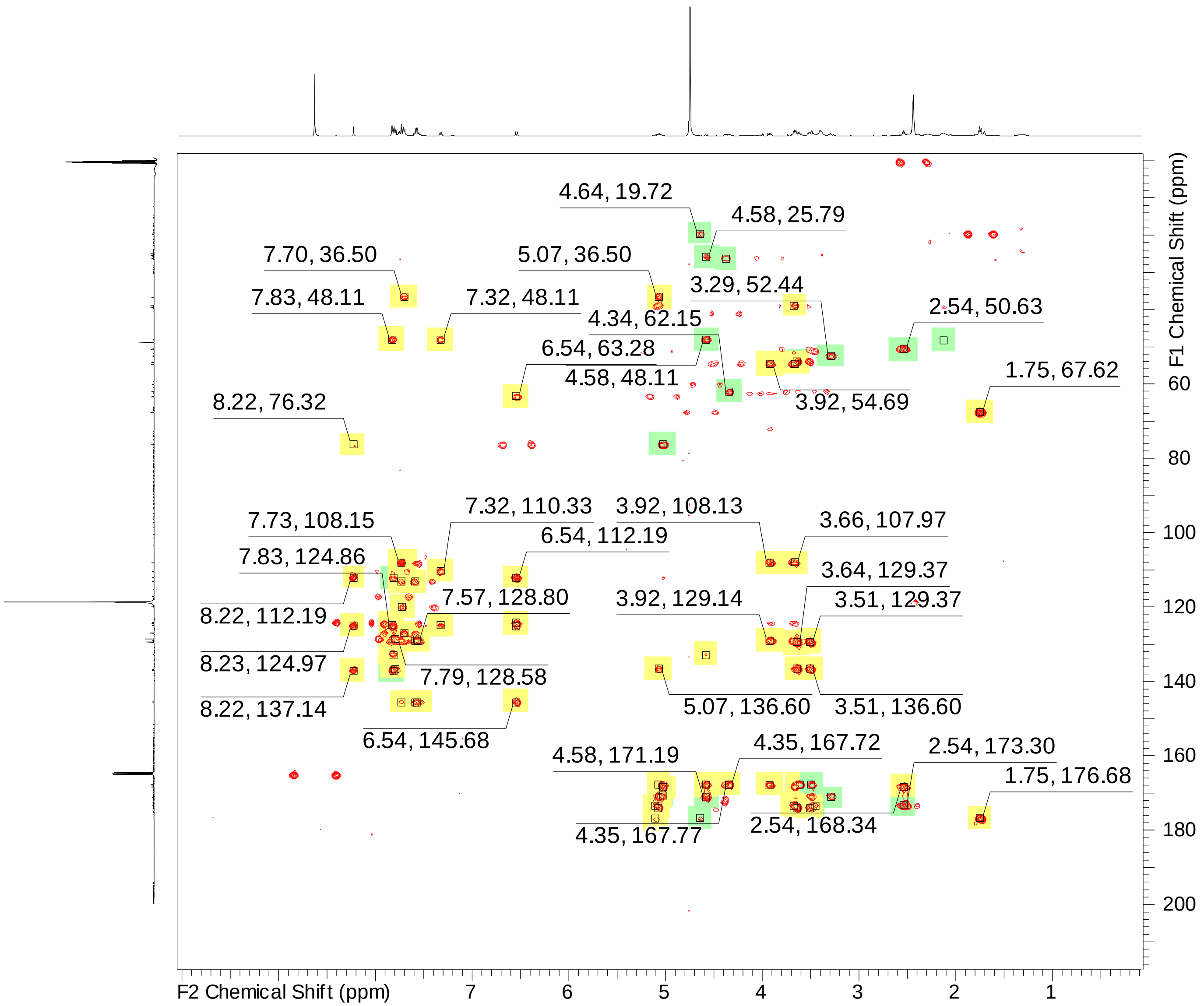

Figure S 80: COSY spectrum of D6[R] in ACN/D2O + 1% FA at 45 °C and 500 MHz.

D32[R]

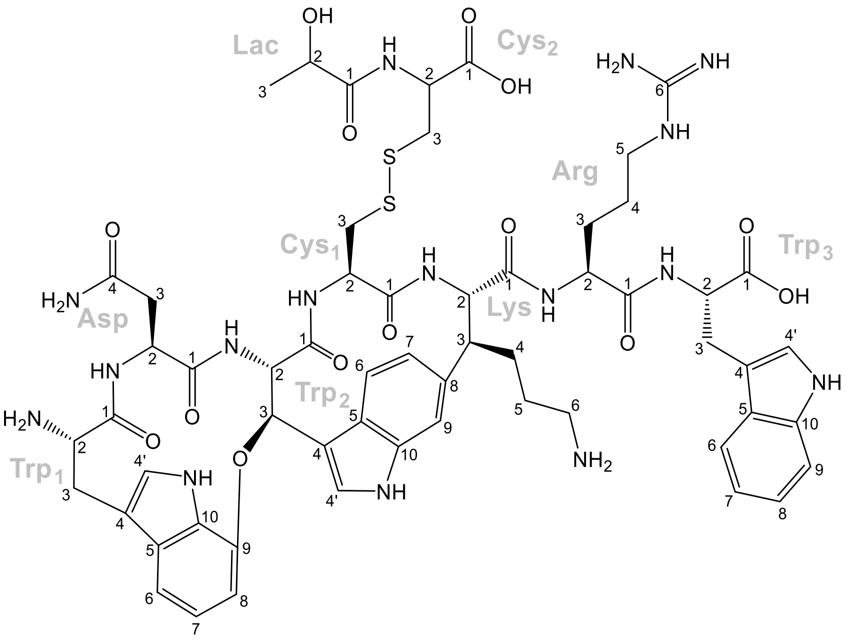

Table S 14: NMR spectroscopic data of D32[R].

		NMR data in ACN/D_2O + 1% FA-d_4		
position	δ ^{13}C	δ ^{1}H, mult (*J* in Hz)	COSY correlations	HMBC correlations
Trp$_3$				
1	175.1	-	-	-
2	53.6	4.61, t (5.8)	3	1, 3, 4, 4‘, *Asp*-1
3	26.8	3.26, m	2, 4‘	1, 2, 4, 4‘, 5
4	109.1	-	-	-
4‘	124.2	7.19, s	3	3, 4, 5, 10
5	127.2	-	-	-
6	118.3	7.57, d (7.9)	7	4, 5, 8, 9, 10
7	119.8	7.06, m	6, 8	5, 8, 9
8	111.6	7.43, d (8.0)	7, 9	5, 6, 8, 10
9	121.6	7.17, t (5.6)	8	5, 6, 7, 8, 10
10	136.0	-	-	-
Arg				
1	172.1	-	-	-
2	53.0	4.26, m	3	3, 4
3	28.2	1.64, 1.49, m	2, 4	1, 2, 4, 5
4	24.3	1.35, m	3, 5	2, 3, 5
5	40.3	2.96, m	4	3, 4, 6
6	156.4	-	-	-

		NMR data in ACN/D_2O + 1% FA-d_4		
position	δ ^{13}C	δ ^{1}H, mult (*J* in Hz)	COSY correlations	HMBC correlations
Lys				
1	170.8	-	-	-
2	59.7	4.01, bd (9.9)	3	1, 3, 4, *Cys*-1, *Trp$_1$*-8
3	47.7	2.88, m	2, 4	1, 2, 4, 5, *Trp$_1$*-7, *Trp$_3$*-8, *Trp$_1$*-9
4	25.3	1.46, 1.65, m	3, 5	2, 3, 5, 6
5	25.5	1.73, m	4, 6	3, 4, 6
6	39.0	2.68, bd (4.8)	5	4, 5
Cys$_1$				
1	167.7	-	-	-
2	51.3	4.00, m	3	1, 3, *Trp$_1$*-1
3	40.6	2.31, 2.42, m	2	1, 2
Cys$_2$				
1	174.3	-	-	-
2	52.3	4.35, m	3	1, 3, *Lac*-1
3	39.4	2.98, 2.81, m	2	1, 2
Lac				
1	176.3	-	-	-
2	67.4	4.08, m	3	1, 3
3	19.6	1.19, d (6.9)	2	1, 2
Trp$_2$				
1	167.1	-	-	-
2	63.1	4.51, bd (9.2)	3	1, 3, 4, *Asp*-1
3	76.2	6.01, d (8.7)	2, 4‘	2, 4, 4‘, *Trp$_1$*-9
4	111.7	-	-	-
4‘	124.0	7.70, s	3	3, 4, 5, 6, 9, 10
5	124.7	-	-	-
6	109.9	7.27, m	7	4, 5, 7, 8, 9, 10
7	124.8	6.81, d (7.5)	6	5, 6, 8, 9, 10, *Lys*-3
8	132.4	-	-	-
9	117.1	7.28, s	-	6, 7, *Lys*-3
10	136.8	-	-	-
Asp				
1	168.0	-	-	-
2	50.4	3.23, m	3	1, 3, 4, *Trp$_1$*-1
3	38.8	2.03, m	2	1, 2, 4
4	173.3	-	-	-

		NMR data in ACN/D_2O + 1% FA-d_4		
position	δ ^{13}C	δ ^{1}H, mult (*J* in Hz)	COSY correlations	HMBC correlations
Trp_1				
1	167.8	-	-	-
2	54.4	3.90, m	3	1, 3
3	26.1	3.16, 3.42, m	2, 4‘	1, 2, 4, 4‘, 5
4	107.9	-	-	-
4‘	124.2	7.22, s	3	3, 4, 5, 9
5	128.9	-	-	-
6	113.2	7.06, m	7	4, 8, 9
7	119.0	7.08, m	6, 8	5, 9
8	108.1	7.04, m	7	6, 9, 10
9	145.2	-	-	-
10	128.7	-	-	-

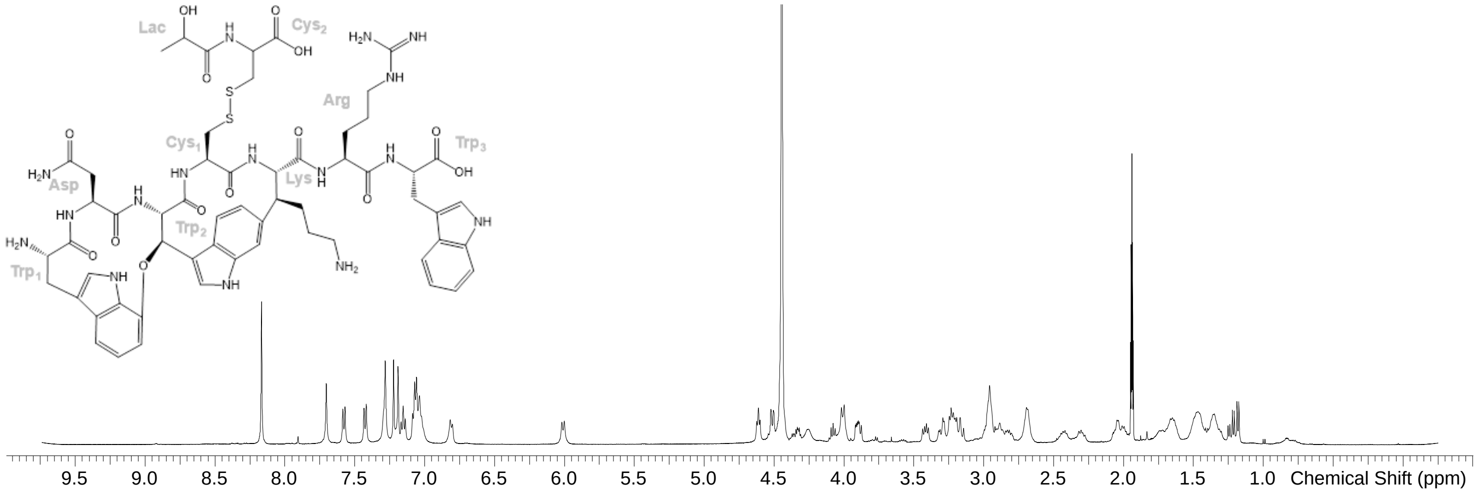

Figure S 81: ^{1}H spectrum of D32[R] in ACN/D2O + 1% FA at 35 °C and 500 MHz.

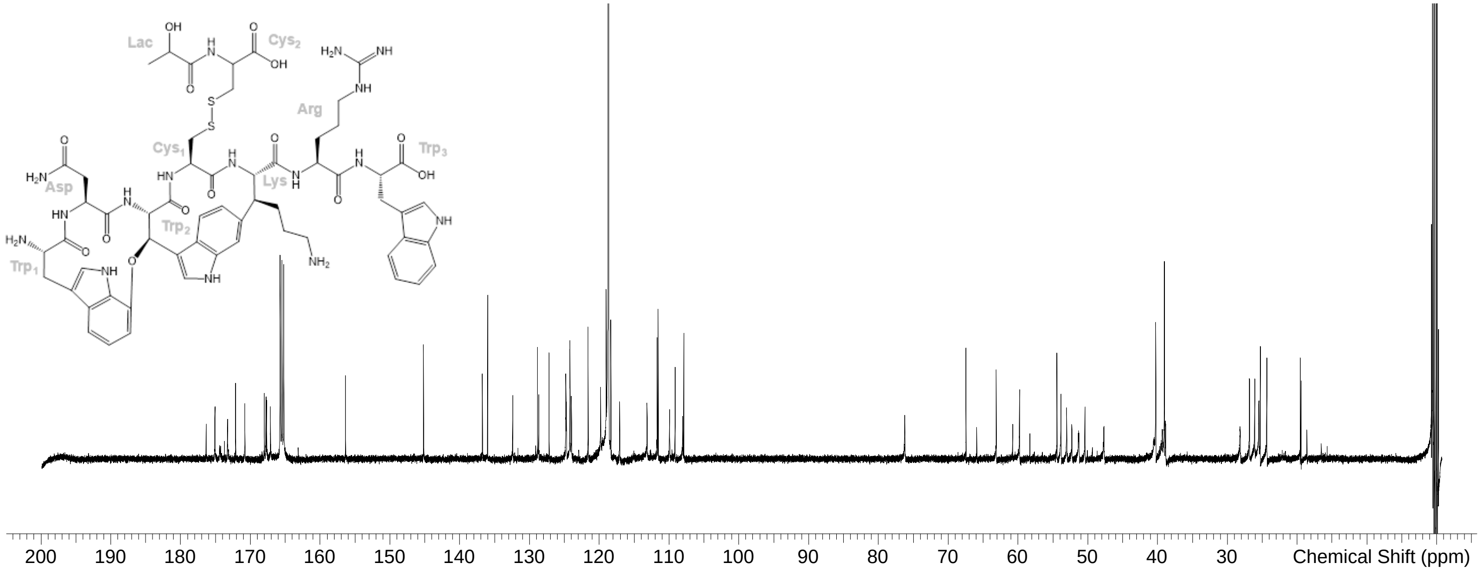

Figure S 82: ^{13}C spectrum of D32[R] in ACN/D2O + 1% FA at 35 °C and 125 MHz.

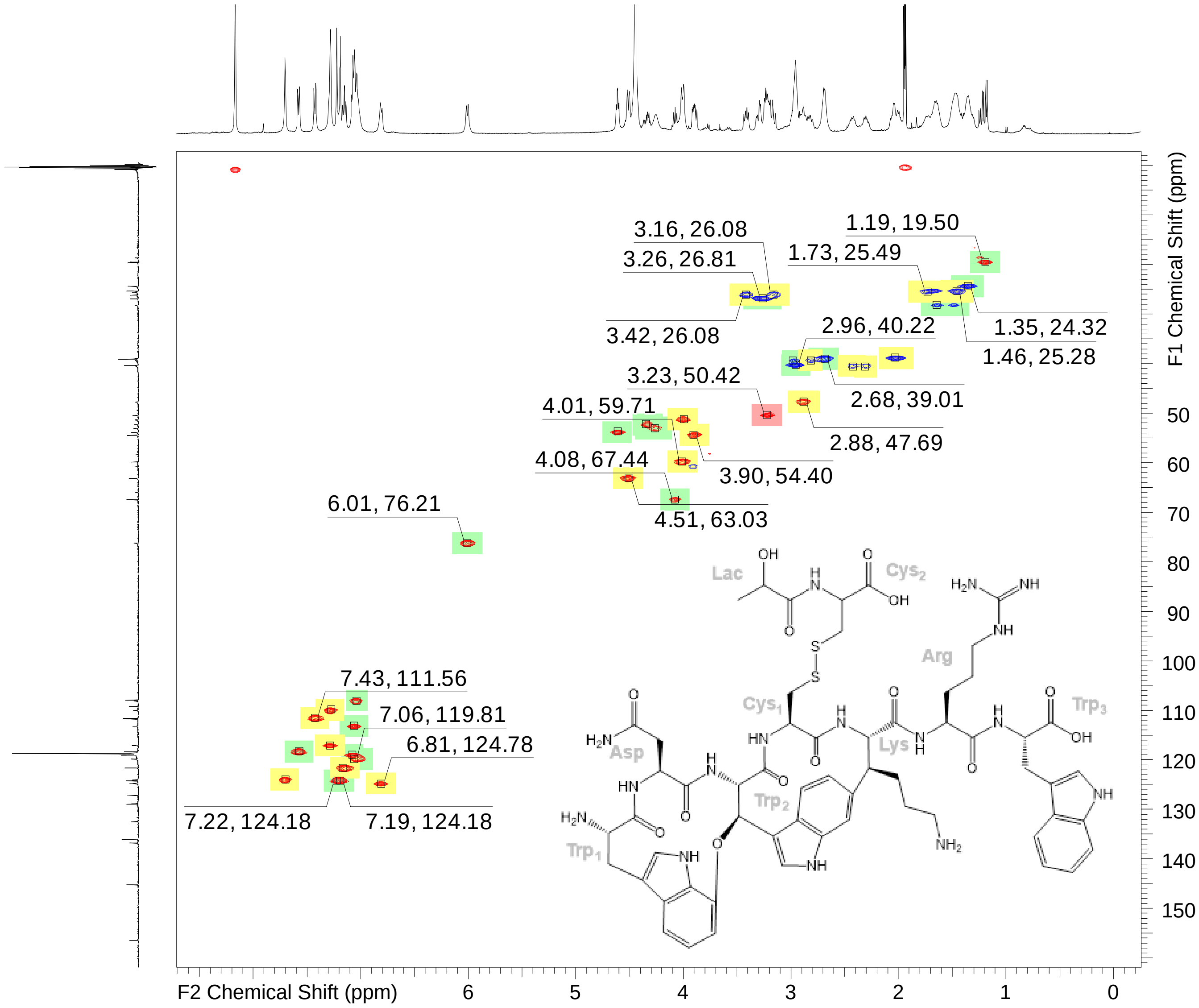

Figure S 83: HSQC spectrum of D32[R] in ACN/D2O + 1% FA at 35 °C and 500/125 MHz.

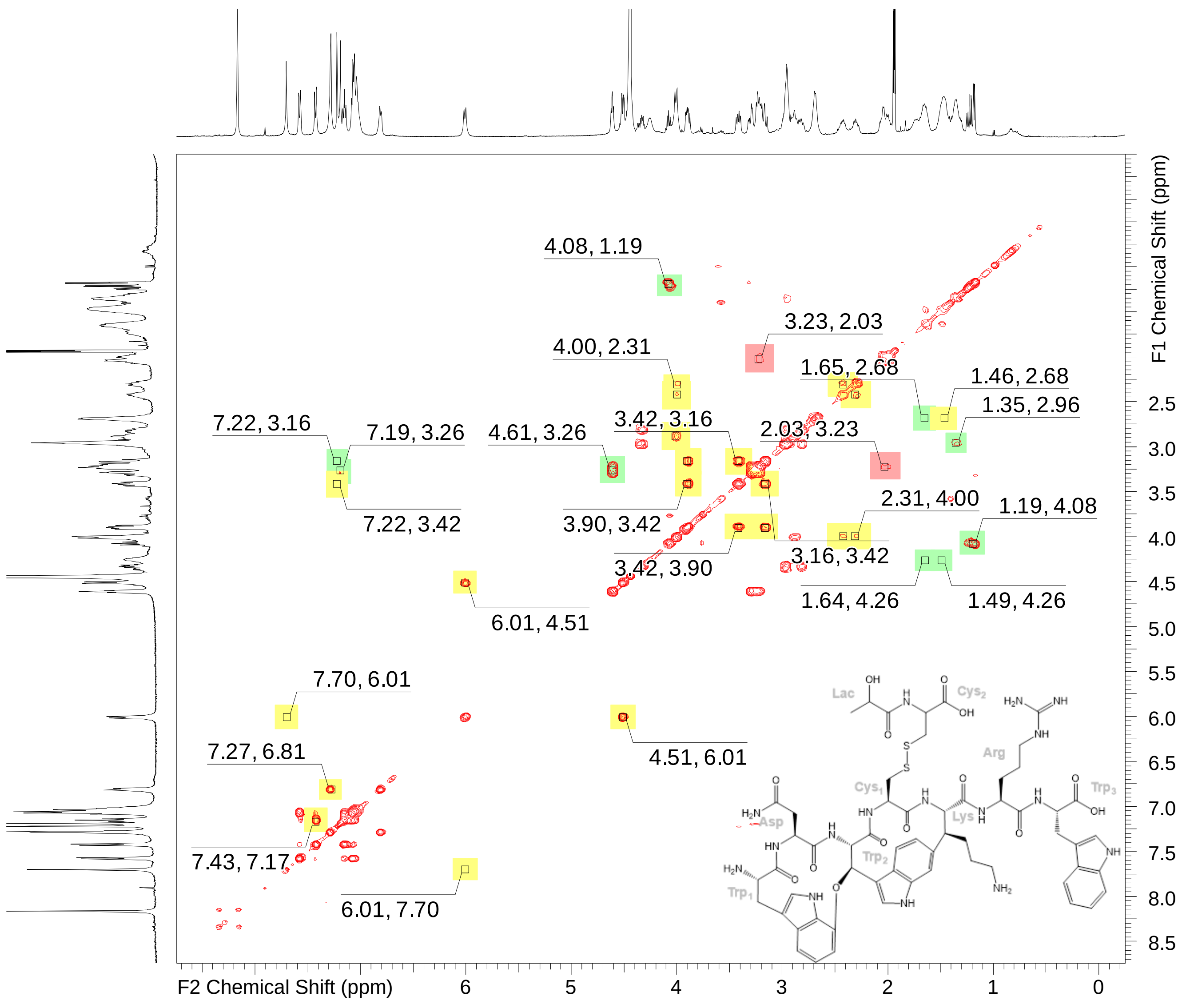

Figure S 84: COSY spectrum of D32[R] in ACN/D2O + 1% FA at 35 °C and 500 MHz.

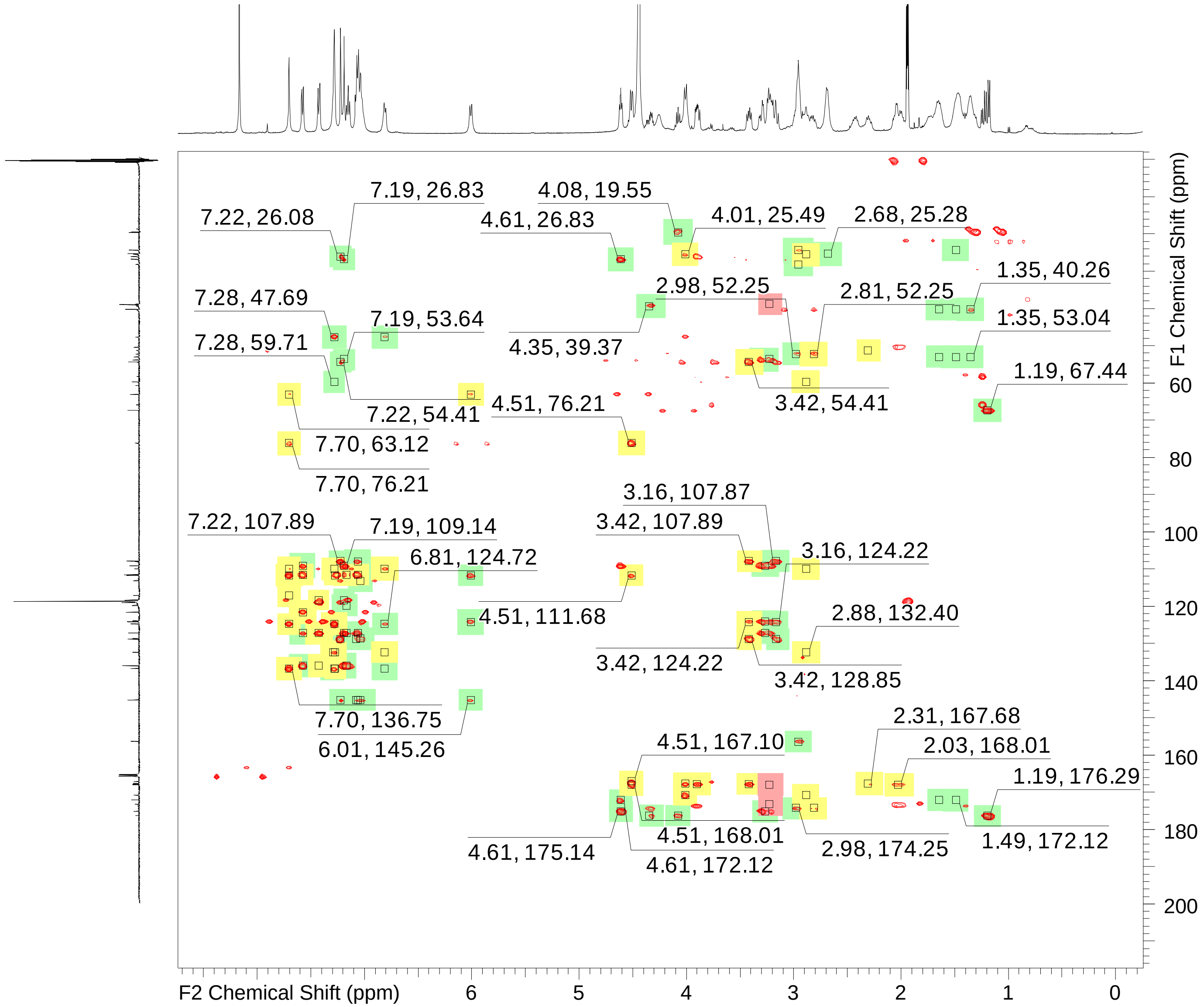

Figure S 85: HMBC spectrum of D32[R] in ACN/D2O + 1% FA at 35 °C and 500/125 MHz.

D9-6F

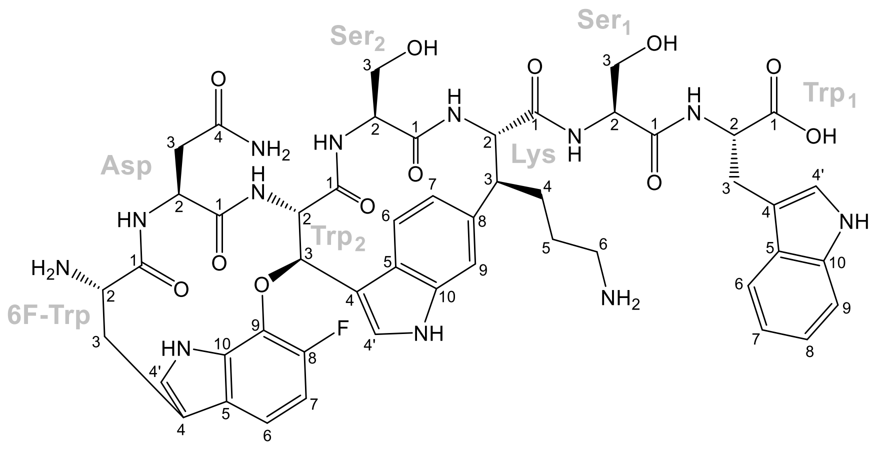

Table S 15: NMR spectroscopic data of D9-6F.

		NMR data in ACN/D_2O + 1% FA-d_4		
position	δ ^{13}C	δ ^{1}H, mult (*J* in Hz)	COSY correlations	HMBC correlations
Trp₁				
1	174.5	-	-	-
2	53.5	5.20, t (5.9)	3	1, 3, 4, *Ser*-1
3	26.8	3.80, m	2	1, 2, 3
4	108.9	-	-	-
4’	124.3	7.72, s	-	3, 4, 5, 9, 10
5	127.2	-	-	-
6	118.2	8.13, d (7.9)	7	8, 10
7	119.1	7.62, m	6, 8	5, 9
8	121.7	7.71, m	7, 9	6, 10
9	111.6	7.99, d (8.4)	8	5, 7
10	136.1	-	-	-
Ser₁				
1	170.4	-	-	-
2	55.3	4.89, m	3	1, 3, *Lys*-1
3	61.0	4.22, m	2	1, 2
Lys				
1	171.2	-	-	-
2	59.9	4.57, d (10.2)	3	1, 3, 4, *Ser₂*-1, *Trp₂*-8
3	48.0	3.38, m	2, 4	1, 2, 4, 5, *Trp₂*-7, *Trp₂*-8, *Trp₂*-9
4	25.3	2.22, 2.03, m	3, 5	3, 5, 6, *Trp₂*-8
5	25.4	2.14, m	4, 6	3, 4, 6
6	39.1	3.25, m	5	4, 5

NMR data in ACN/D_2O + 1% FA-d_4				
position	δ ^{13}C	δ ^{1}H, mult (*J* in Hz)	COSY correlations	HMBC correlations
Ser$_2$				
1	167.7	-	-	-
2	53.9	4.36, m	3	1, 3, *Trp$_2$*-1
3	61.8	3.54, dd (11.3, 6.4)	2	1, 2
Trp$_2$				
1	167.8	-	-	-
2	62.9	5.14, d (9.1)	3	1, 3, 4, *Asp*-1
3	76.0	6.54, bd (8.9)	2, 4‘	2, 4, 4‘, *Trp$_3$*-9
4	111.7	-	-	-
4‘	124.0	8.27, s	3	3, 4, 5, 10
5	124.8	-	-	-
6	117.0	7.79, d (7.6)	7	4, 8, 10
7	124.8	7.33, d (7.3)	6	5, 9, *Lys*-3
8	132.6	-	-	-
9	110.4	7.83, s	-	5, 7, *Lys*-3
10	136.9	-	-	-
Asp				
1	168.5	-	-	-
2	50.4	3.89, m	3	1, 3, 4, *Trp$_3$*-1
3	38.9	2.60, t (7.4)	2	1, 2, 4
4	173.1	-	-	-
6F-Trp				
1	167.5	-	-	-
2	54.4	4.44, m	3	1, 3
3	25.9	3.65, 3.96, m	2	1, 2, 4, 4‘, 5
4	108.0	-	-	-
4‘	124.7	7.75, s	-	3, 4, 5, 9, 10
5	124.8	-	-	-
6	114.0, d (*J* = 9.3)	7.59, m	7	4, 10
7	109.5, d (*J* = 22.4)	7.44, m	6	5, 8, 9
8	149.4, d (*J* = 235.1)	-	-	-
9	130.9	-	-	-
10	130.3	-	-	-

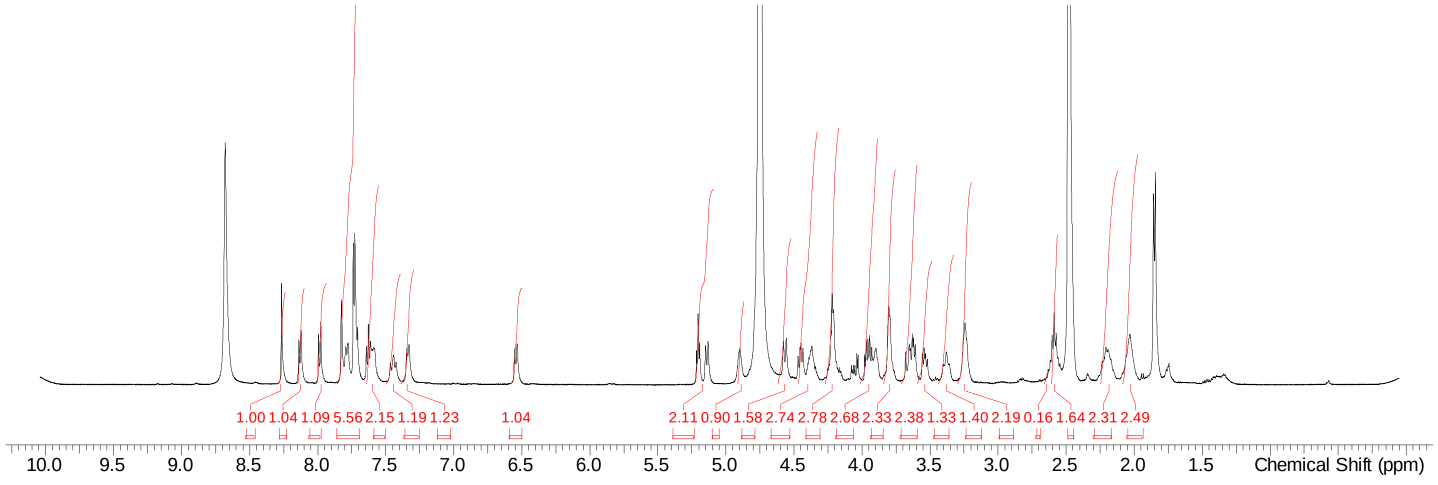

Figure S 86: ^{1}H spectrum of D9-6F in ACN/D2O + 1% FA at 45 °C and 500 MHz.

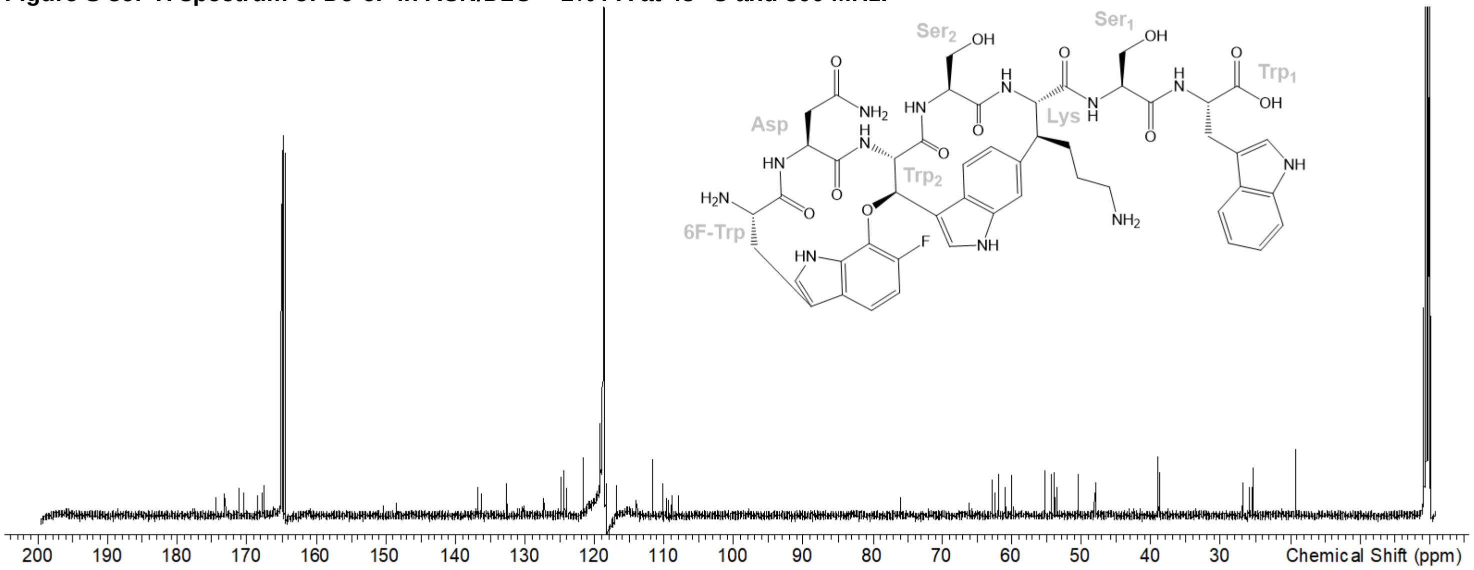

Figure S 87: ^{13}C spectrum of D9-6F in ACN/D2O + 1% FA at 45 °C and 125 MHz.

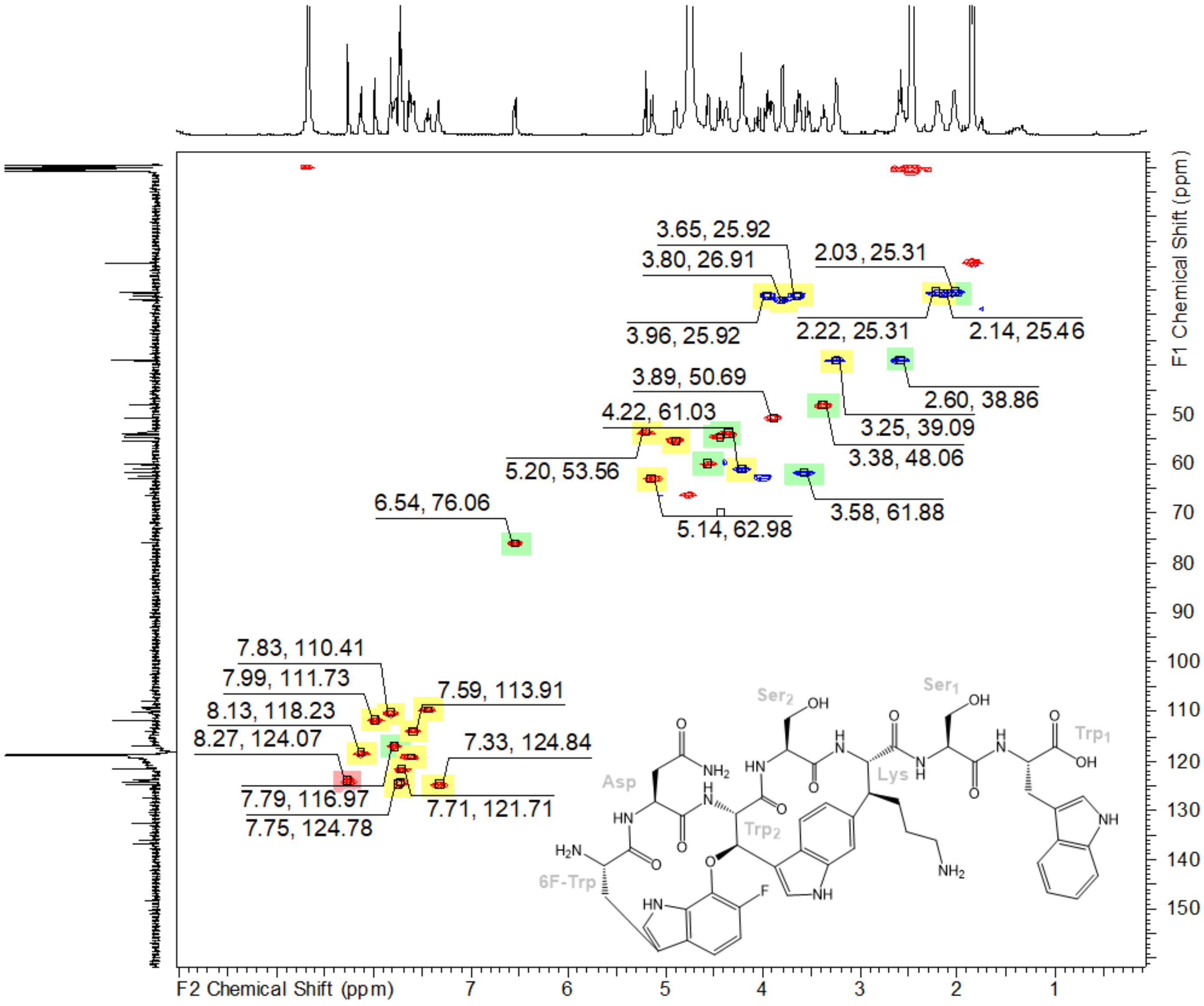

Figure S 88: HSQC spectrum of D9-6F in ACN/D2O + 1% FA at 45 °C and 500/125 MHz.

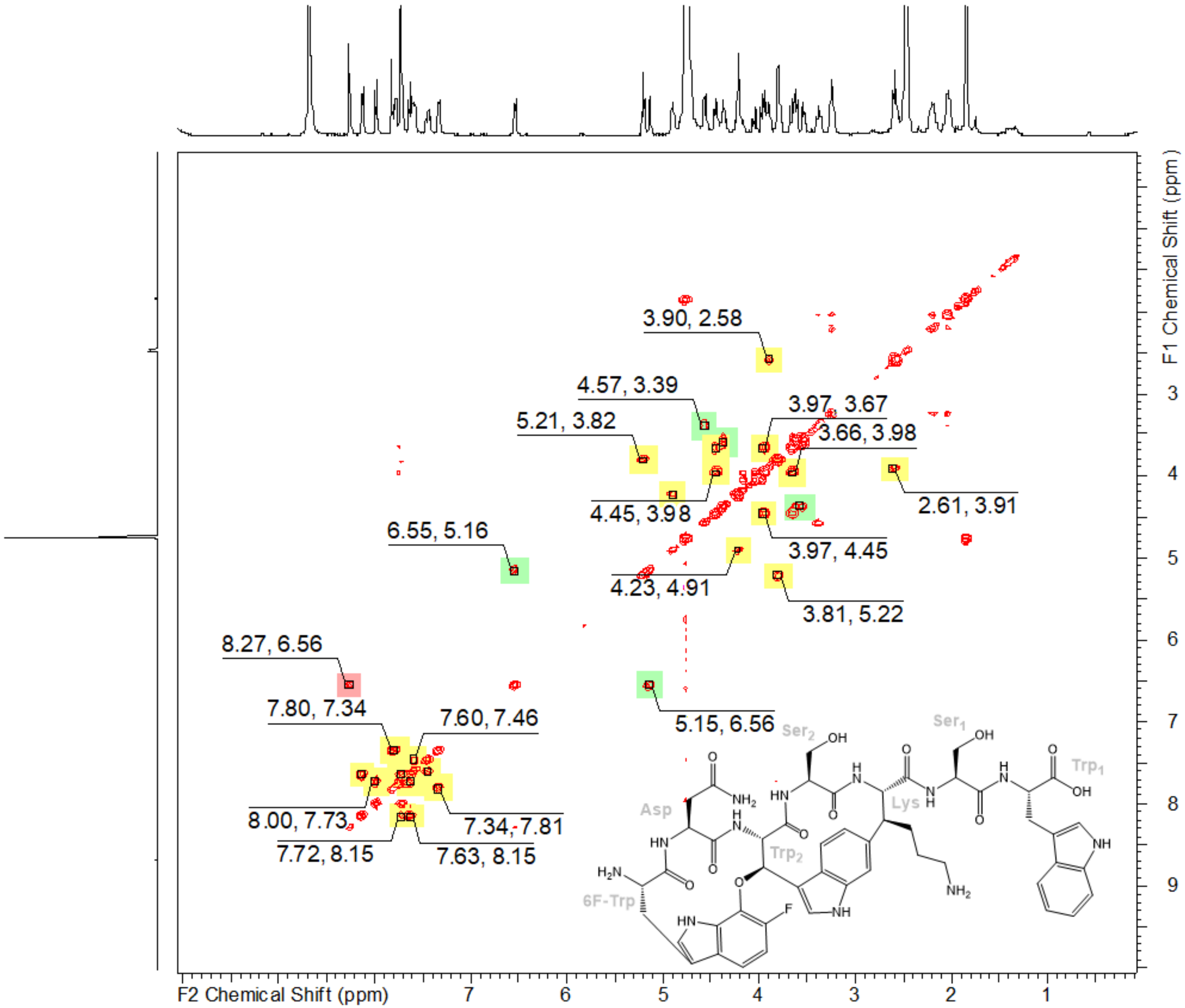

Figure S 89: COSY spectrum of D9-6F in ACN/D2O + 1% FA at 45 °C and 500/125 MHz.

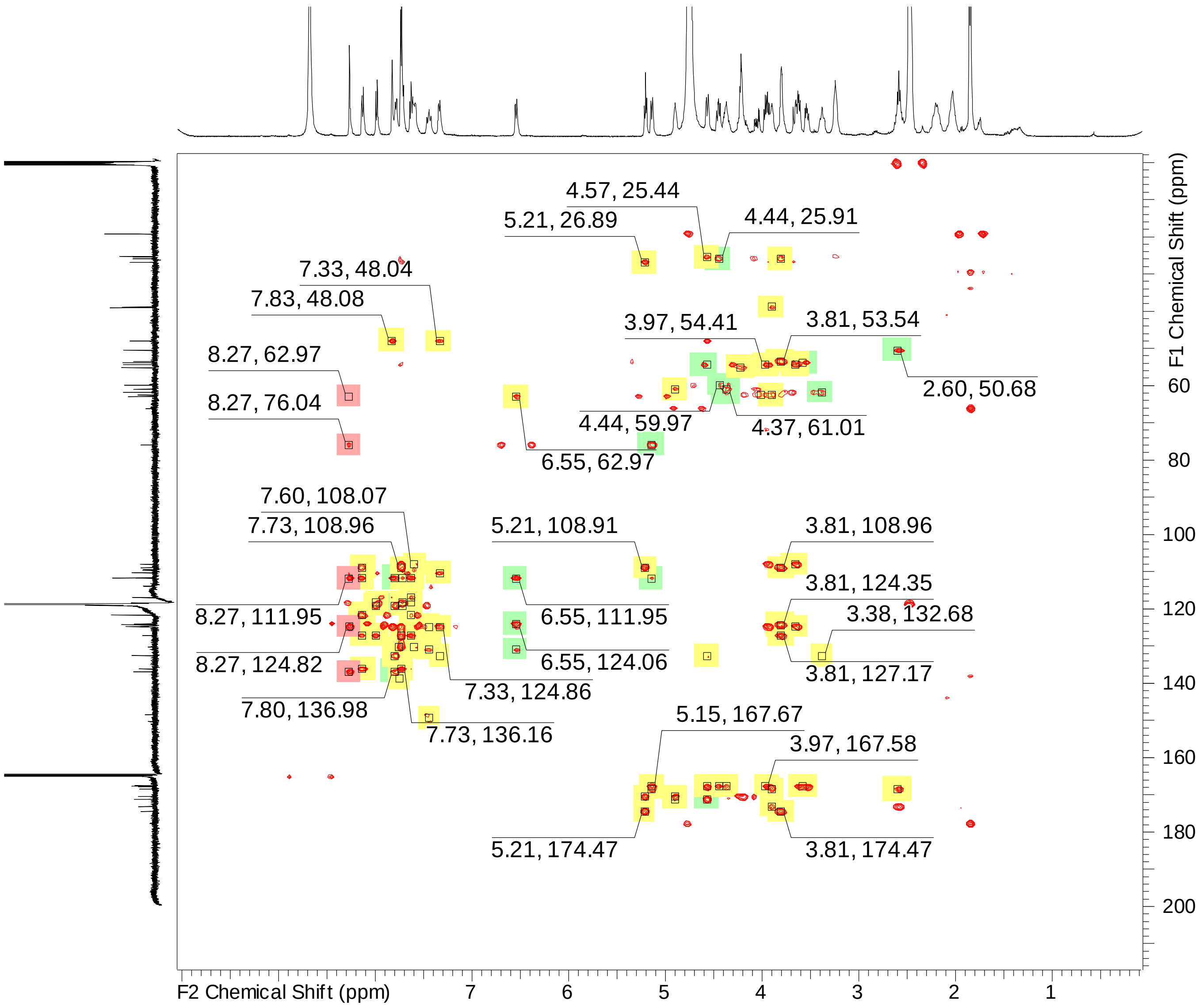

Figure S 90: HMBC spectrum of D9-6F in ACN/D2O + 1% FA at 45 °C and 500/125 MHz.

[1] S. Groß, F. Panter, D. Pogorevc, C. E. Seyfert, S. Deckarm, C. D. Bader, J. Herrmann, R. Müller, *Chem. Sci.* **2021**, *12*, 11882.
[2] K. H. M. E. Tehrani, H. Fu, N. C. Brüchle, V. Mashayekhi, A. Prats Luján, M. J. van Haren, G. J. Poelarends, N. I. Martin, *ChemComm* **2020**, *56*, 3047.
[3] C. B. Kimmel, W. W. Ballard, S. R. Kimmel, B. Ullmann, T. F. Schilling, *Dev. Dyn.* **1995**, *203*, 253.
[4] J. A. van Santen, G. Jacob, A. L. Singh, V. Aniebok, M. J. Balunas, D. Bunsko, F. C. Neto, L. Castaño-Espriu, C. Chang, T. N. Clark et al., *ACS Cent. Sci.* **2019**, *5*, 1824.
[5] H. Kaur, R. P. Jakob, J. K. Marzinek, R. Green, Y. Imai, J. R. Bolla, E. Agustoni, C. V. Robinson, P. J. Bond, K. Lewis et al., *Nature* **2021**, *593*, 125.
[6] S. H. W. Scheres, *J. Struct. Biol.* **2012**, *180*, 519.
[7] A. Punjani, J. L. Rubinstein, D. J. Fleet, M. A. Brubaker, *Nat Methods* **2017**, *14*, 290.
[8] E. F. Pettersen, T. D. Goddard, C. C. Huang, E. C. Meng, G. S. Couch, T. I. Croll, J. H. Morris, T. E. Ferrin, *Protein Sci.* **2021**, *30*, 70.
[9] P. D. Adams, R. W. Grosse-Kunstleve, L. W. Hung, T. R. Ioerger, A. J. McCoy, N. W. Moriarty, R. J. Read, J. C. Sacchettini, N. K. Sauter, T. C. Terwilliger, *Acta Crystallogr. D Biol Crystallogr* **2002**, *58*, 1948.
[10] T. I. Croll, *Acta Crystallogr. D Biol Crystallogr* **2018**, *74*, 519.
